# Glioma-intrinsic MAPK/ERK signaling promotes immunotherapy efficacy through T cell infiltration and interferon responses

**DOI:** 10.1038/s41467-026-74124-7

**Published:** 2026-06-25

**Authors:** Kwang-Soo Kim, Junyi Zhang, Víctor A. Arrieta, Crismita Dmello, Elena Grabis, Yahaya A. Yabo, Si Wang, Karl Habashy, Joseph Duffy, Junfei Zhao, Andrew Gould, Rishi Jain, Li Chen, Jian Hu, Irina Balyasnikova, Dhan Chand, Daniel Levey, Peter Canoll, Wenting Zhao, Peter A. Sims, Raul Rabadan, Surya Pandey, Bin Zhang, Pouya Jamshidi, Catalina Lee-Chang, Dieter Henrik Heiland, Adam M. Sonabend

**Affiliations:** 1 Department of Neurological Surgery, Feinberg School of Medicine, Northwestern University, Chicago, IL USA; 2 Northwestern Medicine Malnati Brain Tumor Institute of the Lurie Comprehensive Cancer Center, Feinberg School of Medicine, Northwestern University, Chicago, IL USA; 3 Microenvironment and Immunology Research Laboratory, Friedrich-Alexander Universität Nürnberg-Erlangen, Erlangen, Germany; 4 Translational Neurosurgery, Friedrich-Alexander Universität Nuremberg-Erlangen, Erlangen, Germany; 5 Department of Neurosurgery, University Hospital Erlangen, Friedrich-Alexander University Erlangen Nuremberg, Erlangen, Germany; 6 Department of Neurosurgery, Medical Center-University of Freiburg, Freiburg, Germany; 7 Department of Systems Biology, Columbia University, New York, NY USA; 8 Department of Biomedical Informatics, Columbia University, New York, NY USA; 9 Department of Cancer Biology, The University of Texas MD Anderson Cancer Center, Houston, TX USA; 10 The University of Texas MD Anderson Cancer Center UTHealth Graduate School of Biomedical Sciences, Houston, TX USA; 11 Agenus Inc., Lexington, MA USA; 12 Herbert Irving Comprehensive Cancer Center, Columbia University Irving Medical Center, New York, NY USA; 13 Department of Pathology and Cell Biology, Columbia University Irving Medical Center, New York, NY USA; 14 Department of Biochemistry & Molecular Biophysics, Columbia University, New York, NY USA; 15 Department of Hematology and Oncology, Feinberg School of Medicine, Northwestern University, Chicago, IL USA; 16 Department of Pathology, Feinberg School of Medicine, Northwestern University, Chicago, IL USA

**Keywords:** CNS cancer, Immunization

## Abstract

Glioblastoma (GBM) remains a formidable challenge in neuro-oncology, with immune checkpoint blockade (ICB) only showing efficacy in some patients, while the mechanisms governing therapeutic responsiveness are poorly defined. Although MAPK/ERK signaling correlates with survival following ICB, its causal role and mechanisms underlying tumor immunogenicity remain unclear. Here, we perform in vivo kinome-wide CRISPR/Cas9 screens in murine gliomas where we identify RAF-MEK-ERK axis as the strongest modulators of glioma susceptibility to anti-programmed cell death protein 1 (anti-PD-1) therapy and CD8^+^ T cell recognition. Experimentally-induced ERK phosphorylation (p-ERK) enhances survival after anti-PD-1 and anti-CTLA-4 therapy, leading to durable antitumor immunity upon rechallenge. Additionally, glioma cell p-ERK promotes increased interferon responses and T cell infiltration. Notably, BRAF/MEK inhibition disrupts interferon programs and tumor-microglia interactions in *BRAF*^*V600E*^ ex vivo in human GBM/brain slice cultures. Our findings elucidate that tumor-intrinsic MAPK/ERK promotes immunotherapy response, interferon responses, T cell tumor infiltration, and GBM cell-microglia interactions.

## Abstract (web summary)

Activation of the MAPK/ERK signaling pathway is known to influence glioma biology. Here the authors report that MAPK/ERK signaling activation drives interferon responses in glioma cells, enhancing anti tumor immunity and response to immune checkpoint blockade.

## Introduction

Glioblastoma (GBM), the most common and malignant primary brain tumor in adults, is characterized by pronounced genetic and molecular heterogeneity^1,2^. This diversity not only drives oncogenic programs, such as uncontrolled cell proliferation, but also results in variability in the activation of key signaling pathways, such as MAPK and PI3K/AKT/mTOR^3,4^. The complexity of GBM biology is further compounded by numerous genetic and epigenetic alterations that can modulate these pathways, producing a wide spectrum of molecular profiles^5^. Importantly, this complex molecular landscape extends beyond tumor cells themselves, shaping the tumor microenvironment, which plays a crucial role in GBM progression. Tumor-associated macrophages and microglia (TAM) constitute up to 30–40% of the tumor volume^6,7^, exhibiting a phenotypic spectrum shaped by tumor cell-derived cues that are linked to the underlying molecular heterogeneity^8,9^. Such dynamic interplay between tumor cells and their microenvironment contributes to the many challenges of treating GBM.

The complex molecular landscape of GBM significantly influences clinical outcomes, shaping how individual tumors respond to treatment^1,9–12^. In the setting of immunotherapy, tailoring regimens to the specific molecular and immunologic features of subsets of tumors may reveal efficacy in selected cases, even though traditional randomized controlled trials in unselected populations may not show overall survival benefits^13^. Anti-PD-1 (aPD-1) immunotherapy exemplifies this challenge; while some cases demonstrate durable responses and prolonged survival, multiple randomized controlled trials in unselected patients have failed to meet the primary efficacy endpoint^13–17^.

We previously reported an association between mutations that activate the MAPK pathway (*PTPN11*/*BRAF*) and GBM susceptibility to aPD-1 therapy^12^. Notably, MAPK activation predicted positive therapeutic effect even in the absence of these mutations; elevated phosphorylation of the ERK protein (p-ERK), a downstream effector of MAPK signaling, predicted overall survival in GBM patients undergoing aPD-1 and anti-CTLA-4 (aCTLA-4) therapy across several independent cohorts^11,18^. Tumors with elevated p-ERK^+^ tumor cell density exhibited a distinct microglial phenotype, characterized by the spatial proximity of microglia to p-ERK^+^ tumor cells and the expression of MHC class II molecules^11^. However, the causal relationship between MAPK signaling, this microglial phenotype, and enhanced immunotherapy responsiveness remains unsolved. Moreover, the mechanisms by which MAPK-active tumor cells become more immunogenic or modulate adjacent TAM are not well understood.

In this study, we leverage two large in vivo CRISPR knockout (KO) screens, mouse glioma models, in vitro experiments, and reverse translation data derived from analyses of human tumors to investigate the impact of tumor cell-intrinsic p-ERK and MAPK signaling on the tumor cell immunogenicity, microglial phenotype, and susceptibility to ICB. By elucidating the role of MAPK signaling in shaping tumor immunogenicity and the microenvironment, our findings provide a foundation for more precise and effective immunotherapy strategies tailored to the molecular profiles of GBM.

## Results

### In vivo kinome-wide CRISPR/Cas9 screens identify MAPK/ERK signaling as a regulator for susceptibility to CD8^+^ T cells and anti-PD-1 immunotherapy

**Fig. 1:**
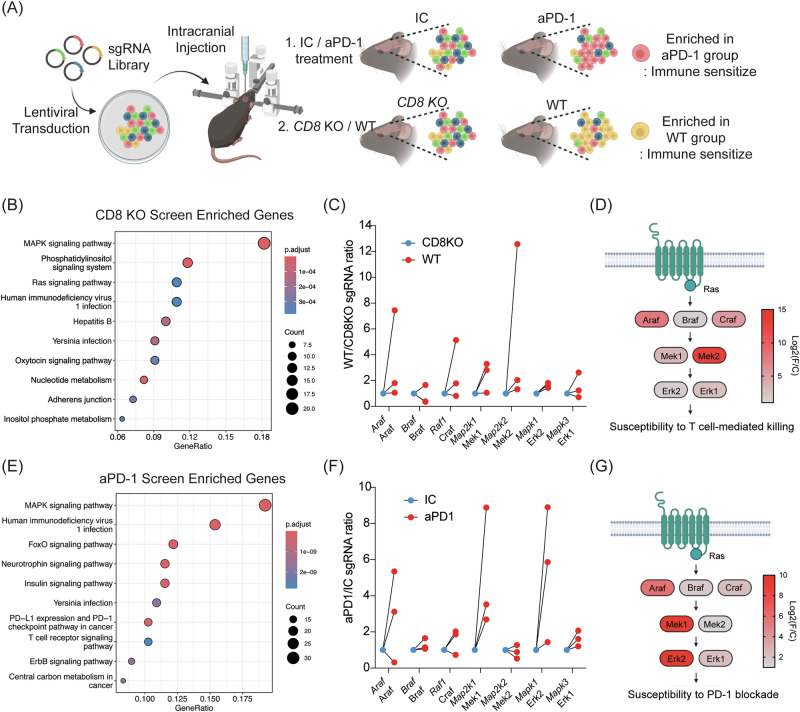
In vivo kinome-wide CRISPR/Cas9 screens identify MAPK/ERK signaling as a regulator for susceptibility to CD8^+^ T cells and anti-PD-1 immunotherapy. **A** Schematic illustration of in vivo CRISPR/Cas9 screening. SgRNA library was transduced to GL261 mouse glioma cells, and the cells were injected intracranially. Comparisons were (1) IC (*n* = 11) vs. aPD-1 (*n* = 12), (2) wildtype (*n* = 12) vs. *Cd8* KO mice (*n* = 20). Created in BioRender. Kim, K. (2026) https://BioRender.com/9l4vkhb. **B** Pathway enrichment analysis of significant genes identified in the kinome-wide CRISPR/Cas9 screens comparing wildtype and *Cd8* KO mice. **C** Fold changes of sgRNAs targeting MAPK/ERK pathway genes. Comparing wildtype and *Cd8* KO mice, 3 sgRNAs are depicted. **D** Detailed diagrams of the MAPK signaling cascade, indicating log fold changes in sgRNA enrichment. Log_2_(fold change) was calculated based on the top-performing sgRNA depleted in *Cd8* KO mice. **E** Pathway enrichment analysis from CRISPR/Cas9 screening comparing mice that received IC and aPD-1. **F** Fold changes of sgRNAs targeting MAPK/ERK pathway genes. Comparing the IC and the aPD-1 group, 3 sgRNAs are depicted. **G** Diagrams of the MAPK signaling cascade, indicating log fold changes in sgRNA enrichment. Log_2_(fold change) was calculated based on the top-performing sgRNA enriched in the aPD1-treated group. For (**B**, **E**), *P* values were adjusted for multiple comparisons, and color indicates adjusted *P* value (*p*.adjust). Dot size indicates gene count, and the x-axis indicates GeneRatio. The gene list of the kinome was used as background (denominator) to calculate the gene ontology enrichment. Source data are provided as a Source data file.

To investigate whether MAPK/ERK signaling contributes to immune cell recognition of glioma cells, we conducted kinome-wide CRISPR/Cas9 screens. GL261 glioma cells were transduced with a lentiviral sgRNA library titered to mostly result in the knockout (KO) of a single kinase gene per tumor cell. These transduced cells were then implanted intracranially into mice (Fig. 1A). By comparing the implanted glioma cells into wild-type and *Cd8* KO mice, we identified kinase KO clones that were selected in the presence of CD8^+^ T cells in the host^19^, independent of the oncogenic properties of these genes. Gene ontology (GO) analysis identified the MAPK pathway as the most significantly enriched kinase pathway in CRISPR KO clones selected by CD8^+^ T cells in wild-type mice compared to *Cd8* KO hosts (Fig. 1B). Among MAPK-related genes, *Map2k2* (encoding Mek2) and *Araf*, both upstream of Erk phosphorylation, showed the highest enrichment (Fig. 1C, D).

We next examined whether these kinases, identified as critical for glioma recognition by CD8^+^ T cells, also conferred susceptibility to ICB. To this end, we performed a similar CRISPR/Cas9 screen in which transduced glioma cells were implanted into wild-type mice treated with aPD-1 or with an isotype control (IC) antibody. In this screen, the MAPK signaling pathway once again emerged as the most enriched among all KO clones selected by aPD-1 treatment (Fig. 1E). Within the MAPK pathway, genes, such as *Araf*, *Raf1*, *Map2k1* (encoding Mek1), and *Mapk1* (encoding Erk2), showed the highest enrichment under aPD-1 treatment (Fig. 1F, G), all involved in ERK activation. Consistent with previous reports, *Cd8* KO exhibited significantly reduced survival, while ICB treatment showed no survival difference in the GL261 mouse model (Supplementary Fig. 1A, B)^19,20^. A scatter plot summarizing the whole kinome CRISPR/Cas9 screen results from the *Cd8* KO screen is available in Supplementary Fig. 1C (modified from Dmello et al.)^19^, and for the aPD-1 screen in Supplementary Fig. 1D. These gene hits were identified through a multilevel variant analysis, which first normalized each group to the same baseline control before comparing the control and treatment groups using MAGEcK maximum likelihood estimation (MLE) workflow. This approach mitigates potential false positives arising from time-selection pressure. Further, we conducted a random permutation test, selecting a gene set of the same size as our significant hits from the BRIE library dataset for pathway enrichment analysis. The resulting analysis did not identify the MAPK pathway as significantly enriched (Supplementary Fig. 2).

### MAPK activation enhances glioma susceptibility to aPD-1 therapy and promotes durable anti-tumoral immunity

**Fig. 2:**
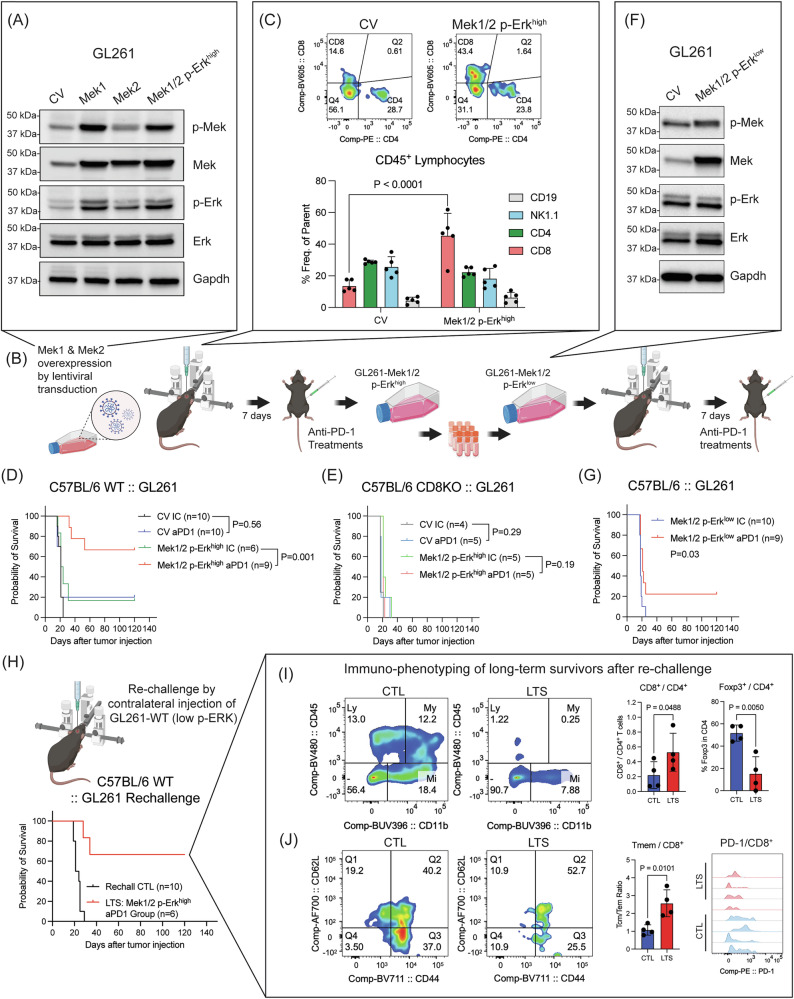
MAPK activation enhances glioma susceptibility to aPD-1 therapy and promotes durable anti-tumoral immunity. **A**, **B** Schematic illustration of cell line generation and in vivo survival studies for GL261 Mek1/2 overexpression stable cell line was established by lentiviral transduction and confirmed by immunoblotting to confer Erk1/2 phosphorylation. Representative western blot from three independent experiments. For treatment with ICB antibodies, treatment started 1 week after tumor implantation and was delivered intravenously 4 times every 3 or 4 days. Created in BioRender. Kim, K. (2026) https://BioRender.com/9zct88w. **C** Flow cytometry-based immune cell infiltration analysis. After 20 days of tumor cell infusion, tumor-infiltrating lymphocytes were analyzed comparing control GL261 tumor (*n* = 5) and GL261 Mek1/2 p-Erk^high^ tumor (*n* = 5). Bar graph shows mean ± s.d. **D** Kaplan–Meier survival curve of GL261 CV (IC, *n* = 10 and aPD-1, *n* = 10) and GL261 Mek1/2 p-Erk^high^ (IC, *n* = 6 and aPD-1, *n* = 9) treated with IC or aPD-1 antibody, conducted in wildtype C57BL/6 mice. **E** Kaplan–Meier survival curve of control GL261 CV (IC, *n* = 4 and aPD-1, *n* = 5) and GL261 Mek1/2 p-Erk^high^ (IC, *n* = 5 and aPD-1, *n* = 5) treated with IC or aPD-1 antibody, conducted in *Cd8* KO C57BL/6 mice. **F** Western blot analysis of Erk and Mek phosphorylations and expressions after several passages and freeze/thaw cycles. The cell line was referred to as GL261 Mek1/2 p-Erk^low^. Representative western blot from three independent experiments. **G** Kaplan–Meier survival curve of control GL261 tumor and GL261 Mek1/2 p-Erk^low^ treated with IC (*n* = 9) or aPD-1 (*n* = 10), conducted in wildtype C57BL/6 mice. **H** The mice cohort from GL261 Mek1/2 p-Erk^high^, the long-term survivors received contralateral injection of wildtype GL261 cell infusion on day 120 post tumor cell injection (control, *n* = 10 and long-term survivors, *n* = 6). Created in BioRender. Kim, K. (2026) https://BioRender.com/9zct88w. **I** Immune landscape after 120 days of the rechallenge. Compared with brand-new tumors (CTL, *n* = 4), brains from long-term survivors (LTS, *n* = 4) were analyzed for immune phenotyping. Flow cytometry data showing percoll-enriched cells (left). Bar graphs show CD8^+^ and CD4^+^ T cell ratio (middle), Foxp3^+^ cells in the CD4^+^ T cell population (right). **J** Flow cytometry data showing expression of CD44 and CD62L in CD8^+^ T cells (left). Bar graph showing central memory T cell (CD44^+^CD62L^+^) to effector memory T cell (CD44^+^CD62L^−^) ratio (middle). Flow cytometry data showing PD-1 expression in CD8^+^ T cells (right). Data are presented as mean ± s.d. The *P* values for survival studies were generated from a log-rank test. Statistical analysis for group comparison was done using an unpaired two-tailed T test. Source data are provided as a Source data file.

To further explore the causal link between MAPK signaling in glioma cells and anti-tumoral immunity, we overexpressed *Mek1* and *Mek2* in GL261 mouse glioma cells (GL261 Mek1/2) via lentiviral-induction, which led to increased Erk phosphorylation (GL261 Mek1/2 p-Erk^high^; Fig. 2A, B). Intracranial implantation of GL261 Mek1/2 p-Erk^high^ cells resulted in tumors with higher CD8^+^ T cell infiltration compared to controls (Fig. 2C). To further validate the role of MAPK signaling in promoting CD8^+^ T cell infiltration, we used a CT-2A murine glioma model, and successful Erk phosphorylation was confirmed via Western blot (Supplementary Fig. 3A). Similar to GL261 glioma models, CT-2A Mek1/2 p-Erk^high^ tumors exhibited increased T cell infiltration compared to controls, as demonstrated by flow cytometry-based analysis (Supplementary Fig. 3B, C) and multiplex immunofluorescence staining (Supplementary Fig. 3D, E). This observation was consistent with our earlier finding that KO clones for *Mek1* and *Mek2* were selected in the presence of CD8^+^ T cells (Fig. 1C, D). The flow cytometry gating strategy used to quantify tumor-infiltrating T cells is shown in Supplementary Fig. 4A. Tumors derived from GL261 Mek1/2 p-Erk^high^ cells exhibited marked susceptibility to aPD-1 immunotherapy, with over 60% of the mice achieving long-term survival (>120 days, *P* = 0.001, Fig. 2D). This effect was CD8^+^ T cell-dependent, as *Cd8* KO hosts bearing GL261 Mek1/2 p-Erk^high^ tumors showed no survival benefit from aPD-1 treatment (Fig. 2E).

Interestingly, after multiple freeze-thaw cycles and several passages in culture, GL261 Mek1/2 cells maintained overexpression of *Mek1* and *Mek2* but lost ERK phosphorylation (Fig. 2F). When implanted intracranially, these cells with diminished p-Erk levels (GL261 Mek1/2 p-Erk^low^) showed reduced susceptibility to aPD-1 therapy, with no increase in median survival compared to IC-treated mice and only 22.2% of mice achieving long-term survival (Fig. 2G), further supporting the specific role of MAPK activation in immunotherapy response.

Building on these findings, we examined the durability of anti-tumoral immunity by re-challenging long-term survivors with parental GL261 cells implanted on the contralateral side of the brain. Of note, four out of six mice rejected the secondary tumors without any additional therapy (Fig. 2H), suggesting that the anti-tumoral immune response was related to MAPK activation and directed against endogenous tumor antigens rather than introduced transgenes. Immune phenotyping of long-term survivors revealed increased CD8/CD4 T cell ratio (*P* = 0.0488), decreased intra-tumoral FoxP3^+^ regulatory T cells (Tregs) (*P* = 0.0050, Fig. 2I), a higher ratio of central memory to effector memory CD8^+^ T cells (*P* = 0.0101), and reduced PD-1 expression on infiltrating CD8^+^ T cells (Fig. 2J). For the analyses shown, CD8^+^ or CD4^+^ T cells were first gated from the live single-cell lymphocyte population, and the indicated immune phenotypes were then quantified within the corresponding CD8^+^ or CD4^+^ T cell subsets (Supplementary Fig. 4A).

We further validated these findings in a transgenic-derived murine glioma model, QPP7, known for its susceptibility to immunotherapy^21^. These cells exhibited elevated p-Erk levels; therefore, we performed KO of *Erk1* (QPP7 Erk1KO) and *Erk2* (QPP7 Erk2KO), which led to reduced efficacy of aPD-1 therapy compared to non-target controls (Supplementary Fig. 5A). Median survival difference between IC-treated and aPD-1-treated groups was 7 days for QPP7 Erk1KO (aPD-1: 64 days; IC: 57 days) and 13 days for QPP7 Erk2KO (aPD-1: 57 days and IC: 44 days) (Supplementary Fig. 5B). In contrast, mice bearing QPP7 NTC tumors and treated with aPD-1 did not reach the median survival endpoint, further supporting a potential role of MAPK/ERK signaling in immunotherapy response (Supplementary Fig. 5B). Collectively, these data demonstrate that MAPK activation in glioma cells enhances susceptibility to aPD-1 therapy and promotes durable, tumor-specific immunity, primarily mediated by CD8^+^ T cells.

### MAPK/ERK activation improves the efficacy of Fc-enhanced anti-CTLA-4 immune checkpoint blockade in murine gliomas

Our CRISPR/Cas9 screens suggested that MAPK activation broadly impacts anti-tumoral immunity in gliomas. We next sought to determine whether this effect extends beyond aPD-1 therapy, and we examined the impact of MAPK activation on the efficacy of aCTLA-4 immunotherapy. While conventional aCTLA-4 therapy has not resulted in significant survival benefits for unselected GBM patients^17^, our prior work suggests that elevated tumor p-ERK levels are associated with prolonged survival in GBM patients treated with a combination of aPD-1 and aCTLA-4 blockade therapy^18,20^.

**Fig. 3:**
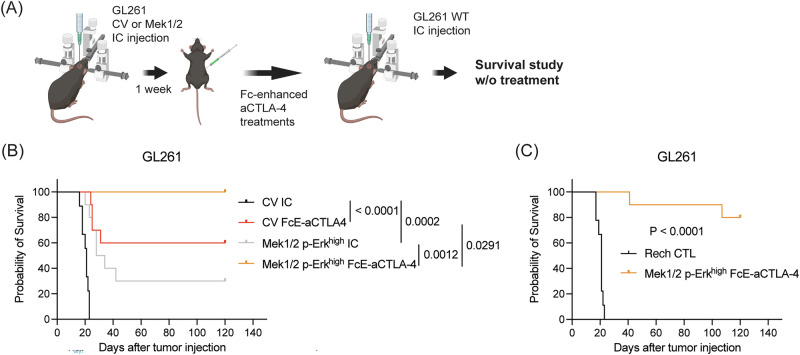
MAPK/ERK activation improves the efficacy of Fc-enhanced anti-CTLA-4 immune checkpoint blockade in murine gliomas. **A** Schematic illustration of the survival study using GL261 Mek1/2 p-Erk^high^ stable cells. For treatment with Fc-enhanced aCTLA-4, mice received the antibody starting 1 week after tumor implantation, administered intravenously 4 times every 3 or 4 days. Created in BioRender. Kim, K. (2026) https://BioRender.com/9zct88w. **B** Kaplan–Meier survival curve of GL261 CV (IC, *n* = 9 and Fc-enhanced aCTLA-4, *n* = 10) and GL261 Mek1/2 p-Erk^high^ (IC, *n* = 10 and Fc-enhanced aCTLA-4, *n* = 10) treated with IC or Fc-enhanced aCTLA-4 antibody. For treatment with ICB antibodies, treatment started 1 week after tumor implantation and was delivered intravenously 4 times every 3 or 4 days. **C** Kaplan–Meier survival curve of re-challenge with wildtype GL261 injection (GL261 Mek1/2 p-Erk^high^ Fc-enhanced aCTLA-4, *n* = 10; re-challenge control, *n* = 10). The *P* values were generated from a log-rank test. Source data are provided as a Source data file.

To investigate whether MAPK activation also contributes to glioma susceptibility to aCTLA4 therapy, we used a mouse surrogate of botensilimab, a next-generation Fc-enhanced (FcE) aCTLA-4 antibody designed to bind with high affinity to activating Fcγ receptors expressed by host immune cells and extend anti-tumor immunity against “cold” tumors^22–24^. We recently reported that this antibody is superior to conventional aCTLA-4 in glioma models^20^, and it is currently being evaluated in an ongoing clinical trial in GBM (NCT05864534). Given that the GL261 mouse glioma clone that we used as an in vivo model is resistant to both conventional and FcE-aCTLA-4^20^, we chose these tumors to evaluate whether the activation of the MAPK signaling led to improved efficacy of FcE-aCTLA-4 therapy (Fig. 3A). Mice bearing GL261 control vector (CV) exhibited mild improvement in efficacy of FcE-aCTLA-4 (*P* < 0.0001, 60% of long-term survivors). Yet, Mek1/2 p-ERK^high^ tumors treated with the FcE-aCTLA-4 antibody achieved a 100% survival rate (*P* = 0.0012), which was significantly better than that observed in CV tumors treated with the same antibody (*P* = 0.0291), and superior to Mek1/2 p-ERK^high^ tumors treated with an IC antibody (*P* = 0.0012) (Fig. 3B). Further, we performed a re-challenge experiment at day 120 to determine whether the therapeutic effect of FcE-aCTLA-4 in GL261 Mek1/2 p-Erk^high^ tumors was associated with durable anti-tumor immunity (Fig. 3A). Long-term survivor mice that were originally implanted with Mek1/2 p-Erk^high^ and initially treated with FcE-aCTLA-4, were re-challenged with wild-type GL261 injected into the contralateral hemisphere. Remarkably, 8/10 mice in the GL261 Mek1/2 p-Erk^high^ FcE-aCTLA-4 group remained tumor-free long term after the secondary intracranial injection, which compared favorably against a control cohort that was also implanted with wild-type GL261, which had not received previous immunotherapy treatment (*P* < 0.0001), indicating the establishment of a sustained, protective immune response (Fig. 3C). These findings highlight MAPK/ERK activation enhances glioma susceptibility to both aPD-1 and FcE-aCTLA-4 therapies for glioblastoma.

### MAPK/ERK pathway is associated with glioblastoma cell response to interferons

**Fig. 4:**
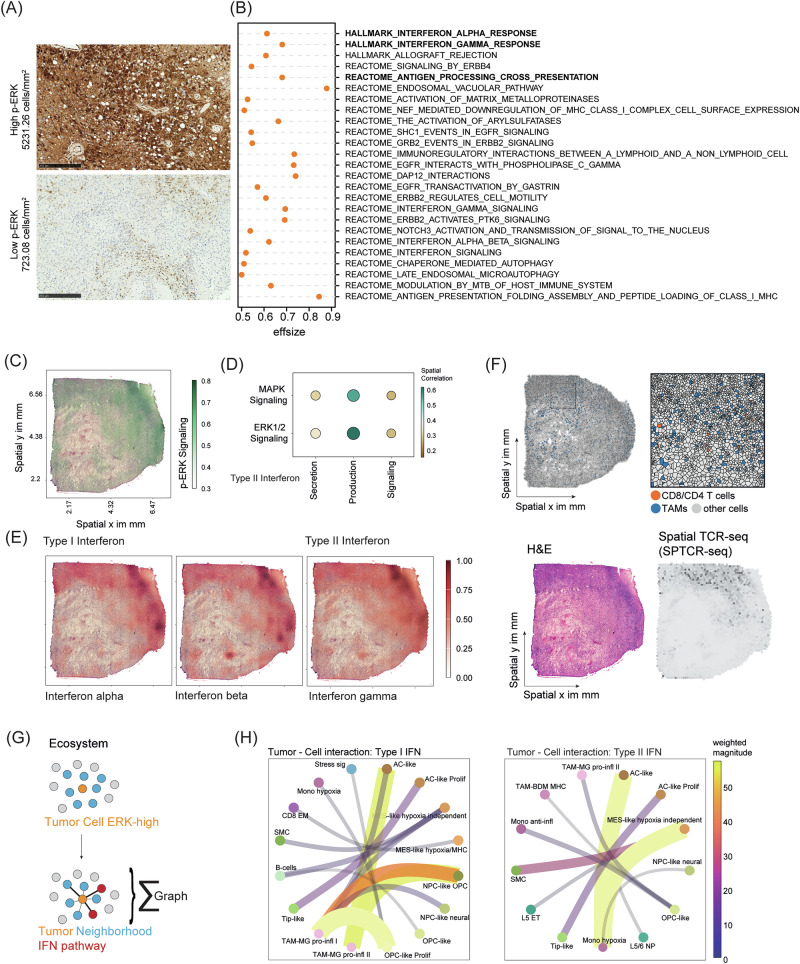
MAPK/ERK pathway is associated with glioblastoma cell response to interferons. **A** Representative immunohistochemistry images of high p-ERK (upper, *n* = 8) and low p-ERK (lower, *n* = 8) tumors. Scale bar, 250 µm. **B** Differentially expressed gene signatures of tumor cells from single-cell RNA sequencing comparing high (*n* = 8) and low (*n* = 8) p-ERK GBM tumors. **C** Example image of spatial multi-omic analysis of GBM tumor representing p-ERK signaling activity (*n* = 26, de novo IDH wildtype GBM samples). **D** Spatial correlation analysis of GBM tumors showing correlation between MAPK and ERK1/2 signaling and type II IFN signatures. **E** Representative images of spatial multi-omic analysis demonstrate type I and II interferon responses in GBM tumor. For **C**–**E**, the analysis was derived Visium Gene Expression slide platform. **F** Single-cell composition after deconvolution representing the location of T cells and TAMs (upper). H&E image demonstrates the histology of GBM tumor, and the image of spatial T cell receptor sequencing (SPTCR-seq) shows infiltrating T cells in GBM tumor (lower, *n* = 9). **G** Illustration of the MERFISH network analysis (*n* = 6). **H** Circular graph plot of the tumor-microenvironment proximity and IFN enrichment. Colors of the dots demonstrate the cell type, and the edges represent the mean enrichment of the IFN pathway (weighted magnitude).

A continuous paired analysis of single-cell RNA-sequencing (scRNA-seq) data from GBM patients with quantified p-ERK^+^ cell density (Fig. 4A) was performed to define the relationship between cell-intrinsic MAPK/ERK signaling and immune-related tumor phenotypes. This analysis revealed correlations between p-ERK^+^ cell density in tumor regions and gene expression of signaling pathways known to interact with MAPK/ERK, including receptor tyrosine kinase (RTK), WNT, AMP-activated protein kinase, and HIF-1α pathways (Supplementary Fig. 6)^3,25–27^. Notably, both continuous and dichotomous analyses identified significant correlations between p-ERK^+^ cell density and GO themes associated with interferon (IFN) responses (Fig. 4B and Supplementary Fig. 6).

To investigate the spatial relationship between ERK signaling, IFN responses, and T cell abundance, we analyzed spatially resolved transcriptomics across GBM samples^28^ (Fig. 4C). Spatial analysis revealed a correlation between the MAPK/ERK signaling pathway and type II IFN secretion, production, and signaling (Fig. 4D). Both type I and II IFN signatures were co-localized with regions of activated p-ERK signaling (Fig. 4E). Consistent with the results from the previous *Cd8* KO CRISPR/Cas9 screen (Fig. 1B) and in vivo immunophenotype analysis in two separate murine glioma models (Fig. 2C and Supplementary Fig. 3), p-ERK signaling was closely associated with T cell abundance, as determined by single-cell deconvolution and a separate validation dataset of SPTCR-seq (spatially resolved transcriptomics and TCR-seq) (Fig. 4F). We also performed ERK pathway signature enrichment analysis across GBM specimens to assess sample-wise differences (Supplementary Fig. 7A), which revealed that the IFN-α signature exhibited a strong correlation with the ERK pathway signature (Supplementary Fig. 7B).

We next validated these findings in GBM samples (*n* = 6) using spatial transcriptomics (MERFISH with single-cell resolution) (Supplementary Fig. 8A, B). The ERK signature was subsequently confirmed to be highly enriched in tumor cells, along with endothelia, vascular compartments, and neuronal populations (Supplementary Fig. 8C). The IFN-related pathways were predominantly enriched in the immune compartment, as well as in endothelial cells and mesenchymal-like tumor cells (Mes-like), consistent with recent reports^29^ (Supplementary Fig. 8D). Because the ERK signal and the IFN type I (alpha) expression appeared to be spatially correlated, we evaluated whether this reflected co-expression within the same cells or the proximity of distinct cell types. To address this, we performed spatial neighborhood analysis of the MERFISH single-cell spatial transcriptomic data, computing the first-hop neighborhoods of tumor cells with high ERK signature across all subgroups (Fig. 4G). Using single-cell spatial transcriptomics, we then quantified the probability of type I or II IFN (α/β or γ) pathway enrichment to be driven by direct cell–cell contacts within defined ecosystems. Type I IFN (in microglia) activation was markedly increased when tumor cells with a high ERK signature were in proximity to inflammatory microglia (Fig. 4H). Further analysis revealed that oligodendrocyte progenitor cell-like and neural progenitor cell (NPC)-like tumor subpopulations were the main contributors to this effect. In contrast, type II IFN activation in myeloid cells was predominantly associated with neighboring astrocyte (AC)-like and mesenchymal (MES)-like tumor populations (Fig. 4H).

Analysis of public RNA-seq data from patients who received either adjuvant or neoadjuvant PD-1 blockade^15^ suggested that high ERK signaling and corresponding IFN signaling were associated with improved survival (Supplementary Fig. 9A, B). These findings collectively demonstrate an association between MAPK/ERK activation, IFN responses, and T cell infiltration in GBM, providing mechanistic insights into the enhanced immunotherapy responsiveness observed in tumors with high MAPK/ERK activity.

### MAPK/ERK pathway drives interferon responses in glioma

**Fig. 5:**
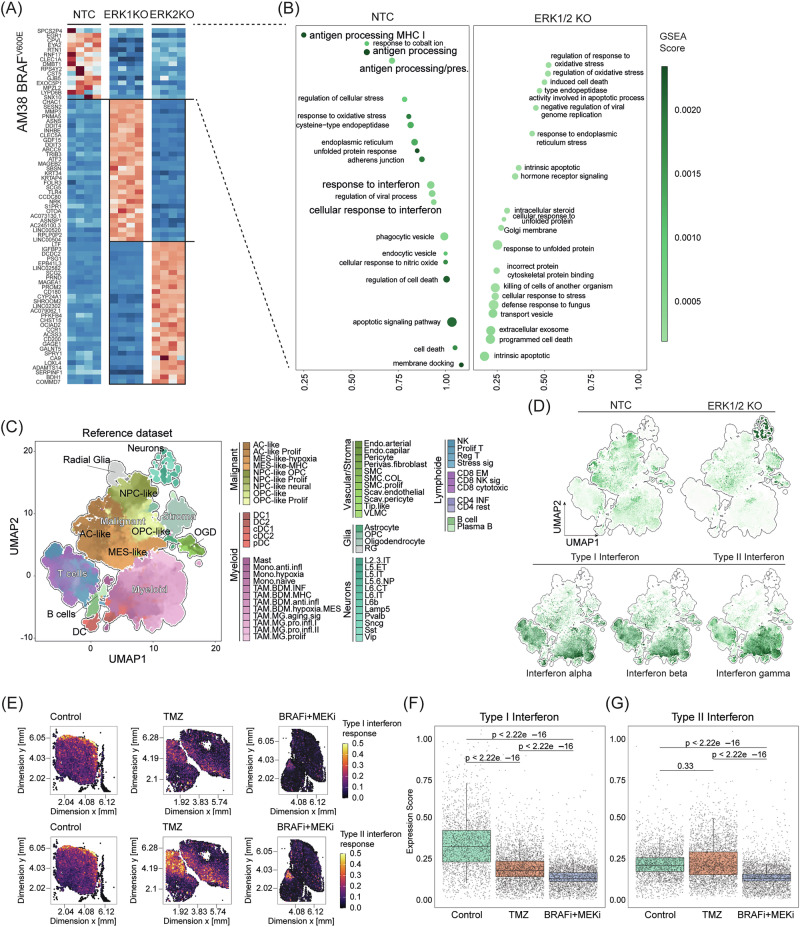
MAPK/ERK pathway drives interferon responses in glioma. **A** Bulk RNA-seq analysis of human GBM cell line AM38 NTC and AM38 ERK1/2KO. Heatmap displaying differential gene expression across four biologically independent samples per group (*n* = 4). NTC vs. ERK1/2KO is focused for further analysis. **B** Gene set enrichment analysis comparing AM38 NTC and AM38 ERK1/2KO (*n* = 4). **C** Uniform manifold approximation and projection dimensional reduction of the GBmap reference dataset. Each color indicates the different cell types. **D** Gene signatures from AM38 NTC (*n* = 4) and ERK1/2KO (*n* = 4) are projected on the GBmap reference dataset (top)^33^. Signatures of interferon alpha, beta, and gamma in the GBmap reference dataset (bottom). **E** Spatial transcriptomic and scRNA-seq analysis of slice culture of *BRAF*^*V600E*^ GBM tumor treated with TMZ or BRAFi/MEKi. One *BRAF*^*V600E*^ GBM patient with 6 slices per condition (control, TMZ, trametinib + dabrafenib, trametinib + vemurafenib, trametinib + encorafenib), for a total of 30 quantified slices. Spatial images of type I (upper) and II (lower) interferon signatures in slice culture samples treated with TMZ or BRAFi/MEKi. Differential analysis of interferon type I (**F**) and II (**G**) signatures comparing control, TMZ, and BRAFi/MEKi-treated slice culture. **E**–**G** were derived from spatial transcriptomic analyses using the Visium Gene Expression slide platform. For the bar plots, statistical significance was determined using an unpaired Wilcoxon test performed directly in R show median (centre line), interquartile range (box bounds). Every data point in these barplots represents one spatial spot from the corresponding sampling group.

To investigate the causal relationship between MAPK/ERK signaling and the observed cancer cell phenotypes across tumors, RNA-seq data from AM38, a human GBM cell line with *BRAF*^*V600E*^ mutation, were analyzed following CRISPR-based single-gene KO of *ERK1* and *ERK2* (Supplementary Fig. 10A). Consistent with the established role of the ERK pathway in tumor cell growth^30,31^, ERK-deficient AM38 cells exhibited slower growth compared to non-target control (NTC) AM38 cells (Supplementary Fig. 10B, C). Differential gene expression analysis comparing AM38 NTC with AM38 ERK1KO and ERK2KO revealed GO themes modulated by MAPK/ERK signaling, including antigen processing and IFN responses (Fig. 5A, B). Gene set enrichment analysis (GSEA) further demonstrated downregulation of type I and type II IFN response genes in ERK1/2KO cells, implicating ERK1/2 signaling as a critical regulator of these immune-related tumor phenotypes (Fig. 5B).

Based on these findings, we explored whether MAPK/ERK activation could mediate glioma cell responsiveness to type I IFN. Functional analysis revealed that ERK1/2 KO significantly impaired the expression of type I IFN response genes (*IRF7*, *IRF9*, and *ISG15)*^32^ following IFN-α exposure in AM38 cells (Supplementary Fig. 11A). Given our previous observation that p-ERK in GBM cells is associated with a distinct microenvironment phenotype^11^, the effect of ERK1/2 KO on cytokine secretion in response to IFN-α was examined. While AM38 secreted CCL3/4 and GM-CSF following IFN-α exposure, this was attenuated in AM38 ERK1 KO and ERK2 KO cells (Supplementary Fig. 11B).

Analysis of a large public GBM scRNA-seq dataset (GBmap)^33^ revealed differential expression of gene signatures derived from AM38 NTC and AM38 ERK1/2KO across various cell populations (Fig. 5C). The AM38 NTC signature (associated with elevated p-ERK; Supplementary Fig. 10A) was mainly expressed within tumor cells, particularly AC-like and NPC-like populations. Conversely, the AM38 ERK1/2 KO signature was predominantly enriched in the MES-like cell population, whereas IFN molecules, including IFN-α, IFN-β, and IFN-γ, were primarily expressed by T cells and myeloid cells (Fig. 5D).

We utilized a novel autologous human neocortical slice model from a *BRAF*^*V600E*^-mutated GBM patient to explore the causal relationship between MAPK/ERK signaling and these phenotypes in human GBM tissues (Supplementary Fig. 12A)^34^. *BRAF*^*V600E*^-mutant tumor cells were implanted into human cortex slices and treated with either temozolomide (TMZ) or BRAF/MEK inhibitors (BRAFi/MEKi), or non-treated controls. The optimal BRAFi/MEKi combination was determined based on maximal inhibition of tumor growth (Supplementary Fig. 12B). Spatially resolved transcriptomic and scRNA-seq analyses of the slices conducted 7 days after treatment (Supplementary Fig. 13A and Fig. 5E) revealed downregulation of IFN signatures in BRAFi/MEKi-treated samples compared to non-treatment controls (Fig. 5F, G). Consistent with our earlier in vivo findings, BRAFi/MEKi-induced suppression of MAPK activity was associated with reduced CD8^+^ T cell infiltration, suggesting that MAPK/ERK signaling in glioma cells may promote a microenvironment favorable to T cell recruitment (Supplementary Fig. 13B).

### MAPK/ERK-IFN signaling axis modulates MHC class II expression in glioma cells

Earlier analysis of scRNA-seq data from GBM specimens with high and low p-ERK levels revealed enrichment of GO signatures associated with both IFN type I and II response and antigen presentation (Fig. 4A). In concordance, bulk RNA-seq analysis of AM38 glioma cells with ERK1/2 knockout demonstrated that MAPK/ERK signaling modulates the expression of genes involved in antigen processing and presentation pathways (Fig. 5B), which are IFN-responsive genes.

**Fig. 6:**
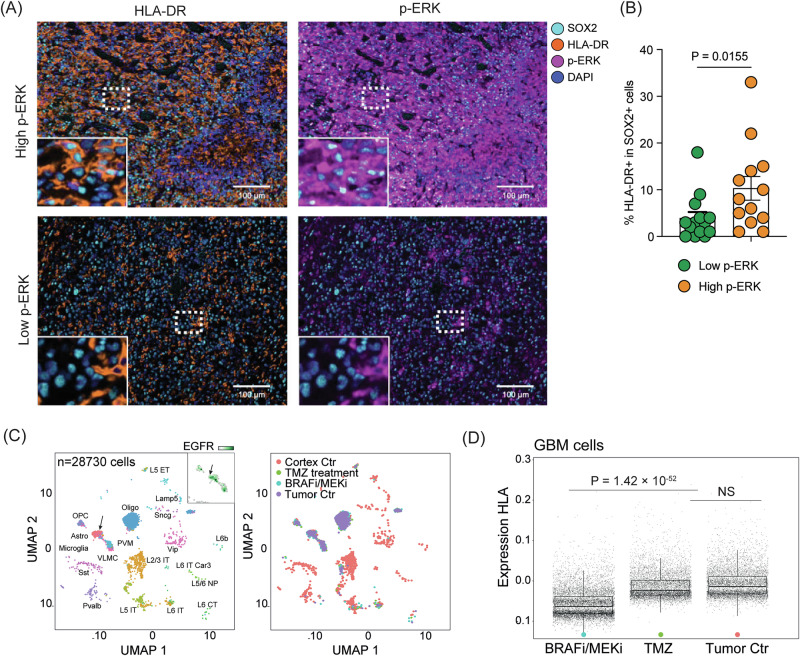
MAPK/ERK-IFN signaling axis modulates MHC class II expression in glioma cells. **A** Multiplex immunofluorescence analysis of human GBM tumors comparing high p-ERK (*n* = 13) and low p-ERK (*n* = 15) tumors. Representative images of a high p-ERK tumor (top) and a low p-ERK tumor (bottom). Scale bars, 100 µm. **B** Quantified phenotypic analysis of multiplex immunofluorescence across 28 GBM (high p-ERK, *n* = 13; low p-ERK, *n* = 15) patient samples. Bar graph showing percentage of HLA-DR^+^ cells in SOX2^+^ tumor cells, comparing p-ERK^−^ and p-ERK^+^ cells. Data are represented as mean ± s.e.m., statistical significance was determined using an unpaired one-tailed t-test. **C** Single-cell analysis from a total of 24 quantified slice-cultivated human GBM tumor (left) treated with TMZ and BRAFi/MEKi (right). **D** Differential expression of human leukocyte antigen (HLA) in slice-cultivated human GBM sample without treatment or TMZ and BRAFi/MEKi treated (*P* = 1.42 × 10^−52^). Statistical significance was determined using an unpaired Wilcoxon test performed directly in R. Every data point in these barplots represents one spatial spot from the corresponding sampling group. Source data are provided as a Source data file.

To assess the expression of MHC class II molecules in human GBM, we performed multiplex immunofluorescence staining. While HLA-DR expression was observed across diverse cell types (Supplementary Fig. 14), the density of SOX2⁺HLA-DR⁺p-ERK⁺ tumor cells was significantly higher than that of SOX2⁺HLA-DR⁺p-ERK⁻ cells (*P* = 0.0155; Fig. 6A, B), suggesting that MAPK/ERK activation enhances the capacity of tumor cells to upregulate MHC class II expression, possibly in response to IFN signaling.

We further examined the causal relationship in human *BRAF*^*V600E*^ GBM slice cultures treated with BRAFi/MEKi. scRNA-seq analysis revealed that pharmacological inhibition of the MAPK pathway resulted in marked downregulation of HLA gene expression in tumor cells when compared to either untreated or TMZ-treated samples (*P* = 1.42 × 10^−52^; Fig. 6C, D). These findings were supported by in vitro experiments in AM38 cells, in which ERK2 KO reduced the expression of MHC class II-related transcripts (Supplementary Fig. 15A) and protein levels (Supplementary Fig. 15B). A similar pattern was observed in the murine glioma line QPP7, in which KO of *Erk1* or *Erk2* led to decreased expression of MHC class II-associated genes (Supplementary Fig. 15C, D). While this finding does not necessarily imply that glioma cells can present antigens via MHC class II, it does confirm their ability to respond to IFN is modulated by MAPK signalling, as these molecules are up-regulated in response to IFN.

### MAPK signaling in tumor cells drives activation of tumor-associated microglia/macrophages in GBM

**Fig. 7:**
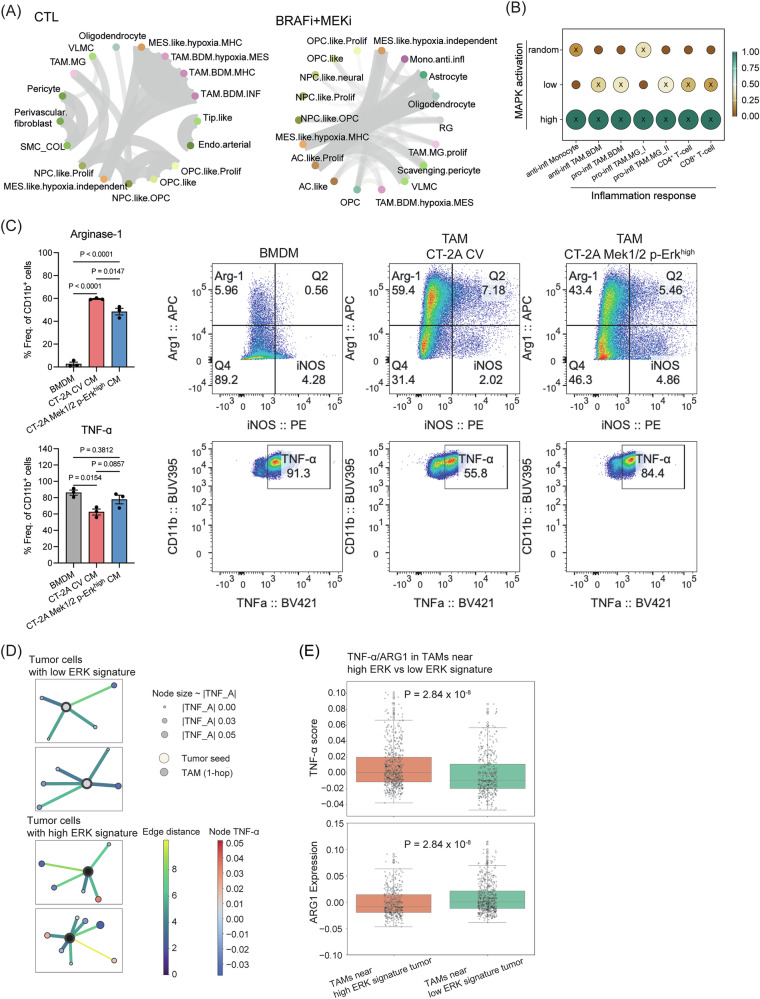
MAPK signaling in tumor cells drives activation of tumor-associated microglia in GBM. **A** Cell-to-cell communication analysis from a total of 24 quantified slice-cultivated human control GBM tumor (left) or treated with BRAFi/MEKi (right). **B** Correlation analysis between MAPK activation and inflammatory immune cell responses. **C** Phenotypic analysis of bone marrow-derived monocytes (BMDMs) cultured with glioma-conditioned media (CM) for 48 h. Flow cytometry analysis showing intracellular staining of arginase-1 (upper) and TNF-α (lower) in BMDMs exposed to complete media, CT-2A CV CM, or CT-2A Mek1/2 p-Erk^high^ CM. Bar graphs represent mean ± SEM (*n* = 3, independent experiments). Statistical significance was determined using one-way ANOVA; *P* values are shown. **D** Spatial NN analysis from MERFISH data (*n* = 6). Examples of TAMs in the NN of tumor cells and the corresponding TNF-α expression. **E** Relative expression of TNF-α (upper) or arginase-1 (lower) in TAMs in the 1-hop NN from ERK-high/low tumors (*n* = 6). Statistical significance was determined using an unpaired Wilcoxon test performed directly in R. Every data point in these barplots represents one spatial spot from the corresponding sampling group, and *P-*values are shown in the graphs. Source data are provided as a Source data file.

Building on previous observations that high p-ERK gliomas are associated with a distinct microglia phenotype^11^, the slice culture model of *BRAF*^*V600E*^ GBM was used to investigate the contribution of MAPK/ERK signaling to GBM cell-microenvironment interactions. Graph-based cell proximity and communication analysis demonstrated that BRAFi/MEKi reduced the communication between tumor cells and microglia/macrophages (Fig. 7A). Additionally, BRAFi/MEKi treatment modulated the microglial and TAM interaction; MAPK-activated tumor cells exhibit increased interactions with inflammatory microglia and a higher degree of T cell engagement (Fig. 7B). These findings demonstrate that MAPK/ERK signaling in GBM cells plays a crucial role in modulating microglia and TAM phenotypes and their interactions with tumor cells.

The pro-inflammatory TAM phenotype associated with MAPK signaling in human GBM was recapitulated in vivo in murine gliomas. Intracranial implantation of GL261 Mek1/2 p-Erk^high^ cells, which are more susceptible to CD8^+^ T cells, aPD-1, and FcE-aCTLA-4, and exhibit increased CD8^+^ T cell infiltration (Figs. 1–3), induced a shift in TAM polarization. This shift was reflected by reduced arginase-1 and PD-L1 expression, and elevated levels of CD68, CD86, and CD80 receptors (Supplementary Fig. 16). To further validate that MAPK signaling in tumor cells can modulate TAM, we conducted an in vitro phenotype analysis of bone marrow-derived monocytes (BMDMs) from healthy mice cultured with glioma cell-conditioned media (CM). TAMs derived from BMDMs cultured with CT-2A CV CM exhibited higher expression of arginase-1 and decreased level of TNF-α, in line with an immunosuppressive TAM-like phenotype. In contrast, BMDMs exposed to CT-2A Mek1/2 p-Erk^high^ CM showed reduced expression of arginase-1 and up-regulation of TNF-α (Fig. 7C). CD11b^+^ myeloid cells were gated from live single cells and further analyzed for arginase-1, iNOS, and TNF-α expression to assess their functional phenotype (Supplementary Fig. 4B). An analysis of human GBM MERFISH data (Fig. 7D) showed that TAMs surrounding high ERK signature regions exhibited increased expression of TNF-α and reduced expression of arginase-1, consistent with a more pro-inflammatory and immunostimulatory phenotype (Fig. 7E). These findings parallel our in vitro observations from the CT-2A model and reinforce the notion that MAPK/ERK signaling in glioma cells can reprogram surrounding TAMs toward a more immunogenic state.

## Discussion

Our findings demonstrate the causal contribution of glioma-intrinsic MAPK/ERK signaling in modulating anti-tumoral immunity and responsiveness to immunotherapy. We provide mechanistic insight into the previously observed association between MAPK-activating mutations, elevated p-ERK levels, and improved survival in GBM patients treated with aPD-1 and aCTLA-4 immune checkpoint blockade therapy^11^^,^^12^^,^^18^. While MAPK signaling is known for its oncogenic and proliferative effects in cancer,^30^ our results reveal its crucial role in shaping the tumor-immune microenvironment, supporting the paradigm that molecular heterogeneity in GBM tumors is linked to the variability in the immune microenvironment, TAM phenotypes, and ultimately, susceptibility to immunotherapy^9,35,36^.

We provide substantive evidence that MAPK activation in GBM cells enhances CD8^+^ T cell-mediated immune responses. Our CRISPR/Cas9 screen implicated the RAF-MEK-ERK branch of the MAPK pathway in glioma susceptibility to T cell recognition. This finding is corroborated by the differential responses to aPD-1 therapy in wild-type versus *Cd8* KO mice. The distinct phenotype of CD8^+^ T cells infiltrating the brains of long-term survivor mice with high p-ERK tumors treated with aPD-1 further supports the role of these cells in the observed anti-tumor immune response. Moreover, p-ERK promoted CD8^+^ T cell infiltration in mouse gliomas, with similar observations in human GBM specimens. Consistent with this, clinical observations showed robust brain/lesional T cell infiltration in patients with elevated p-ERK who responded to aPD-1 therapy^11^. Additionally, in transgenic murine glioma models, we demonstrated that gliomagenesis in *Cd8* KO hosts, which lack CD8^+^ T cells, leads to tumors with elevated p-ERK that are also more immunogenic^37^.

The mechanism by which MAPK/ERK signaling enhances T cell recruitment into the GBM tissue and response to immunotherapy appears to involve modulation of tumor cell immunogenicity and its relationship with the tumor-infiltrating microenvironment, particularly microglia. Our findings highlight the role of ERK phosphorylation in modulating both type I and type II IFN responses in glioma cells, consistent with previous literature implicating MAPK/ERK signaling in the IFN response in other settings^31,38^. This regulation occurs through the activation of signaling via the STAT1 pathway, which drives the expression of genes involved in immune responses^39^. The mechanistic link between p-ERK and IFN responses in GBM is supported by our results, which indicate that inhibition of ERK signaling impairs the induction of IFN response genes and dampens the overall immune response against tumors.

p-ERK was found to promote the expression of MHC molecules in GBM cells, likely related to the IFN response driven by MAPK/ERK signaling. This is consistent with MHC molecules being a downstream effect of IFN signaling^40–42^. We do not know whether the expression of these MHC class II molecules translates into increased antigen presentation, the increase in MHC molecule expression, the pro-inflammatory polarization of microglia, and the enhanced CD8⁺ T cell infiltration observed in MAPK/ERK-activated tumors are all mechanisms known to contribute to effective tumor clearance^11^. The immune-modulating mechanisms driven by MAPK activation in GBM cells translate into robust anti-tumoral immune responses beyond aPD-1 blockade, as evidenced by enhanced susceptibility to aCTLA-4 therapy. This suggests that MAPK/ERK signaling may serve as a predictor of response to various forms of GBM immunotherapy and novel treatments that rely on T cell activity, such as vaccines, CAR-T cells, and BiTEs^43–45^. Importantly, our findings should not be interpreted as proposing the combination of MAPK/ERK pathway activation with immune checkpoint blockade. Rather, our study identifies MAPK/ERK activity as a biologically relevant determinant and potential biomarker of immunotherapy responsiveness in glioblastoma.

While MAPK/ERK activation contributes significantly to anti-tumoral immune responses, it appears to be necessary but not sufficient to elicit robust tumor rejection following immunotherapy. Indeed, not all GBM patients with elevated p-ERK benefit from aPD-1 therapy, whereas no patient with low p-ERK exhibited long survival after ICB^11^. Other factors, including the presence of tumor-specific antigens, bone marrow-derived immunosuppressive myeloid cells, T cell sequestration in the bone marrow, lymphopenia, and the use of steroids, may also play critical roles and influence tumor susceptibility to ICB^7,46,47^. Moreover, several additional tumor-intrinsic features have been reported to modulate anti-tumoral immunity^12,19^.

While our work provides evidence for the role of MAPK/ERK signaling in modulating GBM immunogenicity and immunotherapy response, several limitations should be considered. One of them is the use of *Cd8* KO mice in our CRISPR screen. We selected this system to achieve a clean and specific genetic depletion of cytotoxic CD8^+^ T cells, thereby enabling a focused evaluation of their role in mediating MAPK/ERK pathway-related immune responses. Compensatory mechanisms involving CD4^+^ or unconventional T cells may exist in *Cd8* KO mice^48^. We do not think this had a major influence on our results, as in a previous publication, we demonstrated that CD8^+^ T cells are the primary population depleted in this model, no robust compensatory expansion of other immune subsets^19^. Our experimental models, though informative, may not fully capture the complexity of human GBM. Likewise, while our *BRAF*^*V600E*^ mutant GBM slice culture model offers a unique platform to study tumor-microenvironment interactions, it lacks the systemic immune components and may not fully represent the in vivo tumor immune microenvironment. Despite these limitations, our multi-modal approach, combining in vitro studies, in vivo models, ex vivo human analyses, and large-scale “-omic” approaches, provides a framework for understanding the role of MAPK/ERK signaling in GBM immunobiology that is consistent across platforms, models, and species. Future studies addressing these limitations will be crucial for translating these findings into clinical applications and potentially paving the way for more effective, personalized immunotherapies in GBM.

Our work underscores the critical role of the RAF-MEK-ERK pathway in regulating immune responses in GBM, offering new insights into the molecular mechanisms that drive immunotherapy responsiveness. By linking MAPK/ERK signaling to T cell-mediated tumor recognition and response, these findings offer insights into the heterogeneity of GBM and, more importantly, the potential of MAPK/ERK activation as a predictive biomarker and therapeutic target. These findings have implications for personalized immunotherapy in GBM. Indeed, these results highlight the need for molecularly guided approaches to address the heterogeneity of GBM treatment responses, paving the way for more effective, tailored strategies in this challenging malignancy, where efficacy is designed for subsets of patients with molecularly defined tumors.

## Methods

### Orthotopic mouse glioma model

All mouse work performed in this study was approved by the Northwestern Institutional Animal Care and Use Committee (IACUC) under protocol number IS00014080. C57BL/6 mice were purchased from Charles River Laboratories and housed in a pathogen-free animal facility at Northwestern University Center for Comparative Medicine. Mice were maintained under controlled environmental conditions with a 12-h light/12-h dark cycle, ambient temperature of 20–24 °C, and relative humidity of 30–70%, with ad libitum access to food and water. For tumor cell implantation, 6–8-week-old mice were anesthetized with ketamine (100 mg/kg) and xylazine (10 mg/kg), and 100,000 glioma cells in 2.5 µl DPBS were injected intracranially at a specific brain coordinate after disinfecting and making a small cranial opening at 3 mm lateral and 2 mm caudal relative to the bregma using a stereotaxic device (Harvard Apparatus). Post-surgery, animals were treated with 10 mg/kg of IC or ICB antibodies 4 times every 3–4 days started on day 7 post tumor implantation and monitored until endpoints defined by the IACUC were met, including significant body weight loss, severe or progressive neurological symptoms, impaired mobility, lethargy, hunched posture, poor grooming, dehydration, inability to access food or water, or other signs of distress or morbidity requiring euthanasia.

### Cell culture

The mouse glioma cell line GL261 was obtained from the National Institutes of Health, and CT-2A was purchased from Millipore Sigma (SCC194). Both cell lines were cultured in Dulbecco’s modified Eagle’s medium supplemented with 10% fetal bovine serum and 1% penicillin/streptomycin. QPP7 cells^21,49^, kindly provided by Dr. Amy B. Heimberger from Northwestern University, were maintained in Dulbecco’s modified Eagle’s medium/F-12 (Gibco) supplemented with B-27 (Gibco), recombinant epidermal growth factor (EGF, 20 ng/ml, Peprotech), and recombinant basic fibroblast growth factor (bFGF, 20 ng/ml, Peprotech). All the cells were maintained in 5% CO_2_ incubators at 37 °C and tested for mycoplasma and confirmed negative before intracranial injection. Patient-derived GBM cells after a single cell suspension procedure were seeded in T-75 culture flasks and preserved in the cell culture incubator at 37 °C with 5% CO_2_ for recovery and following experiments. GM was used as a cell culture medium for patient-derived GBM cells in this study.

### Kinome-wide CRISPR Cas9 screening

In vivo kinome CRISPR screens were performed using the Brie mouse CRISPR knockout pooled library (Addgene). Lentivirus was produced in HEK293T cells, and GL261 murine glioma cells were transduced with the kinome library at a multiplicity of infection (MOI) < 0.3, followed by 2 µg/mL puromycin selection for 7 days. For the *Cd8* KO screen, 200,000 cells per mouse, GL261 cells were implanted intracranially into either wild-type C57BL/6 mice (*n* = 11) or *Cd8* KO mice (*n* = 11). For aPD-1 screen, similarly transduced GL261 cells were implanted into C57BL/6 wild-type mice treated with anti-PD-1 antibody (200 µg intraperitoneally twice a week for 2 weeks, *n* = 12) or IC (*n* = 12). Tumors were harvested as mice reached humane endpoints, and genomic DNA was extracted for further analysis. Both *Cd8* KO and anti-PD-1 kinome-wide CRISPR screens were analyzed using the Model-based Analysis of Genome-wide CRISPR-Cas9 Knockout (MAGeCK) computational tool, with detailed methodology, procedures, and controls described in a previous publication^19,50^. Briefly, the MAGeCK count command was used to map FASTq files to the sgRNAs in the Brie library. Counts were normalized to internal non-target sgRNA controls, and gene essentiality scores (beta-scores) was determined for each group relative to day 0 baseline controls using the MAGeCK MLE method.^51^ Kyoto Encyclopedia of Genes and Genomes (KEGG) Pathway enrichment analysis was performed on significant genes (FDR < 0.05) in R (v4.2.3)^52^ using clusterProfiler (v4.12.0)^53^ package, and plots were generated using enrichPlot (v1.24.0)^54^.

### Immunophenotype analysis

Tumor-bearing brains were processed for immune profiling on 20 days post-intracranial tumor injection after 2 treatments. Following preparation of Percoll gradient-enriched single-cell suspension using a 70 µm cell strainer, cells underwent Fc blocking (TrueStain FcX, Biolegend) for 10 min for further staining process. Cells then underwent surface staining with primary antibodies and live/dead staining with Fixable Viability Dye eFluor 780 (eBioscience). After fixation and permeabilization (Foxp3/Transcription Factor Staining Buffer Set, eBioscience), intracellular staining was performed. Antibodies used in this study were listed in Supplementary Table 1. Flow cytometry data were acquired by the BD Symphony and analyzed by FlowJo 10.8.1 (BD).

### Multiplex immunofluorescence staining

Multiplex immunofluorescence was performed as described^18^. The sections undergo deparaffinization with BOND dewax solution and epitope retrieval process using a heat-induced method with BOND epitope retrieval solution or pH9 EDTA buffer. DAB staining for immunohistochemistry was performed to optimize antibody concentrations. The antibodies used in this analysis and the dilution factor were depicted in Supplementary Table 2. Multiple cycles of heat-induced epitope retrieval, protein blocking, epitope labeling, and signal amplification were performed for multiplex staining, then the slides were counterstained using spectral DAPI, and finally mounted using Prolong Diamond Antifade Mountant (Thermo). First, whole slide images were acquired after auto-adjusting focus and signal intensity. Then, MSI was acquired in the tumor regions delineated by a board-certified neuropathologist at ×20 of the original magnification. For analysis of MSI, we created a spectral library for all Opal dyes to subject acquired multispectral images to spectral unmixing, enabling the identification and separation of weakly expressing and overlapping signals from background and subsequent visualization of the signal of each marker in the inForm Tissue Finder software (inForm 2.6, Akoya Biosciences). inForm’s adaptive cell segmentation feature was used to identify the nucleus of analyzed cells and determine the nuclear and cytoplasmic compartments on each cell. A machine-learning algorithm within inForm was also used to automatically assign cells to a specific phenotype (GFAP^+^, TMEM119^+^, CD163^+^, CD16^+^, CD11c^+^, and HLA-DR^+^). Batch analysis was used to analyze all tumor samples under the same segmentation and phenotype settings. The processing and analysis of images from all tumor samples were exported to cell segmentation tables. Exported files from inForm were processed in R (R packages Phenoptr and PhenoptrReports) to merge and create consolidated single files for each tumor sample. Consolidated files had cell phenotypes as outputs that we employed for further quantification and spatial analyses using the Phenoptr R addin.

### RNA extraction and quantitative RT-PCR

Total cellular RNA was isolated using TRIzol reagent (Invitrogen) following standard phenol–chloroform extraction procedures. For reverse transcription, 1 μg of RNA was converted into cDNA with the iScript cDNA synthesis kit (Bio-Rad) according to the protocol from the supplier. Quantitative real-time PCR assays were carried out using iTaq 2X SYBR Green mix on a CFX Real-Time PCR platform (Bio-Rad). Primer sequences used for amplification are provided in Supplementary Table 3.

### Western blot analysis

Cells were harvested in lysis buffer (Cell Signaling Technology) supplemented with protease and phosphatase inhibitor cocktails (Thermo Fisher Scientific). Protein samples were denatured at 95 °C for 10 min prior to electrophoresis. Twenty micrograms of protein were separated by SDS-PAGE and subsequently transferred onto PVDF membranes (Millipore). Membranes were incubated with the indicated primary antibodies, followed by appropriate secondary antibodies, and signal detection was performed using enhanced chemiluminescence reagents (Thermo Fisher Scientific). A complete list of antibodies used in this study is provided in Supplementary Table 4.

### Patient sample

The ethics approval was issued by the local committee of the University of Freiburg for data evaluation, imaging procedures, and experimental design (Freiburg: protocol 100020/09 and 472/15_160880). All methods were carried out in accordance with the approved guidelines, with written informed consent obtained from all subjects. The patient provided preoperative (in the Department of Neurosurgery of the Medical Center-University of Freiburg, Freiburg im Breisgau, Germany) informed consent to take part in the study. Therapeutically resected access cortical tissue and glioblastoma tumor tissue were collected and transported to the laboratory immediately after surgical resection.

### Human organotypic slice culture system

Human organotypic slices were prepared and cultured as we previously described^55^. Therapeutically resected access cortical tissue was immediately collected and immersed in the Preparation Medium saturated with carbogen (95% O_2_ and 5% CO_2_). Damaged tissue was dissected away from the primary tissue block. The primary tissue block was further dissected into smaller tissue blocks (6 × 6 mm^2^ dimension) for tissue sectioning. Sectioning was carried out using a Leica VT1200 semi-automatic vibratome (14912000001, Leica, Germany). Three hundred micrometers evenly thick cortical sections were collected in growth medium (GM) using a capillary glass pipette and settled on ice for 10 min for recovery. Recovered slices were plated on MilliporeTM inserts (PIPH03050, Merck KGaA, Germany) in 6-well plates (657160, Greiner BIO-ONE, Germany) supplied with GM and further cultured in the incubator at 37 °C with 5% CO_2_ for the following experiments. Three sections per culture well are recommended. The first medium refreshing was carried out 2 h post tissue plating, followed by medium refreshing every 48 h on a regular basis throughout the culture period.

### Autologous GBM cell suspension

Patient-derived GBM cell suspension was carried out, as we previously described using a Neural Tissue Dissociation Kit (T) (130-093-231, Miltenyi Biotec, Germany)^55^. Primary GBM tissue obtained from the surgery was immediately processed and dissociated with the Neural Tissue Dissociation Kit (T) in C-tubes (130-093-237, Miltenyi Biotec, Germany). Briefly, tissue was dissected into small pieces, incubated in enzyme 1 and enzyme 2 for 10 min with 2 min of tissue homogenizing in between each incubation. A filtering step was followed to remove all visible tissue pieces with pia matter. Single cells in the filtered supernatant were further processed to remove the myelin population along with red blood cells and non-viable cells. Eventually, viable single patient-derived GBM cells in the final supernatant were seeded into T-75 cell culture flasks for cell culture or directly snap-frozen in cryopreservation medium at −80 °C.

### GBM cell labeling and detection assay

Cell TraceTM CFSE dye (C34570, ThermoFisher, USA) was used in this study to label the autologous GBM cells. Tumor cells from cell culture were washed with PBS and detached with Accutase for 5 min. CFSE dye was prepared with the manufacturer’s protocol. In a ratio of 1 µL dye per 2 × 10^6^ cells, the CFSE dye was used to label the tumor cells. Tumor cells were further observed under EVOS M7000 microscope (AMF7000, Thermofisher, USA) coupled with its onstage incubator system (AMC1000, ThermoFisher, USA) to confirm the success of fluorescent labeling for tumor cells.

### Personalized GBM model

Personalized GBM model system was generated based on the autograft of GBM cells into the patient’s cortical sections. The CFSE-labeled tumor cells were resuspended and inoculated into the patient’s cortical sections using a Hamilton micro syringe (80330, Hamilton, Switzerland) in the concentration of 20,000 cells per 1 µL.

### Tumor growth monitoring

Fluorescent images of sections after inoculation were acquired using EVOS M7000 microscope coupled with its onstage incubator system for monitoring tumor growth on a time-resolved manner of every 24 h till the end point of the culture experiment.

### Fluorescence-associated cell sorting

Fluorescence-associated cell sorting (FACS) was performed to screen targeted cell/nucleus populations. In brief, tissue was homogenized, and nuclei were isolated using Nuclei EZ Prep (NUC101-1KT, Merck KGaA, Germany) lysis buffer. Nuclei were labeled using DAPI (32670#5MG, Merck KGaA, Germany). Myelin and debris were removed by a sucrose gradient centrifugation step. 35,000 DAPI^+^ events per condition were collected from FACS sorter using 1.5 mL eppis coated with 100 µL 2% BSA.

### Single nucleus RNA-sequencing

Single-nucleus RNA-sequencing, a droplet-based approach, was performed according to the Chromium Next GEM Single Cell 3′ v3.1 protocol from 10X Genomics. Nuclei collected from FACS sorting were added to a prepared Master Mix and loaded in the Chromium Controller for RNA recovery and generating GEMs. After reverse transcription and cDNA amplification, the enriched cDNA was fragmented, size-selected using SPRIselect (B23318, Beckman Coulter, USA), indexed using i7 index, and SI primer was added. The average length of final libraries was quantified using a 5200 Fragment Analyzer (M5310AA, Agilent, USA) with its HS NGS Fragment kit (DNF-474, Agilent, USA), and the concentration was determined using QubitTM 4 Fluorometer (Q33238, ThermoFisher, USA) with its 1× dsDNA HS kit (Q33231, ThermoFisher, USA). The diluted, pooled, and denatured final library was loaded in Illumina NextSeq 550 Sequencing System. NextSeq High Output kit v2.5 (20024906, Illumina, USA) was used in this study. Sequencing cycles for read1 – i7 – i5 – read2 were: 28 – 8 – 0 – 56.

### Spatially resolved transcriptomics

Ten-micrometer-thick sections from fresh frozen tissue were mounted on a specially designed spatially barcoded Visium Gene Expression Slide (1000188, 10X Genomics, Netherlands). The mounted slide was fixed in 100% methanol following with H&E staining. EVOS M7000 microscope, coupled with a 20× magnification lens, was used to acquire bright field images. Post-imaging processing was performed using the FIJI ImageJ software. A permeabilization with pre-optimized incubation time was further performed to maximize capture oligo binding with the sample mRNA. After reverse transcription, the second-strand cDNA was then cleaved off by denaturation. Following a qPCR quantification using KAPA SYBR FAST qPCR Master Mix (KK4600, Roche, USA), cDNA was amplified and fragmented, and further size selected using SPRIselect. Similar to single-nucleus RNA-sequencing library preparation, cDNA library quality was quantified using a 5200 Fragment Analyzer, further indexed, amplified, and double-sided size selected, followed by an average base pair length quantification using a 5200 Fragment Analyzer and a concentration quantification with QubitTM 4 Fluorometer. The final sequencing library was generated after a dilution, normalization, and denaturation step. Illumina NextSeq 550 Sequencing System, coupled with its NextSeq High Output kit v2.5 (20024904, Illumina, USA), was used in this study. Sequencing cycles for read1 – i7 – i5 – read2 were set up as 28 – 10 – 10 – 102. To define the p-ERK signal, we used the MSIGDB database with the pathways “BP.GO_POSITIVE_REGULATION_OF_ERK1_AND_ERK2_CASCADE” with the SPATA2 functions: gene sets via SPATA2::getSignature(). The SPATA2 framework was recently published,^56–58^, including the various options for gene set enrichment analysis. To characterize not only the overall enrichment of the p-ERK pathway in the samples, we performed spatial autocorrelation to determine if the ERK pathway is enriched in local niches or generally upregulated across the sample. The enrichment analysis of IFN was also performed by gene-set enrichment analysis using the IFN signatures: “BP.GO_RESPONSE_TO_INTERFERON_ALPHA;” “BP.GO_RESPONSE_TO_INTERFERON_BETA;” “BP.GO_RESPONSE_TO_INTERFERON_GAMMA”.

### MERFISH of GBM samples

For the MERFISH experiments, we designed a 500-gene panel consisting of genes that are discriminative of the different cellular populations within GBM. The panel consists of GBM cell state markers and over 15 cell types and subtype markers. We initially selected 600 genes and excluded 100 genes with low abundance and insufficient target regions using the MERSCOPE Gene Panel Design Portal (https://portal.vizgen.com). To avoid optical crowding for MERSCOPE imaging, SPP1, CHI3L1, LGALS1, and GFAP were moved to auxiliary channels and imaged as RNAs. 50 blank barcodes were added to the gene panel as a control for unspecific binding of probes. FFPE (formalin-fixed paraffin-embedded) tissue preparation for MERFISH was performed following the MERSCOPE FFPE Sample Preparation guide (https://vizgen.com/-resources/merscope-formalin-fixed-paraffin-embedded-tissue-sample-preparation-user-guide/). Tissue sections (5μm thick) were cut from FFPE blocks using a microtome and mounted onto MERSCOPE standard FFPE slides (Vizgen 10500110). The sections were deparaffinized using the deparaffinization buffer (Vizgen 20300112) and washed with graded ethanol concentration (100% ethanol for 2 min, 90% ethanol for 2 min, and 70% ethanol for 2 min) to rehydrate the tissue. The rehydrated tissue sections were incubated with the Decrosslinking Buffer (Vizgen 20300115), followed by anchoring pretreatment and RNA anchoring. Following RNA anchoring, the tissue sections were embedded in hydrogel using the Gel Embedding solution consisting of Gel Embedding Premix (Vizgen 20300118), ammonium persulfate (Sigma, 09913-100 G), and TEMED (N,N,N′,N′-tetramethylethylenediamine) (Merck, 1.10732.0100). A coated cover slip was placed on the gel solution to aid in gel solidification. The samples were then cleared using a solution containing Proteinase K (NEB, P8107S) and Clearing Premix (Vizgen 20300114), incubated at 47 °C for 24 h and then at 37 °C in a humidified incubator for 2–3 days until the tissue sections became transparent. The samples were placed in a photobleacher for 5 h to quench autofluorescence. The samples were then washed in sample prep wash buffer (Vizgen 20300001) and formamide wash buffer (Vizgen 20300002) and hybridized with our custom MERSCOPE Gene Panel Mix of 500 genes (Vizgen 10400003) at 37 °C for 36–48 h. After hybridization, tissue sections were washed with formamide wash buffer. DAPI and Poly T Reagent (Vizgen 20300021) was then added to the tissue sections for 15 min, washed, and then the MERSCOPE 500 gene imaging kit was used for imaging (Vizgen 10500110).

### MERFISH analysis

Spatial transcriptomics data were processed using the sopa package. Raw spatial transcriptomics data were loaded into a SpatialData object using sopa.io.merscope. Cellpose segmentation was performed using the sopa.segmentation.cellpose function to delineate individual cells. Image patches of 6000 pixels with a 150-pixel overlap were first created using sopa.make_image_patches function. The Cellpose model was applied using the DAPI channel as the nuclear marker, with a specified cell diameter of 60 pixels, as recommended for MERFISH data. The initial cell segmentation was complemented with transcript-level segmentation using Baysor34. Transcript patches were created using, utilizing cellpose boundaries as prior shape information. Baysor segmentation was executed with a minimum cell area threshold of 10 µm². Cells with an area below this threshold were removed from the dataset. Transcripts with fewer than 10 molecules per gene and cells with fewer than 20 detected molecules were filtered out to enhance segmentation accuracy. Single-cell feature matrices were obtained using the sopa.aggregate function. An AnnData object with transcript counts per segmented cell and computed average channel intensities for each region were created. Cell type annotation was performed using Tangram, mapping spatial transcriptomics data to a modified GBmap scRNA-seq reference dataset. For data analysis, we leveraged our SPATA2 framework. First, we initiated a SPATA object from the anndata inputs using the cell coordinates, gene-by-cell count matrix. For normalization, scVI variational autoencoder36 was trained (sample-wise) with a *n* = 50 dimensions for the latent space and 200 epochs per sample (scvi.model.SCVI). The reconstructed expression matrix (model.get_normalized_expression) was implemented along with log-scaled normalization. Cell shapes were implemented from cell pose (explained in the sopa pipeline) into the SPATA (@spatial) slot. To separate various samples within the same ROI, we manually segmented the cells and split the SPATAobject (splitbybarcode) into sample-individual objects.

### Spatial neighborhood analysis

For the neighborhood analysis of T cells, we started by embedding the samples into a spatial graph leveraging triangulation (Delaunay triangulation) implemented in the “deldir” package. We computed the Euclidean distance between all cells and initiated a graph object (igraph) with cells as nodes and distance as edge features. For all ERK^+^ cells, we extracted the 1-hop subgraph and weighted the importance of the neighborhood by distance measurement $({\varpi }_{i}):{\varpi }_{i}=\frac{\min (d)}{{d}_{i}}$. We summarized the cell-cell importance within each treatment condition and epigenetic subgroups, resulting in a cell-to-neighborhood adjacent matrix, which was normalized using a power function and graph construction.

### Statistical analyses

Statistical analyses were performed using Prism Software 9.4.1 (GraphPad) and R Software 4.4.1 (R Studio). Unpaired Student’s *t* test was used to compare statistical differences between the two groups. For Kaplan–Meier survival curves, the log-rank (Mantel–Cox) test was adapted to determine the significance between groups. Statistical significances were presented in *P*-value, or *P* < 0.05 was considered significant, *P < 0.05, **P < 0.01, ***P < 0.001, and ****P < 0.0001.

### Reporting summary

Further information on research design is available in the Nature Portfolio Reporting Summary linked to this article.

## Supplementary information

Supplementary Information

Reporting Summary

Transparent Peer Review File

## Source data

Source Data

## Data Availability

The dataset from the CRISPR/Cas9 screen in *Cd8* KO mice is published and available under BioProject ID PRJNA822842^19^. The CRISPR/Cas9 screen data using PD-1 blockade is available under BioProject ID PRJNA1164273. Single-cell data from the patient dataset, single-cell data from the neocortical slice model, and Visium data from the slice experiment have been deposited in public repositories. The processed BRAF patient-derived Visium and scRNA-seq datasets generated in this study have been deposited in the Gene Expression Omnibus under accession number GSE331374. Raw sequencing files from human/patient-derived samples were not deposited in an unrestricted public repository due to privacy and ethical restrictions related to potentially identifiable human genetic information. Access requests should be directed to the corresponding author and will be reviewed subject to institutional approval and/or a data use agreement. The remaining patient Visium datasets were previously published^58^ and are publicly available through Dryad at 10.5061/dryad.h70rxwdmj. Publicly available GBMmap dataset can be found via cellxgene (https://cellxgene.cziscience.com/collections/999f2a15-3d7e-440b-96ae-2c806799c08c)^33^. The remaining data are available within the article, Supplementary Information, or Source data file. Source data are provided with this paper.
