# Supplementary material for: Glioma-intrinsic MAPK/ERK signaling promotes immunotherapy efficacy through T cell infiltration and interferon responses
Source: Nat Commun. 2026 Jun 25;17:7968. doi: 10.1038/s41467-026-74124-7 (PMC13448493; doi:10.1038/s41467-026-74124-7)
Supplement: Supplementary file 1 [file 41467_2026_74124_MOESM1_ESM.pdf]

Supplementary table 1. Antibodies used for flow cytometry analysis.

Marker	Antibody	Dilution
CD8	CD8a-BV605 (Biolegend 100744)	1:200
CD4	CD4-PE (Biolegend 100408)	1:200
NK1.1	NK1.1-BV785 (Biolegend 108749)	1:200
CD19	CD19-PEFire640 (Biolegend 115574)	1:200
CD45	CD45-BV510 (Biolegend 103138)	1:200
CD11b	CD11b-BUV395 (BD 563553)	1:200
Foxp3	FOXP3-AF488 (Biolegend 320012)	1:100
CD44	CD44-BV711 (Biolegend 103057)	1:200
CD62L	CD62L-PE-Cy7 (Biolegend 104418)	1:200
PD-1	PD1-PEFire640 (Biolegend 135258)	1:200
Arg-1	Arg1-APC (Invitrogen 17-3697-82)	1:100
iNOS	iNOS-PE (Invitrogen 12-5920-82)	1:100
TNF-a	TNFa-BV421 (Biolegend 606318)	1:100
CD80	CD80-BV605 (Biolegend (104729)	1:200
CD68	CD68-AF594 (Biolegend 137020)	1:200
CD86	CD86-PE-Cy7 (Biolegend 105013)	1:200
PD-L1	PDL1-BV650 (Biolegend 124336)	1:200

Supplementary table 2. Antibodies used for multiplex immunofluorescence analysis.

p-ERK: dilution 1:1600, CellSignaling
HLA-DR: dilution 1:1000, Abcam
SOX2: dilution 1:500, Abcam
GFAP: dilution 1:300, Abcam
TMEM119: dilution 1:200, sigma
CD163: dilution 1:600, Abcam
CD16: dilution 1:100, Abcam
CD11c: dilution 1:300, Abcam

Supplementary table 3. Primer list.

qRT-PCR	Forward Primer	Reverse Primer
Ciita	ACCTTCGTCAGACTGGCGTTGA	GCCATTGTATCACTCAAGGAGGC
H2Eb1	TCCAGTGGCTTTGGTCAGAGAC	CAGGTTCTCCTCCAGGTTGTAG
H2Eb2	TGCCTCAGTAGACAGGTGCAGA	AGAGCAGACCAGGAGGTTATGG
H2Ab1	GTGTGCAGACACAACTACGAGG	CTGTCACTGAGCAGACCAGAGT
H2Oa	ACGACGCTTCTGGACAGTTCAC	ATCAGCCCACTCTGAAAGTCCG
H2Ob	TGCTGACCAGTGGAACAAACGC	CCTCTCTGGATACACTGTCACC
H2DMa	GAGATTGACCGCTACACGGCAA	GAAGACAATGCCCATGATGGTGC
H2DMb1	AGAGCCTTCTCCAGCGTTTGCA	TGTGGTTTGGGCTACTCGGACA
H2DMb2	ACCTTTCTGGGATGTGCTGACC	GTGATGGTCACATCCGCTGGAT
mGapdh	CATCACTGCCACCCAGAAGACTG	ATGCCAGTGAGCTTCCCGTTCAG
IRF7	CTTCGTGATGCTGCGGGATA	TTCTCGCCAGCACAGCTC
IRF9	CCTCCAGCCAAGACAATGGA	TTACCTGGAACTTCGGTGGG
ISG15	GTTCATGAATCTGCGCCTGC	ATTTCCGGCCCTTGATCCTG
HLA-DRA	AGCTGTGGACAAAGCCAACCTG	CTCTCAGTTCCACAGGGCTGTT
HLA-DRB1	GAGCAAGATGCTGAGTGGAGTC	CTGTTGGCTGAAGTCCAGAGTG
HLA-DRB5	GAACAGCCAGAAGGACTTCCTG	GCAGGATACACAGTCACCTTAGG
HLA-DRB6	TTGGAGCAGGCTAAGTGTGAG	GTTCCGTAACTGCCTGGAACT
HLA-DPA1	ATCCAGCGTTCCAACCACACTC	CGTTGAGCACTGGTGGGAAGAA
HLA-DPB1	GTGCAGACACAACTACGAGCTG	CCTGGGTAGAAATCCGTCACGT
HLA-DQA1	GCATTGTGGTGGGCACTGTCTT	TCTTCTGCTCCTGTAGATGGCG
HLA-DOA	ATCGCCGCAATCAAAGCCCATC	TGCAGATGAGGATGTTGGGCTG
HLA-DMA	GTTCTGCGAGTGGATGATCCAG	TGTTGGGCTTGCCAAACTCCAG
HLA-DMB	CACTTACACCTGTGTGGTAGAGC	GCAGACACAGAAACCTTCAGGG
hGAPDH	GTCTCCTCTGACTTCAACAGCG	ACCACCCTGTTGCTGTAGCCAA

Supplementary table 4. Antibodies used for western blot analysis.

Western Blot Antibodies	Company	Cat. No.	Dilution
p-MEK	Cell Signaling Technology	9154S	1:1000
MEK	Cell Signaling Technology	9126S	1:1000
p-ERK1/2	Cell Signaling Technology	4370S	1:1000
ERK1/2	Cell Signaling Technology	4695S	1:1000
GAPDH	Cell Signaling Technology	5174S	1:1000
ERK1	Abcam	ab32537	1:500
ERK2	Cell Signaling Technology	9108S	1:1000
Anti-rabbit IgG, HRP-linked Antibody	Cell Signaling Technology	7074S	1:5000
Anti-mouse IgG, HRP-linked Antibody	Cell Signaling Technology	7076S	1:5000

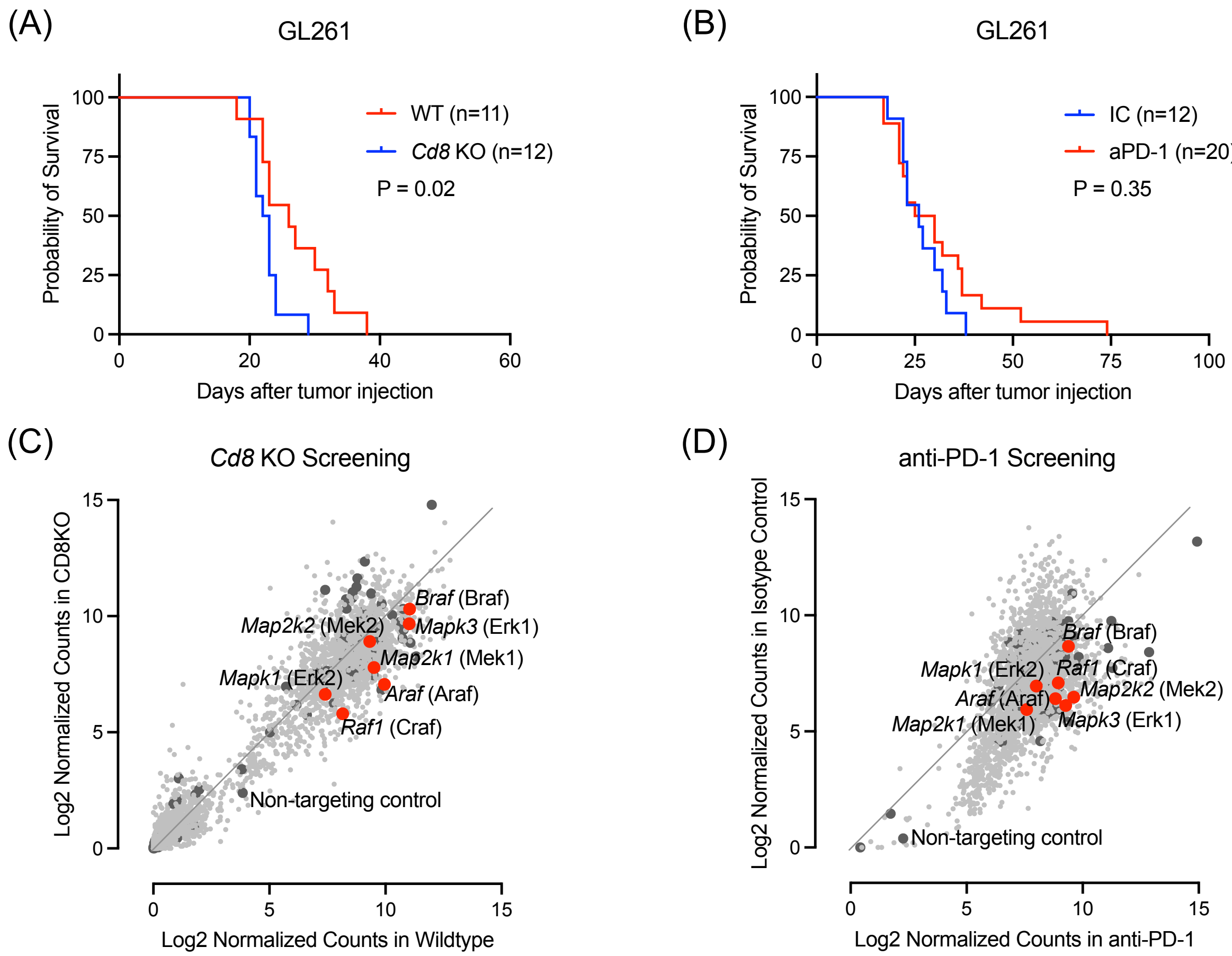

Supplementary Figure 1. (A) Kaplan-Meier survival curves from the kinome-wide CRISPR/Cas9 screen comparing GL261-bearing C57BL/6 wild-type (n = 11) and *Cd8* KO mice (n = 12). (B) Kaplan-Meier survival curves from the kinome-wide CRISPR/Cas9 screen conducted in C57BL/6 wild-type mice comparing isotype control treated (IC, n = 12) and anti-PD-1 treated (aPD-1, n = 20) treated groups. P-values were calculated using the log-rank test. (C) Overall scatter plot showing kinases with enriched or depleted sgRNAs in the wild-type (n = 11) compared to *Cd8* KO mice (n = 12). Red dots represent the MAPK/ERK pathway-targeting sgRNAs; gray dots indicate non-targeting control sgRNAs. (D) Overall scatter plot showing kinases with enriched or depleted sgRNAs in anti-PD-1-treated mice compared to controls (IC, n = 12; aPD-1, n = 20). Red dots represent the MAPK/ERK pathway-targeting sgRNAs; gray dots indicate non-targeting control sgRNAs. Source data are provided as a Source Data file.

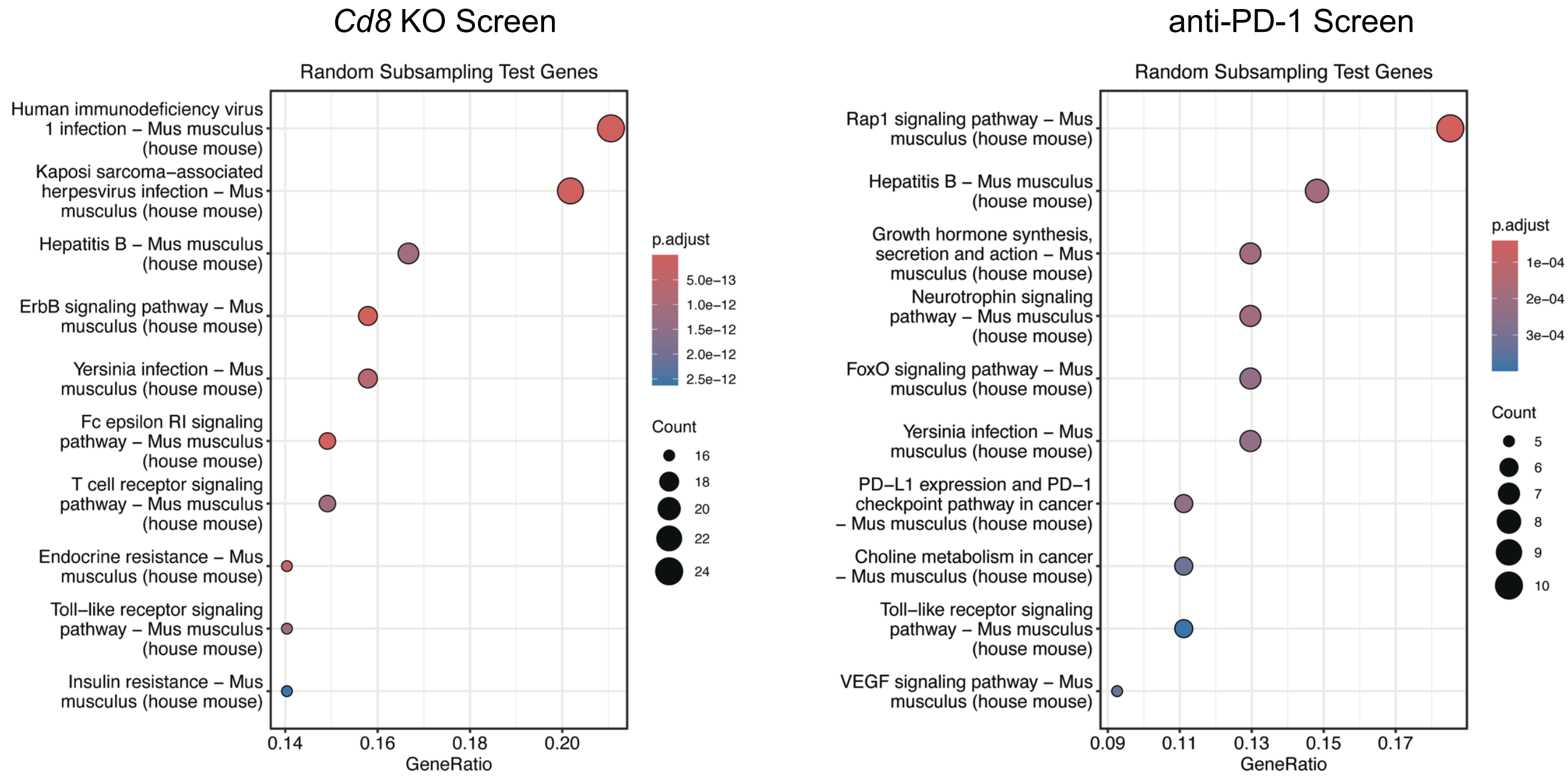

Supplementary Figure 2. Random permutation test of *Cd8* KO and anti-PD-1 CRISPR/Cas9 screen dataset (left: wild-type, n = 11; *Cd8* KO, n = 12; right: IC, n = 12; aPD-1, n = 20).

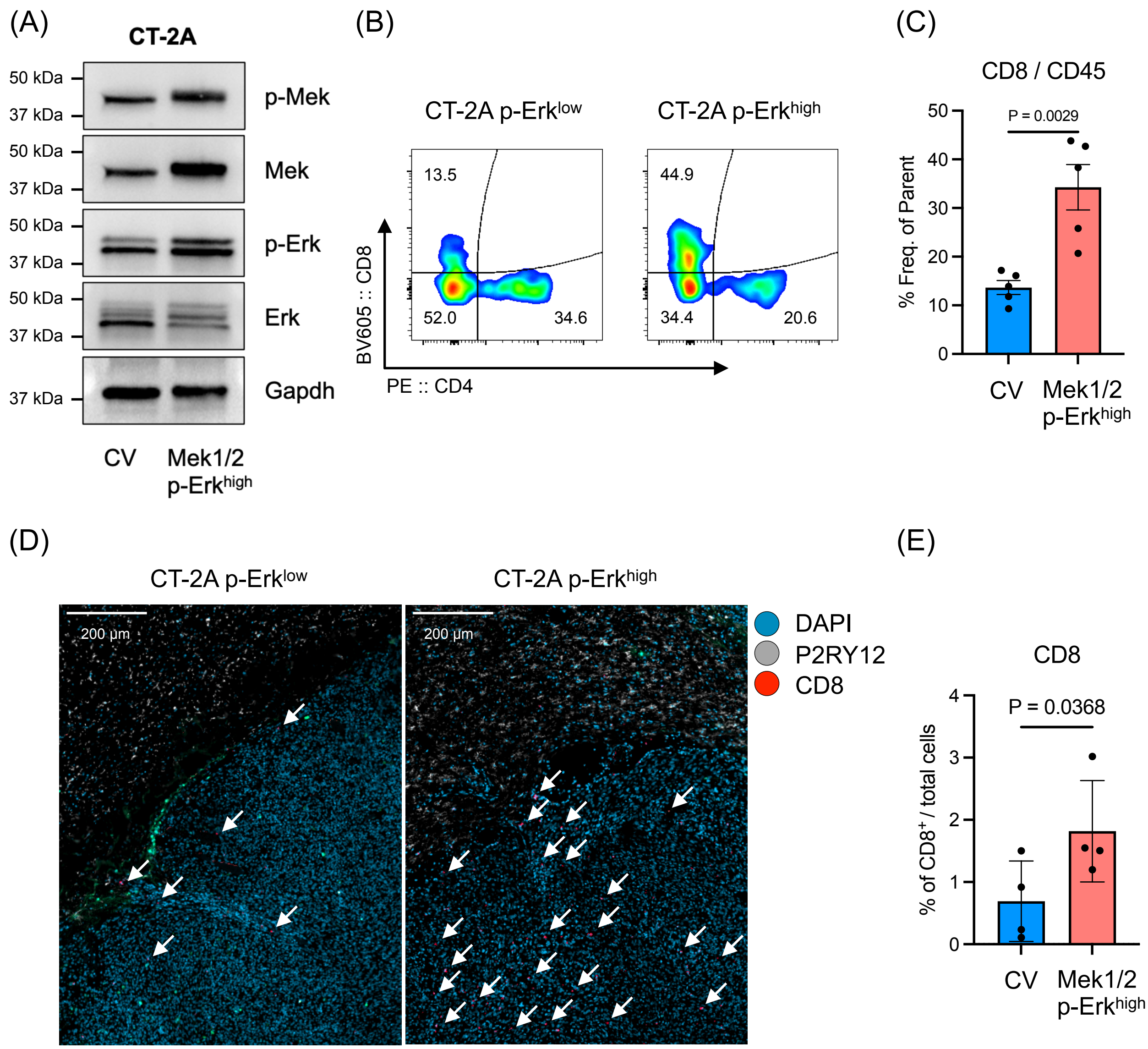

Supplementary Figure 3. Establishment of CT-2A Mek1/2 overexpression cell line. (A) Western blot analysis showing successful overexpression of Mek1 and Mek2 and subsequent activation of downstream MAPK/ERK signaling in CT-2A cells. Representative western blot from three independent experiments. (B) Representative flow cytometry plots of $CD8^+$ T cell infiltration in tumors derived from CT-2A CV (n = 5) and CT-2A Mek1/2 p-Erk^{high} (n = 5) cells post 21 days intracranial implantation. (C) Quantification of $CD8^+$ T cell frequency among $CD45^+$ immune cells in brain tumors from each group (CV, n = 5; Mek1/2 p-Erk^{high}, n = 5). (D) Representative multiplex immunofluorescence staining of tumor sections showing increased $CD8^+$ T cell infiltration in CT-2A Mek1/2 p-Erk^{high} tumors. Arrows indicate $CD8^+$ T cells. Scale bars = 200 µm. (E) Quantification of $CD8^+$ cell density in tumor sections (CV, n = 5; Mek1/2 p-Erk^{high}, n = 4). Each dot represents one animal. Data are shown as mean ± SEM. Statistical significance was determined using unpaired two-tailed Student's t-test. Source data are provided as a Source Data file.

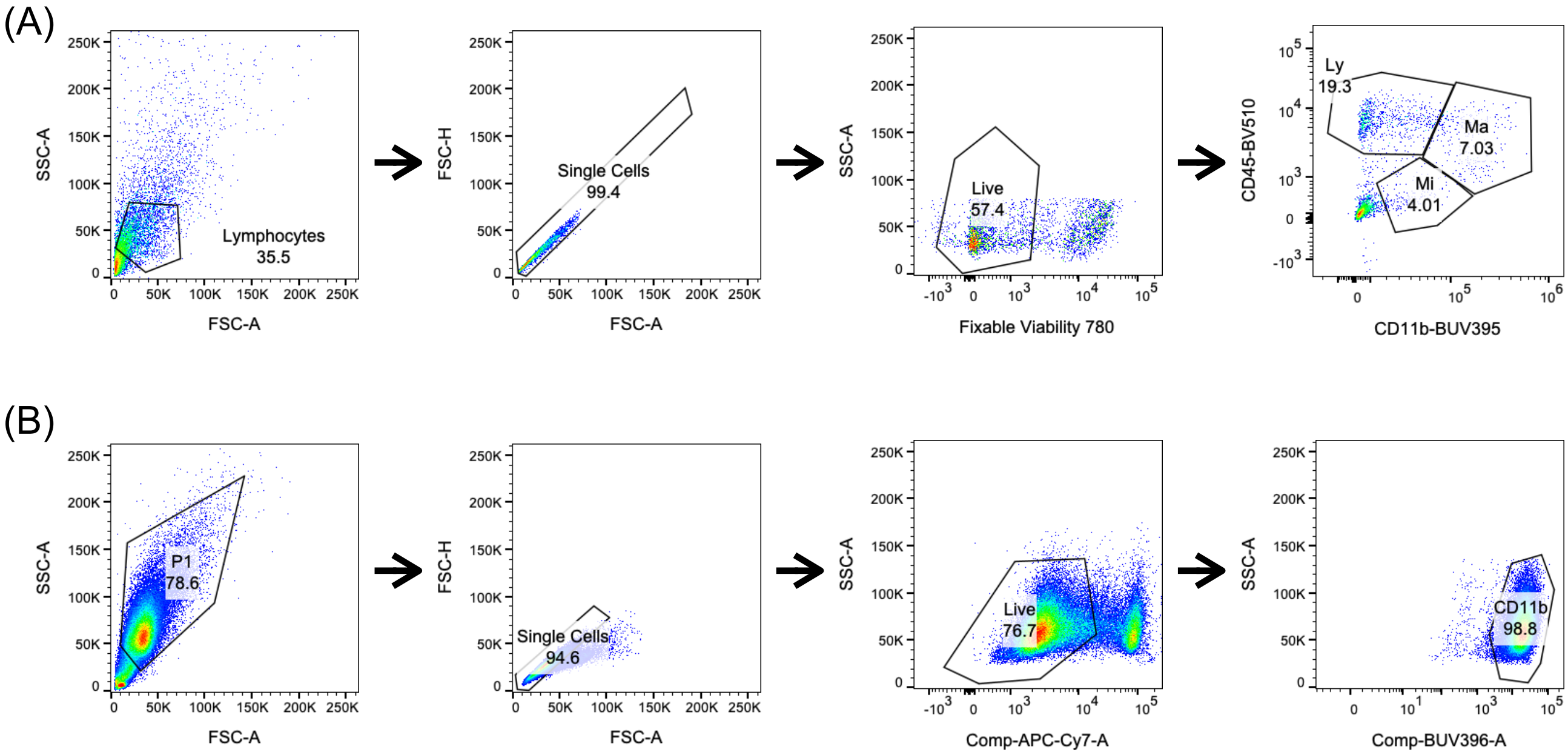

Supplementary Figure 4. Representative flow cytometry gating strategy. (A) Representative gating strategy used for flow cytometry analysis of tumor-infiltrating immune cells from intracranial mouse glioma tumors. Cells were sequentially gated to exclude debris, select singlets, and identify viable cells. Live single cells were then used to define lymphocyte, microglia, macrophage/myeloid. CD45+ lymphocytes were identified for further analysis. (B) Representative gating strategy used for flow cytometry analysis of *ex vivo* BMDM and TAM analysis. CD11b+ myeloid populations are defined for further analysis.

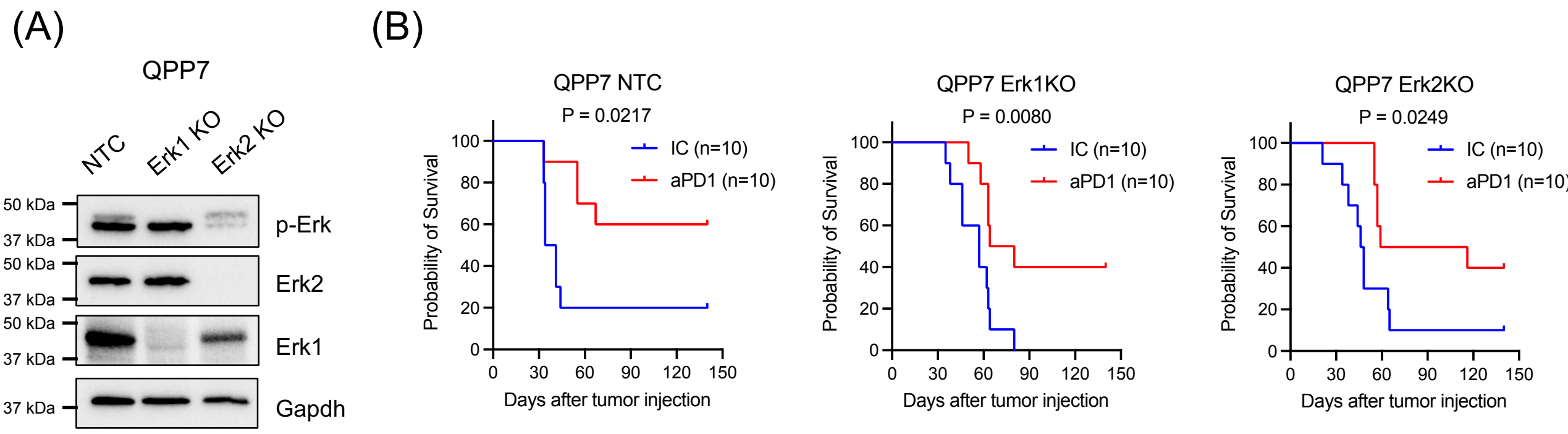

Supplementary Figure 5. (A) Western blot analysis showing KO of Erk1 or Erk2 in immunotherapy-sensitive QPP7 mouse glioma model. Representative western blot from three independent experiments. (B) Kaplan-Meier survival curves of QPP7-NTC bearing mice treated with IC (n = 10) or aPD-1 (n = 10). (C) Kaplan-Meier survival curves of QPP7-Erk1 KO bearing mice treated with IC (n = 10) or aPD-1 (n = 10). (D) Kaplan-Meier survival curves of QPP7-Erk2 KO bearing mice treated with IC (n = 10) or aPD-1 (n = 10). The P-values were calculated using the log-rank test. Source data are provided as a Source Data file.

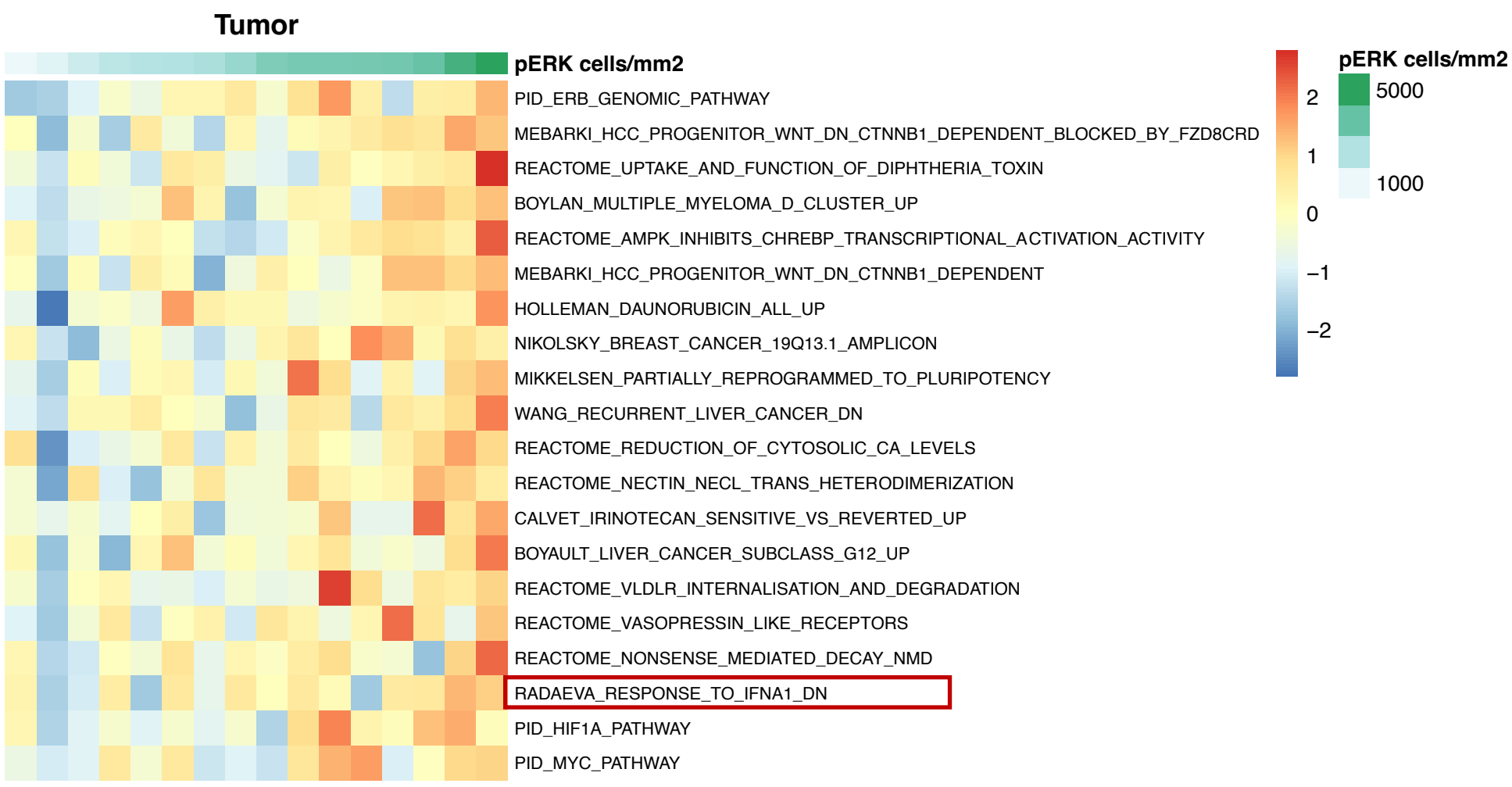

Supplementary Figure 6. Differential gene expression analysis of scRNA-seq data from human GB patient samples (n = 16), categorized by continuous levels of p-ERK.

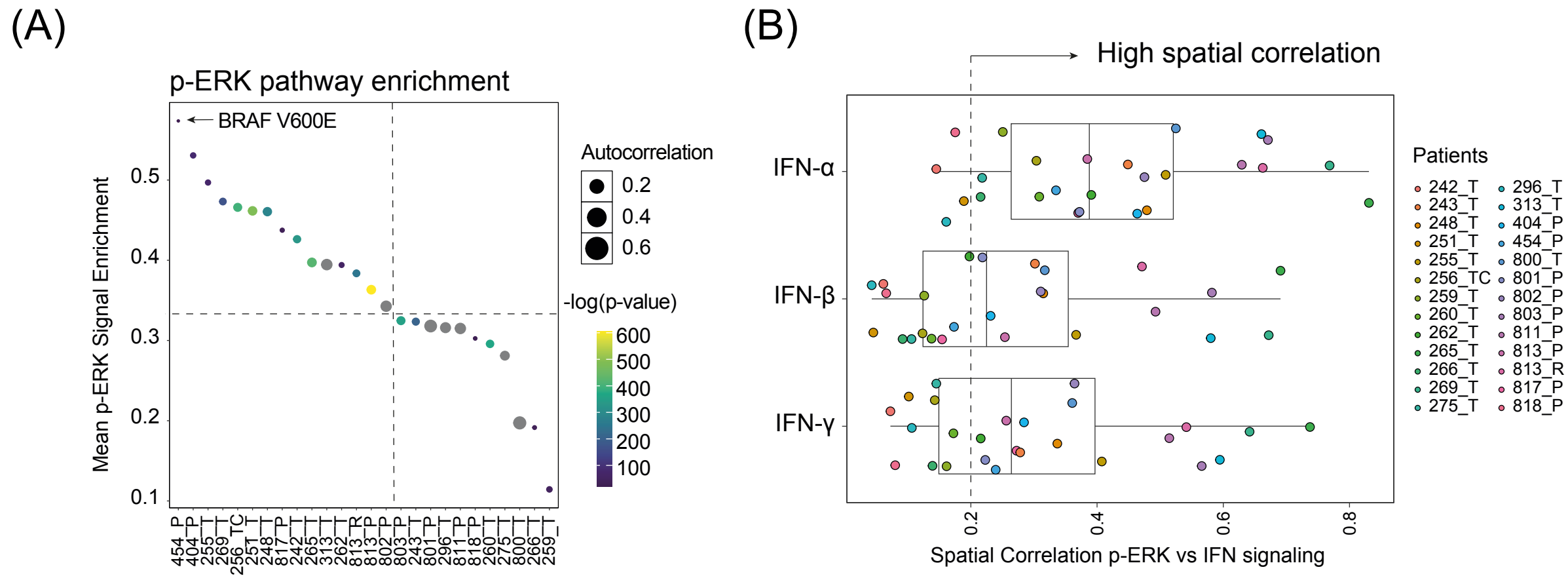

Supplementary Figure 7. (A) ERK pathway enrichment across samples of *de novo* IDH wild-type GBM (n = 26). Size indicates the level of spatial autocorrelation using Morans'I and the color encode the level of significance. (B) Spatial correlation of ERK activation and IFN signal enrichment. Analysis is derived from the Visium Gene Expression Slide platform.

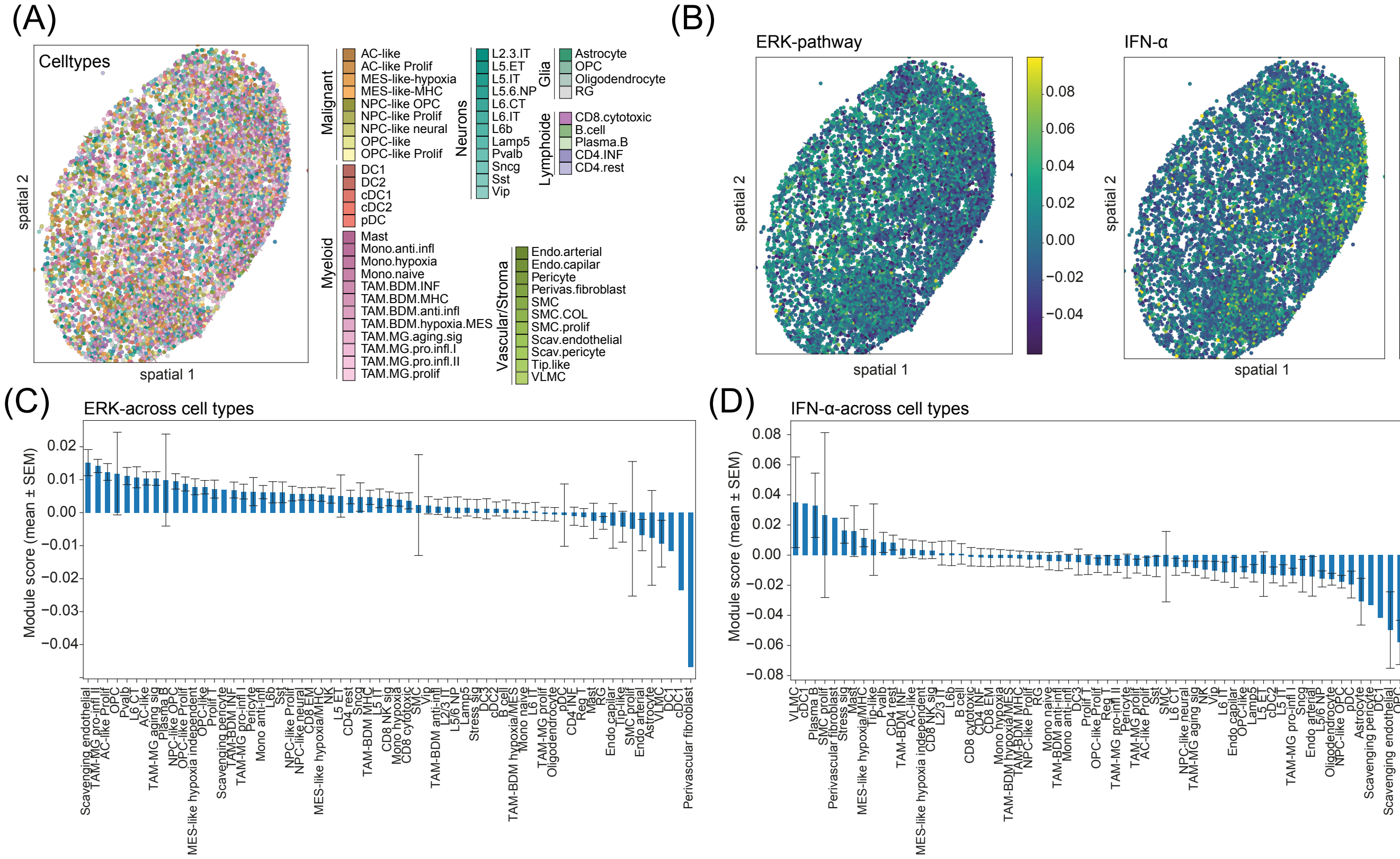

Supplementary Figure 8. (A) Illustration of an example using tissue microarrays (n = 6) of primary GBMs. Cell types were annotated based on the GBMap. (B) Cellular enrichment scores of ERK (left) and IFN-a pathway (right). Colors indicate the pathway enrichment score. (C) Mean ERK and IFN-a pathway enrichment (D) across samples and cell types. Analysis performed by MERFISH platform.

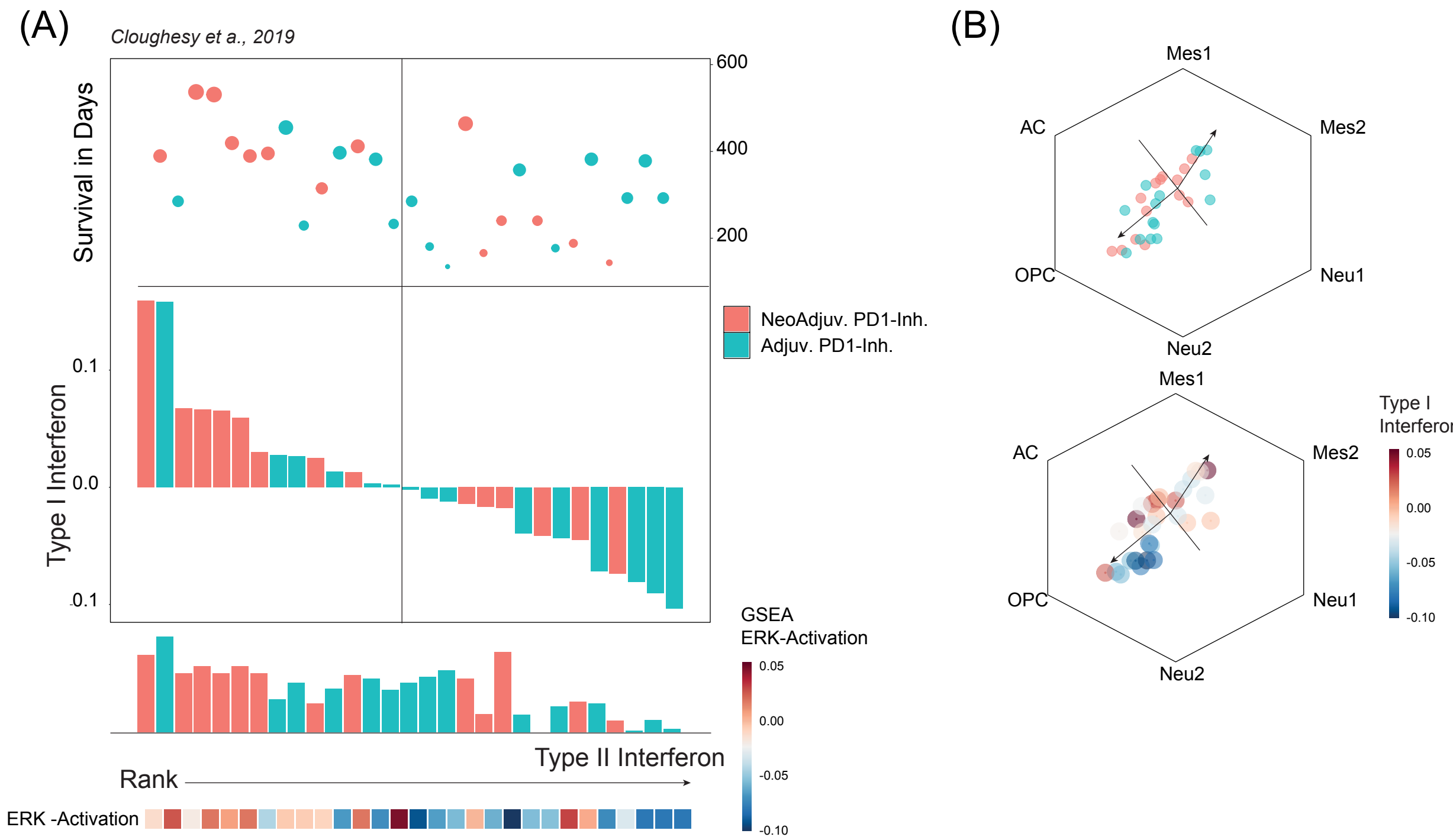

Supplementary Figure 9. (A) Analysis derived from the study by Cloughsey et al.[1], showing overall expression levels of Type I IFN across all cohorts and their respective survival rates. (B) The left panel depicts cell types and Type II interferon signaling, while the right side compares adjuvant PD-1 inhibitor therapy with neoadjuvant PD-1 inhibitor therapy.

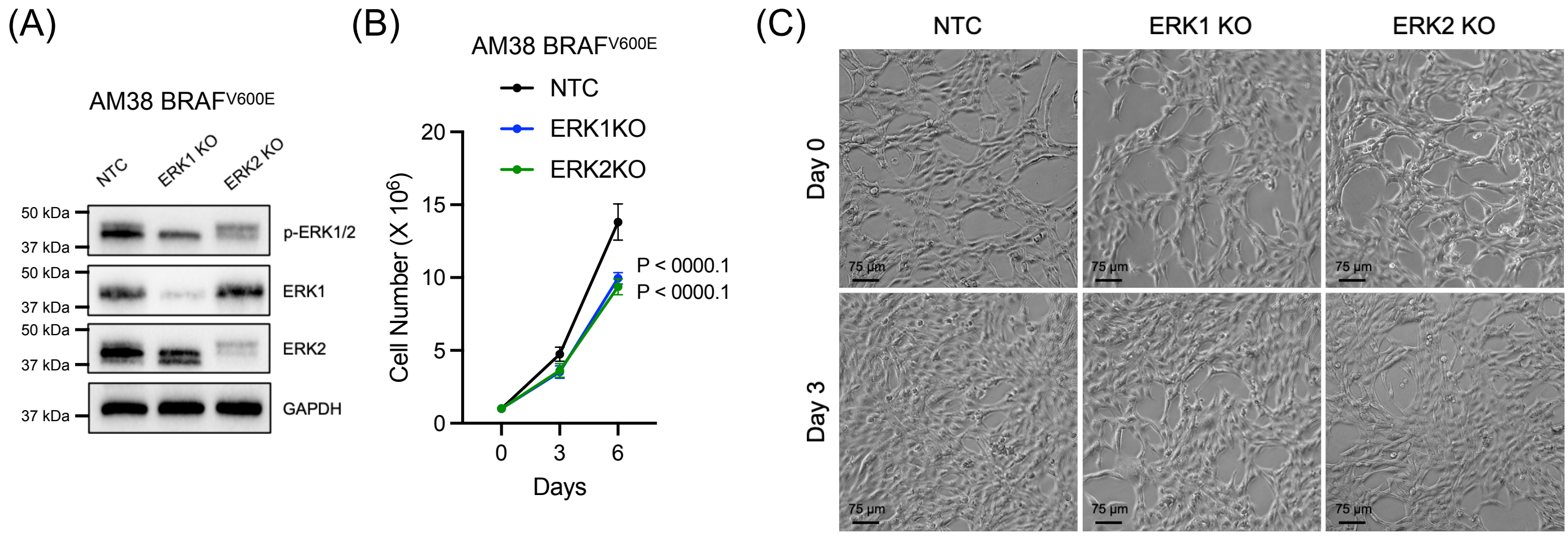

Supplementary Figure 10. Characterization of AM38-ERK KO cell lines. (A) Western blot analysis demonstrating the KO of ERK1 and ERK2 in the AM38 BRAF^{V600E} cell line. (B) Cell proliferation assay showing the total cell number count for both AM38 ERK1 and ERK2 KO cell lines over time. The graph represents the mean ± SD (n = 4), one-way ANOVA was used to determine statistical significance, and corresponding P-values are shown in the figure. (C) Representative images cell density of AM38 ERK1 and ERK2 KO cell lines. Scale bar = 75 µm. Source data are provided as a Source Data file.

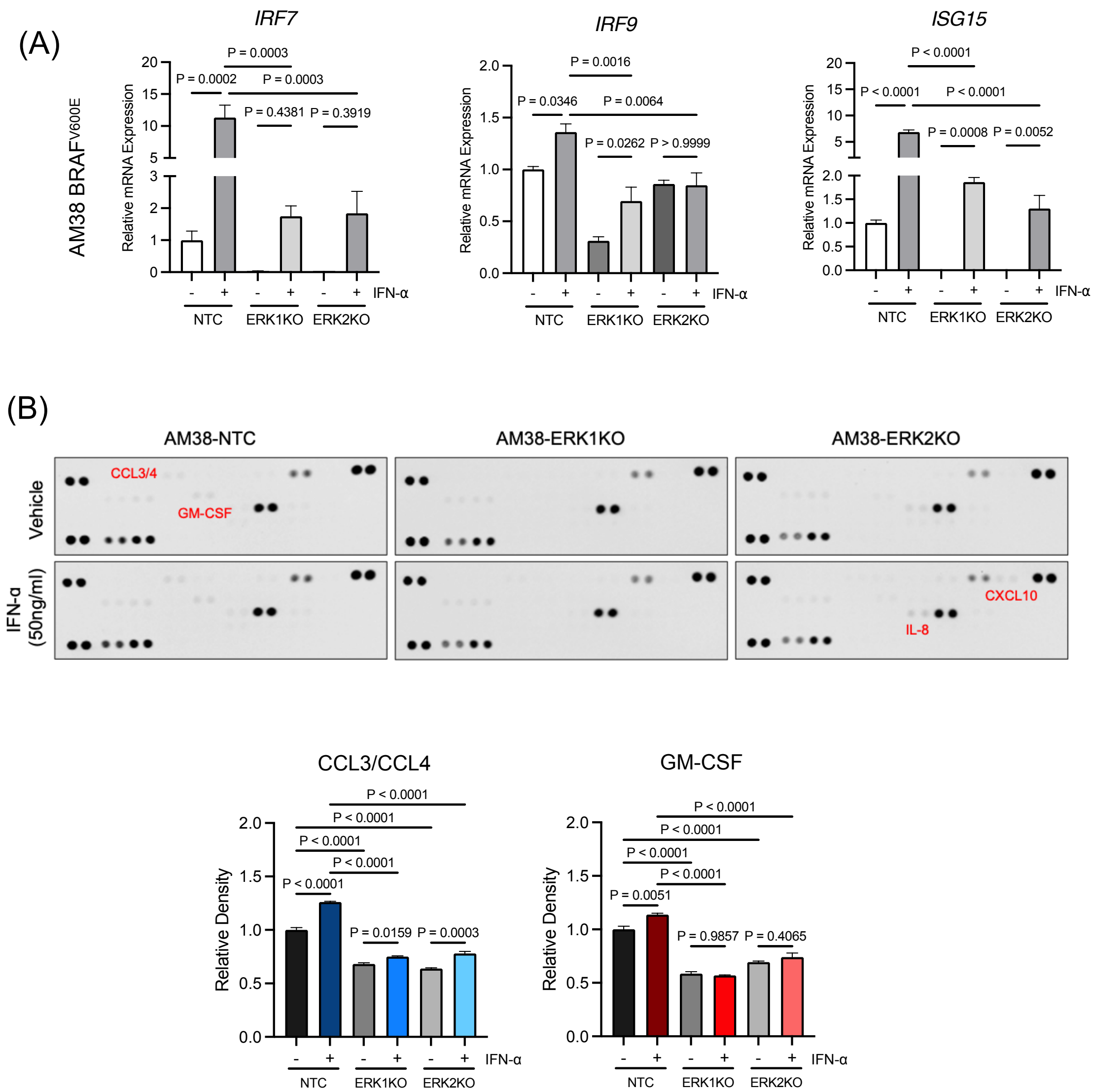

Supplementary Figure 11. (A) Quantitative RT-PCR results showing transcript levels of *IRF7*, *IRF9*, and *ISG15* in AM38-NTC, ERK1 KO, and ERK2 KO cells following exposure to IFN-α (20µg/ml). Data are presented as mean ± SD (n = 4). (B) Cytokine secretion analysis using a human cytokine array kit (top panel). Bar graphs represent the relative fold change in CCL3/CCL4 (left) and GM-CSF (right) secretion from the culture supernatants of AM38-NTC, ERK1 KO, and ERK2 KO cells. Statistical significance was determined using one-way ANOVA, and P-values are indicated. Source data are provided as a Source Data file.

(A)

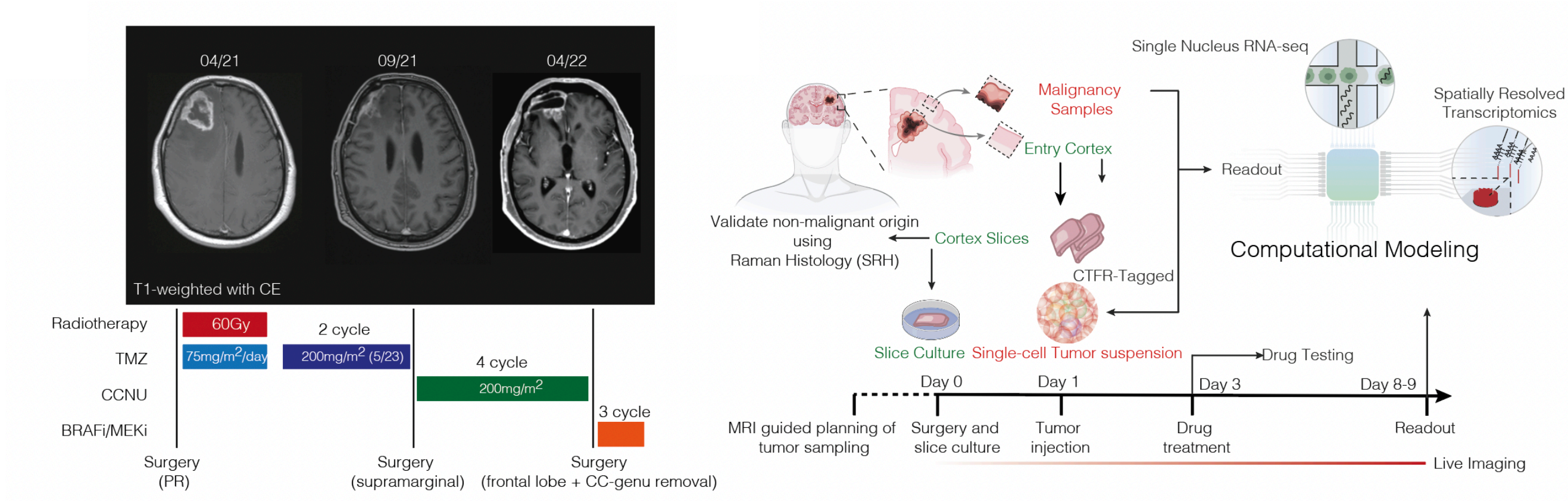

(B)

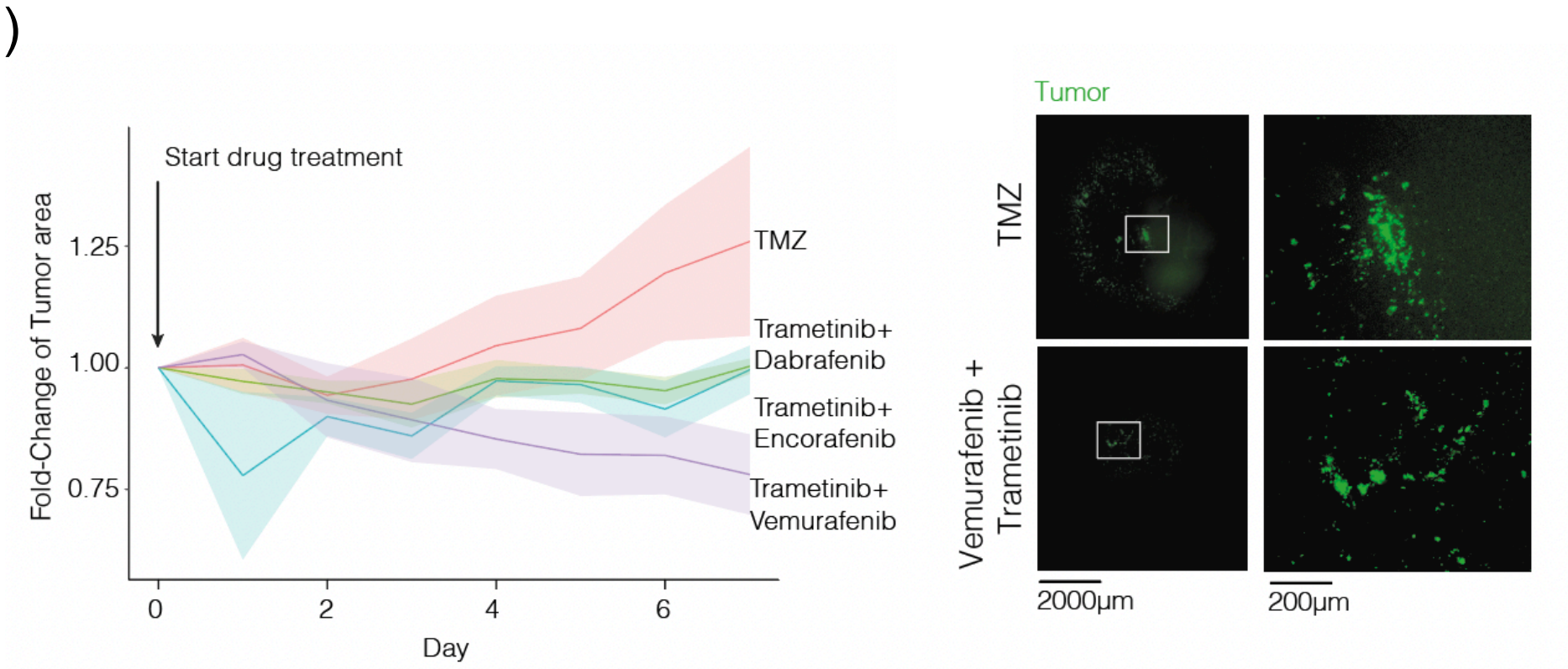

Supplementary Figure 12. (A) Brief illustration of autologous human neocortical slice model from *BRAF*^{V600E} mutated GBM patients. Tumor cells were implanted in cultivated cortex slices, then treated with TMZ or BRAFi/MEKi combination. Created in BioRender. Kim, K. (2026) https://BioRender.com/louom8t (B) The area covered by green fluorescence-labeled tumor cells was measured and ploted in the graph for slices treated with TMZ or various BRAFi/MEKi combinations. Representative fluorescence images show tumor cells growing on cortical slice treated with TMZ or Vemurafenib/Trametinib. Scale bar = 200 μm.

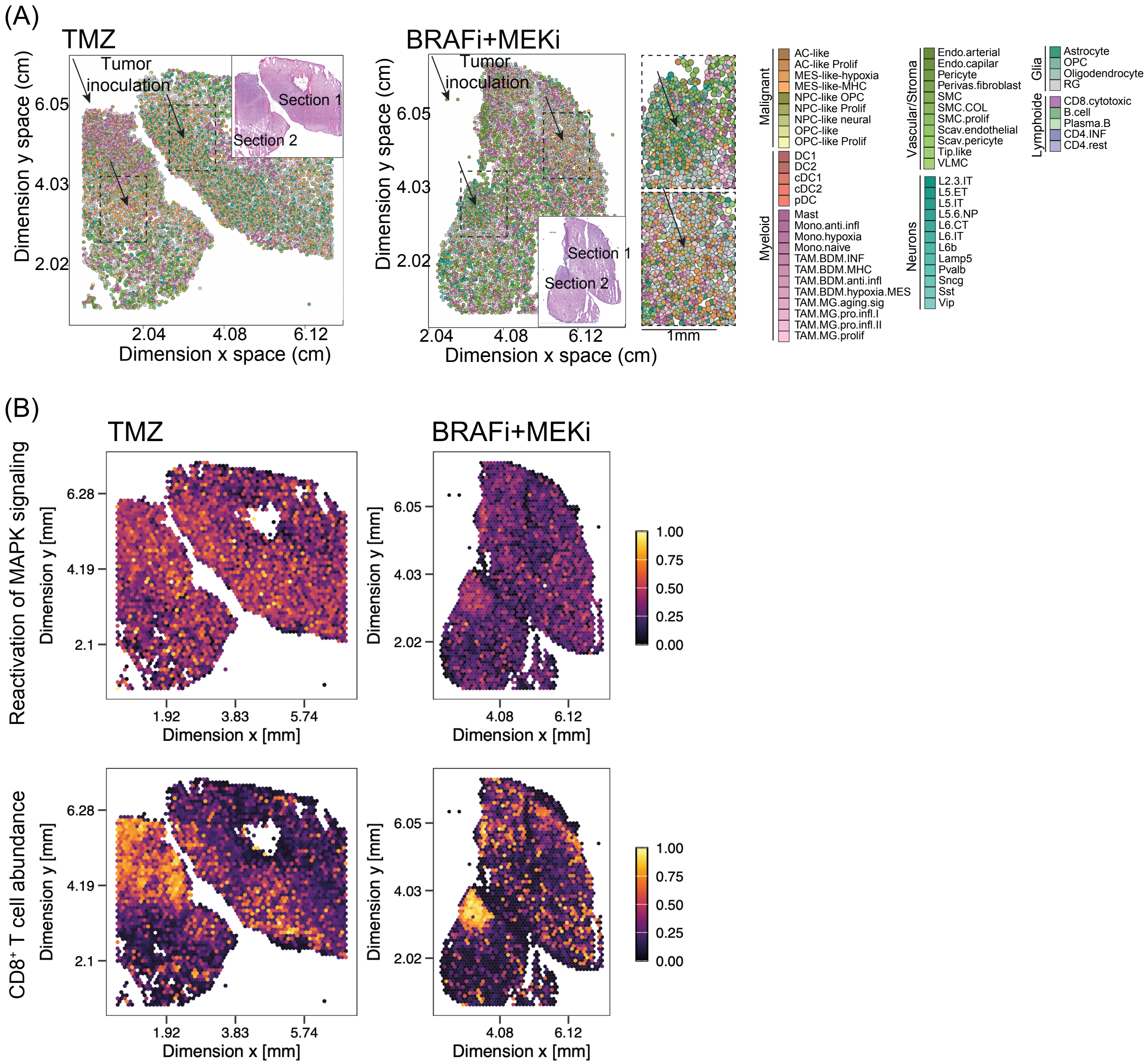

Supplementary Figure 13. (A) Spatial transcriptanalysis of slice culture of BRAFV600E GBM tumor treated with BRAFi/MEKi. One BRAFV600E GBM patient with 6 slices per condition (TMZ, Trametinib + Dabrafenib, Trametinib + Vemurafenib, Trametinib + Encorafenib), for a total of 30 quantified slices. Corresponding H&E images of the same tissue section are shown inset. Each color indicates the different cell types (right). (B) Spatial images of MAPK signaling (upper) and $CD8^{+}$ T cell abundance (lower) in slice culture samples treated with temozolomide (TMZ, left) or BRAFi/MEKi (right).

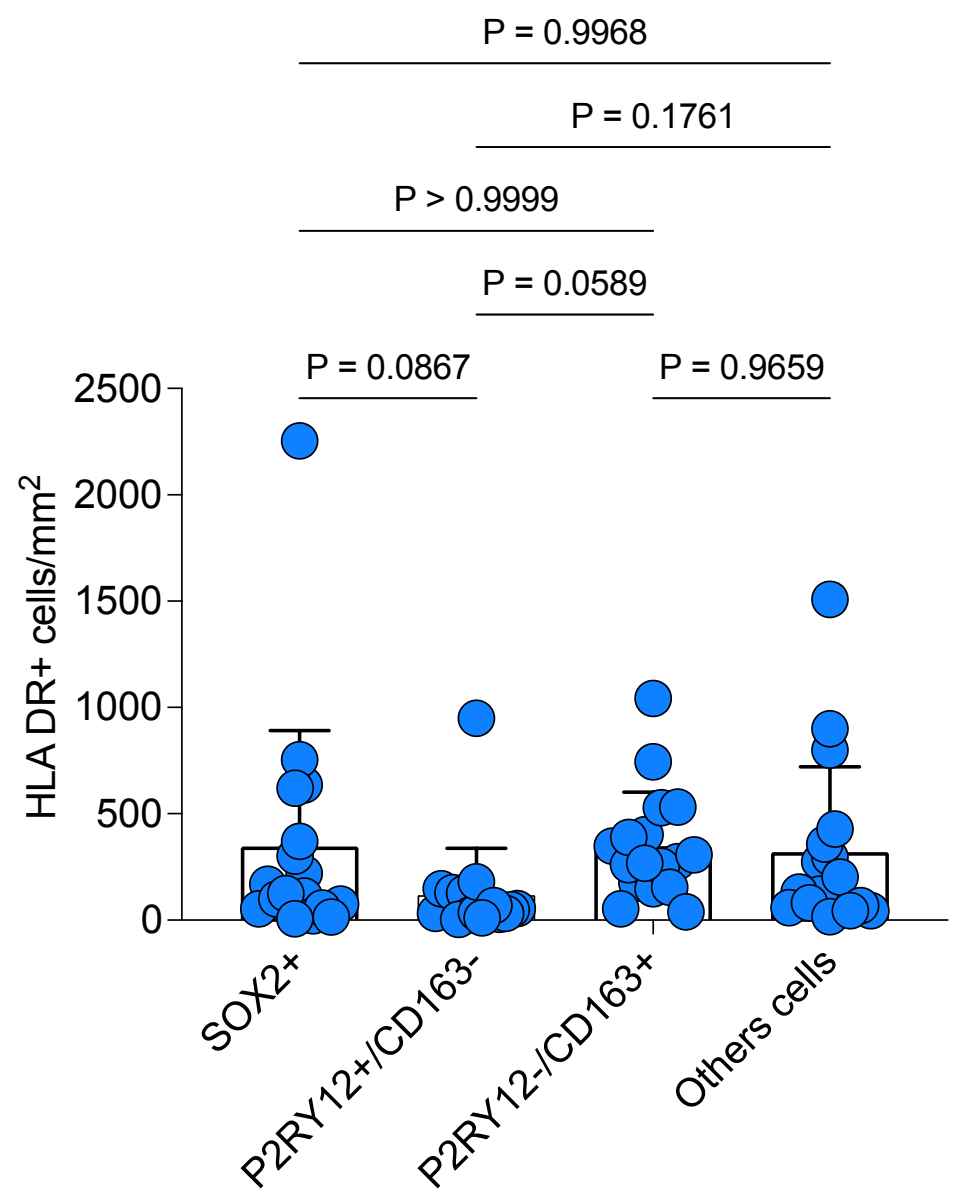

Supplementary Figure 14. Quantification analysis of immunofluorescence analysis staining for SOX2, P2RY12, CD163, HLA-DR, and DAPI in GB samples. One-way ANOVA was used to determine statistical significance, and corresponding P-values are shown in the figure. Source data are provided as a Source Data file.

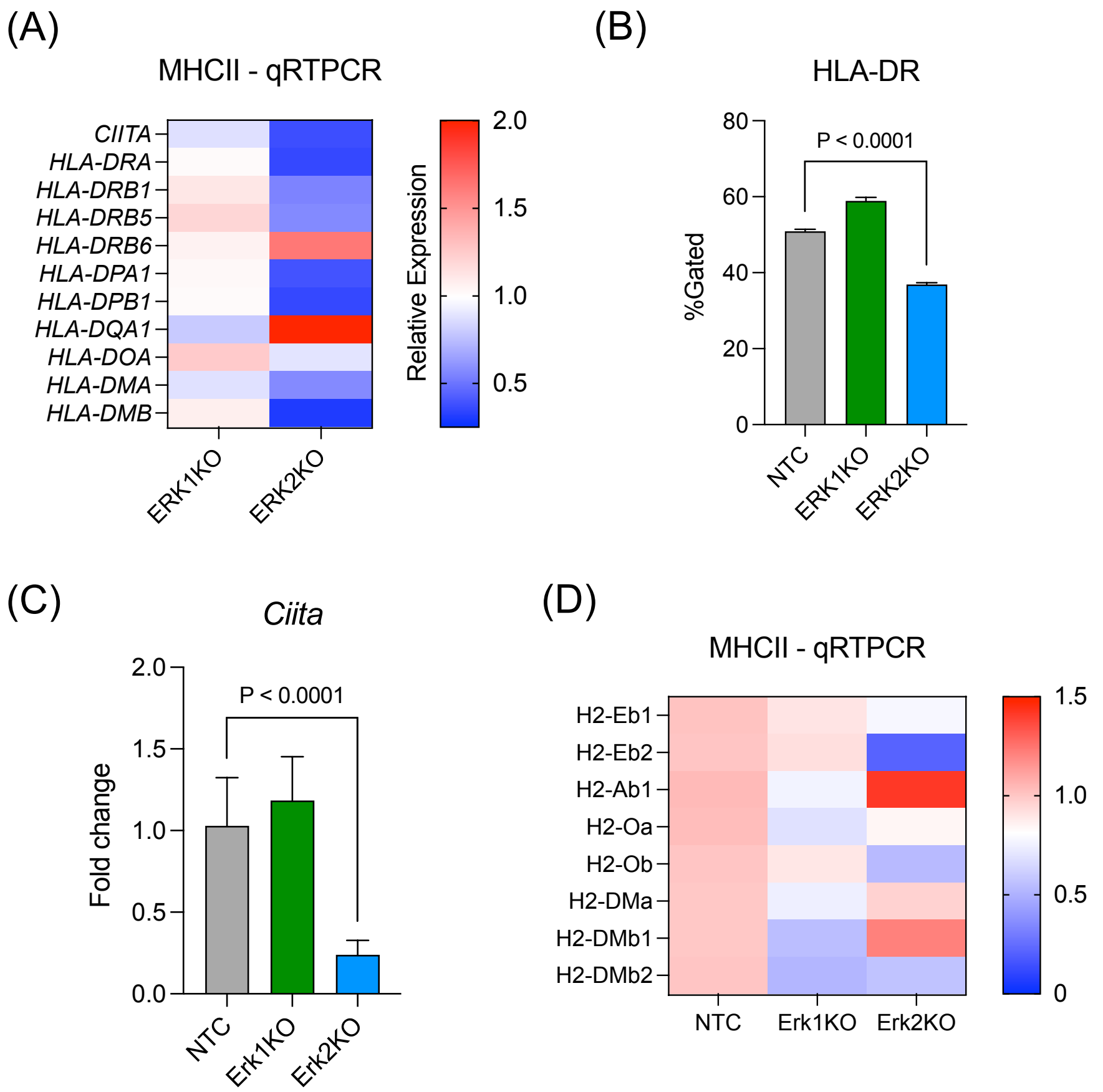

Supplementary Figure 15. (A) Quantitative RT-PCR results showing transcripts level of MHC class II genes comparing AM38-NTC cells and ERK1 KO or ERK2 KO cells. Data are presented as mean ± SD (n = 4). (B) HLA-DR protein expression was measured by flow cytometry-based analysis. Data are presented as mean ± SD (n = 4), unpaired two-tailed T test was used, and corresponding P-values are shown in the figure. (C) Quantitative RT-PCR results showing transcripts level of *Ciita* comparing QPP7-NTC cells and Erk1 KO or Erk2 KO cells. Data are presented as mean ± SD (n = 4), unpaired two-tailed T test was used. (D) Quantitative RT-PCR results showing transcripts level of MHC class II genes comparing QPP7-NTC cells and Erk1 KO or Erk2 KO cells. Data are presented as mean ± SD (n = 4), unpaired two-tailed T test was used, and corresponding P-values are shown in the figure. Source data are provided as a Source Data file.

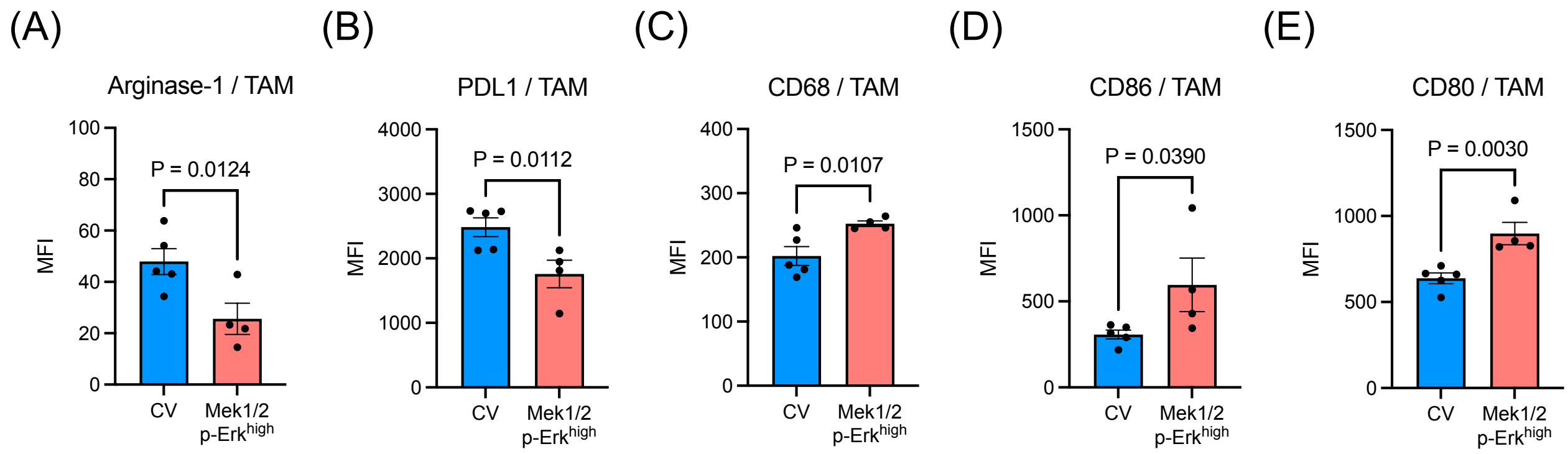

Supplementary Figure 16. Quantification of tumor associated myeloid cell markers ($CD11b^+/CD45^-$) on day 21 post tumor cell implantation from GL261-CV and GL261-Mek1/2 p-Erk^{high} gliomas. Flow cytometry analysis of tumor associated myeloid cells was conducted to evaluate immune-related marker expression in GL261-CV (n = 5) and GL261-Mek1/2 p-Erk^{high} (n = 4) tumor-bearing mice. Bar graphs show the frequency of (A) Arginase-1$^+$ cells, (B) PD-L1$^+$ cells, (C) CD68$^+$ cells, (D) CD86$^+$ cells, (E) CD80$^+$ cells, in CD11b$^+$ myeloid cells. Data are presented as mean ± SEM. Each dot represents an individual biological replicate. P values are depicted in the figures, unpaired two-tailed Student's t-test. Source data are provided as a Source Data file.

Reference

1. Cloughesy, T.F., Mochizuki, A.Y., Orpilla, J.R., Hugo, W., Lee, A.H., Davidson, T.B., Wang, A.C., Ellingson, B.M., Rytlewski, J.A., Sanders, C.M., et al. (2019). Neoadjuvant anti-PD-1 immunotherapy promotes a survival benefit with intratumoral and systemic immune responses in recurrent glioblastoma. Nature Medicine *25*, 477-+. 10.1038/s41591-018-0337-7.
