# Supplementary material for: Glioma-intrinsic MAPK/ERK signaling promotes immunotherapy efficacy through T cell infiltration and interferon responses
Source: Nat Commun. 2026 Jun 25;17:7968. doi: 10.1038/s41467-026-74124-7 (PMC13448493; doi:10.1038/s41467-026-74124-7)
Supplement: Supplementary file 2 [file 41467_2026_74124_MOESM2_ESM.pdf]

nature portfolio

Corresponding author(s): Adam M. Sonabend

Last updated by author(s): Jan 10, 2026

Reporting Summary

Nature Portfolio wishes to improve the reproducibility of the work that we publish. This form provides structure for consistency and transparency in reporting. For further information on Nature Portfolio policies, see our Editorial Policies and the Editorial Policy Checklist.

Statistics

For all statistical analyses, confirm that the following items are present in the figure legend, table legend, main text, or Methods section.

n/a	Confirmed	
☐	☒	The exact sample size (*n*) for each experimental group/condition, given as a discrete number and unit of measurement
☐	☒	A statement on whether measurements were taken from distinct samples or whether the same sample was measured repeatedly
☐	☒	The statistical test(s) used AND whether they are one- or two-sided<br>*Only common tests should be described solely by name; describe more complex techniques in the Methods section.*
☐	☒	A description of all covariates tested
☐	☒	A description of any assumptions or corrections, such as tests of normality and adjustment for multiple comparisons
☐	☒	A full description of the statistical parameters including central tendency (e.g. means) or other basic estimates (e.g. regression coefficient) AND variation (e.g. standard deviation) or associated estimates of uncertainty (e.g. confidence intervals)
☐	☒	For null hypothesis testing, the test statistic (e.g. *F*, *t*, *r*) with confidence intervals, effect sizes, degrees of freedom and *P* value noted<br>*Give P values as exact values whenever suitable.*
☐	☒	For Bayesian analysis, information on the choice of priors and Markov chain Monte Carlo settings
☐	☒	For hierarchical and complex designs, identification of the appropriate level for tests and full reporting of outcomes
☐	☒	Estimates of effect sizes (e.g. Cohen's *d*, Pearson's *r*), indicating how they were calculated

Our web collection on statistics for biologists contains articles on many of the points above.

Software and code

Policy information about availability of computer code

Data collection	The dataset from CRISPR/Cas9 screen in CD8KO mice is published and available under BioProject ID PRJNA822842. The CRISPR/Cas9 screen data using PD-1 blockade is available under BioProject ID PRJNA1164273. The repository contained the RNA-seq data from the ERK-KO, single-cell data from the Patient's dataset, the single cell data from the neocortical slice model, the Visium Data from the slice experiment has been deposited on the OSF platform (https://osf.io/5947q/?view_only=a7939920e2e5490dbd9e8cbdf273c811). Publicly available GBmap dataset can be found via cellxgene (https://cellxgene.cziscience.com/collections/999f2a15-3d7e-440b-96ae-2c806799c08c).
Data analysis	Both CD8KO and anti-PD-1 kinome-wide CRISPR screens were analyzed using the Model-based Analysis of Genome-wide CRISPR-Cas9 Knockout (MAGeCK) computational tool. KEGG Pathway enrichment analysis was performed on significant genes (FDR < 0.05) in R (v4.2.3) using clusterProfiler (v4.12.0) package and plots were generated using enrichPlot (v1.24.0). Multiplex analysis was processed in R using R packages Phenoptr and PhenoptrReports to merge and create consolidated single files for each tumor sample. Consolidated files had cell phenotypes as outputs that we employed for further quantification and spatial analyses using the Phenoptr R addin.

For manuscripts utilizing custom algorithms or software that are central to the research but not yet described in published literature, software must be made available to editors and reviewers. We strongly encourage code deposition in a community repository (e.g. GitHub). See the Nature Portfolio guidelines for submitting code & software for further information.

Data

Policy information about availability of data

All manuscripts must include a data availability statement. This statement should provide the following information, where applicable:

- Accession codes, unique identifiers, or web links for publicly available datasets
- A description of any restrictions on data availability
- For clinical datasets or third party data, please ensure that the statement adheres to our policy

The dataset from CRISPR/Cas9 screen in Cd8 KO mice is published and available under BioProject ID PRJNA822842 [https://www.ncbi.nlm.nih.gov/bioproject/PRJNA822842/]. The CRISPR/Cas9 screen data using PD-1 blockade is available under BioProject ID PRJNA1164273 [https://www.ncbi.nlm.nih.gov/bioproject/PRJNA1164273].
Single-cell data from the patient dataset, single-cell data from the neocortical slice model, and Visium data from the slice experiment have been deposited in public repositories. The processed BRAF patient-derived Visium and scRNA-seq datasets generated in this study have been deposited in the Gene Expression Omnibus under accession number GSE331374 [https://www.ncbi.nlm.nih.gov/geo/query/acc.cgi?acc=GSE331374]. Raw sequencing files from human/patient-derived samples were not deposited in an unrestricted public repository due to privacy and ethical restrictions related to potentially identifiable human genetic information. Access requests should be directed to the corresponding author and will be reviewed subject to institutional approval and/or a data use agreement. The remaining patient Visium datasets were previously published and are publicly available through Dryad at https://doi.org/10.5061/dryad.h70rxwdmj. Publicly available GBMmap dataset can be found via cellxgene (https://cellxgene.cziscience.com/collections/999f2a15-3d7e-440b-96ae-2c806799c08c)34. The remaining data are available within the Article, Supplementary Information or Source Data file. Source data are provided with this paper.

Research involving human participants, their data, or biological material

Policy information about studies with human participants or human data. See also policy information about sex, gender (identity/presentation), and sexual orientation and race, ethnicity and racism.

Reporting on sex and gender	Analyses were not designed to assess sex-specific differences, and sex was not used as a biological variable in the primary analyses.
Reporting on race, ethnicity, or other socially relevant groupings	Race, ethnicity, and other socially relevant grouping information was not available for the de-identified human glioma specimens and was therefore not analyzed.
Population characteristics	Human glioma specimens were obtained from patients diagnosed with glioblastoma or glioma who underwent surgical resection. Clinical and pathological information, when available, was obtained from associated de-identified clinical metadata.
Recruitment	The tumor samples from the discovery cohort were compiled retrospectively. No active recruitment was conducted during this study. All the relevant covariants are tested for any possible confounding effect. For the validation cohort, participants were recruited by site-specific co-investigators at their respective institutions based on protocol eligibility criteria and verified by the study coordinator. Self-selection bias may be present and may affect survival; however, we specifically analyzed the control arm of the indicated clinical trial employing the same methodology as the discovery cohort.
Ethics oversight	Institutional review board (IRB) approval was acquired from Northwestern University (STU00095863).<br>The ethics approval was issued by the local committee of the University of Freiburg for data evaluation, imaging procedures and experimental design (Freiburg: protocol 100020/09 and 472/15_160880).

Note that full information on the approval of the study protocol must also be provided in the manuscript.

Field-specific reporting

Please select the one below that is the best fit for your research. If you are not sure, read the appropriate sections before making your selection.

☒ Life sciences ☐ Behavioural & social sciences ☐ Ecological, evolutionary & environmental sciences

For a reference copy of the document with all sections, see nature.com/documents/nr-reporting-summary-flat.pdf

Life sciences study design

All studies must disclose on these points even when the disclosure is negative.

Sample size	We performed survival studies with 10 mice per group to get enough power for statistic analysis. For glioma infiltrating immune cell phenotyping, we analyzed with 5 samples for each group.
Data exclusions	We did not exclude any sample or group in this study.
Replication	We established multiple clones of overexpression cell lines and confirmed the phenotype with at least 3 different clones.
Randomization	All animal experiments were done in randomized and blinded grouping.
Blinding	All animal experiments were done in randomized and blinded grouping.

Reporting for specific materials, systems and methods

We require information from authors about some types of materials, experimental systems and methods used in many studies. Here, indicate whether each material, system or method listed is relevant to your study. If you are not sure if a list item applies to your research, read the appropriate section before selecting a response.

Materials & experimental systems

n/a	Involved in the study
☐	☒ Antibodies
☐	☒ Eukaryotic cell lines
☒	☐ Palaeontology and archaeology
☐	☒ Animals and other organisms
☐	☒ Clinical data
☒	☐ Dual use research of concern
☒	☐ Plants

Methods

n/a	Involved in the study
☒	☐ ChIP-seq
☐	☒ Flow cytometry
☒	☐ MRI-based neuroimaging

Antibodies

Antibodies used	Biolegend antibodies, CTLA4-PerCP5.5 (Biolegend 106316), FOXP3-AF488 (Biolegend 320012), CD62L-PE-Cy7 (Biolegend 104418), CD4-PE (Biolegend 100408), CD44-BV711 (Biolegend 103057), CD8a-BV605 (Biolegend 100744), CD45-BV510 (Biolegend 103138), CD11b-BUV395 (BD 563553), PD1-PEFire640 (Biolegend 135258). Multiplex antibodies, TMEM119 (HPA051870, Sigma-Aldrich, 1/250 dilution) with Opal 570 (1/200 dilution), GFAP (ab68428, clone EPR1034Y, Abcam, 1:400 dilution) with Opal 620, HLA-DR (cat ab20181, clone TAL 1B5, Abcam, 1:1000 dilution) with Opal 690 (1:200 dilution)
Validation	We validated the antibodies for multiplex analysis and we depicted the dilution factor for each antibody. For flow cytometry, we follow the manufacturer's instruction.

Eukaryotic cell lines

Policy information about cell lines and Sex and Gender in Research

Cell line source(s)	The mouse glioma cell lines GL261 was obtained from the National Institutes of Health. QPP7 cells, kindly provided by Dr. Amy B. Heimberger from Northwestern University.
Authentication	All the cells were tested for mycoplasma STR profile and confirmed contamination-negative before intracranial injection.
Mycoplasma contamination	All the cells were tested for mycoplasma STR profile and confirmed contamination-negative before intracranial injection.
Commonly misidentified lines (See ICLAC register)	n/a

Animals and other research organisms

Policy information about studies involving animals; ARRIVE guidelines recommended for reporting animal research, and Sex and Gender in Research

Laboratory animals	We used C57BL/6 mice for survival studies and glioma infiltrating immune cell phenotyping.
Wild animals	n/a
Reporting on sex	Biological variables such as sex representation, age, and littermate controls will be kept same across control and test groups. Results will be reported separately for each sex. If analyses suggest differences between sexes, we will use this information to calculate sample size and perform adequately powered experiments to rigorously explore the differences between sexes.
Field-collected samples	n/a
Ethics oversight	All mouse work performed in this study were approved by the Institutional Animal Care and Use Committee (IACUC) under protocol number IS00014080.

Note that full information on the approval of the study protocol must also be provided in the manuscript.

Clinical data

Policy information about clinical studies

All manuscripts should comply with the ICMJE guidelines for publication of clinical research and a completed CONSORT checklist must be included with all submissions.

Clinical trial registration	n/a
Study protocol	n/a
Data collection	n/a
Outcomes	n/a

Plants

Seed stocks	n/a
Novel plant genotypes	n/a
Authentication	n/a

Flow Cytometry

Plots

Confirm that:

- [x] The axis labels state the marker and fluorochrome used (e.g. CD4-FITC).
- [x] The axis scales are clearly visible. Include numbers along axes only for bottom left plot of group (a 'group' is an analysis of identical markers).
- [x] All plots are contour plots with outliers or pseudocolor plots.
- [x] A numerical value for number of cells or percentage (with statistics) is provided.

Methodology

Sample preparation	Single cells enriched by Percoll gradient are prepared from the brains of glioma-bearing mice.
Instrument	BD Symphony
Software	FlowJo 10.8.1
Cell population abundance	We did not use sorter in this manuscript.
Gating strategy	FSC and SSC were used to gate for cell size, granularity, and single cell, while live/dead discrimination was performed using APC-Cy7. CD45 was used to identify leukocytes, CD11b for myeloid cells, CD8 and CD4 for T-cell subsets.

- [x] Tick this box to confirm that a figure exemplifying the gating strategy is provided in the Supplementary Information.
