# Supplementary material for: Glioma-intrinsic MAPK/ERK signaling promotes immunotherapy efficacy through T cell infiltration and interferon responses
Source: Nat Commun. 2026 Jun 25;17:7968. doi: 10.1038/s41467-026-74124-7 (PMC13448493; doi:10.1038/s41467-026-74124-7)
Supplement: Supplementary file 3 [file 41467_2026_74124_MOESM3_ESM.pdf]

nature portfolio

Peer Review File

Glioma-intrinsic MAPK/ERK signaling promotes immunotherapy efficacy through T cell infiltration and interferon responses

Corresponding Author: Dr Adam Sonabend

This file contains all reviewer reports in order by version, followed by all author rebuttals in order by version.

Version 0:

Reviewer comments:

Reviewer #1

(Remarks to the Author)
The rationale for this study is based on the observation that MAPK/ERK signaling is associated with overall survival following immune checkpoint blockade (with anti-PD-1 or anti-CTLA-4) in recurrent glioblastomas (GB). The authors capitalize on the results of the previous study in which they performed the kinome-wide CRISPR/Cas9 screening in murine GL261 gliomas and identified Chek2 as main regulator in CD8KO cells (doi: 10.1038/s41467-023-36878-2.).
In the present study the authors applied similar CRISPR/Cas9 screening to murine 261 glioma cells to identify key regulators in CD8 KO and anti-PD-1 treated mice. This time the Gene ontology (GO) analysis revealed Map2k2 (encoding Mek2) and Araf from the MAPK pathway as the most significantly enriched among all kinase pathways in CD8 KO, and Araf, Map2k1 (encoding Mek1) and Erk2 in anti-PD-1 treated mice. Moreover, Mek1/2 Erkhigh tumors respond well to CTLA4 blockade.. Knockout of Erk1 or Erk2 in murine QPP7 gliomas , sensitive to anti-PD1, produced same responses as in control gliomas.
The validation part encompasses overexpression of Mek1 or Mek2, implanting cells and seeking effects on survival. They studied CD8+ T cell responses and survival to validate selected genes. Experimentally-induced ERK phosphorylation in glioma cells enhanced survival after anti-PD1 treatment, led to anti-tumoral immunity upon re-challenge and increased infiltration of memory T cell in long-term survivors. Spatial transcriptomics and scRNA-seq analysis revealed the modulation of interferon responses by the MAPK/ERK pathway in BRAFV600E human GB cells with ERK1/2 knockout and in slice cultures of human BRAFV600E GB tissue. Ex vivo a BRAFV600E mutant GB tumor slices treated with inhibitors (BRAFi/MEKi) demonstrated links between tumor cell immunogenicity. They claimed that BRAFi/MEKi treatment disrupted the interaction between tumor cells and tumor-associated macrophages/microglia in slice cultures from BRAFV600E mutant GB.
Altogether, the results are intriguing, against what to be expected about roles of IFNs in antitumor responses.The results are significant to the field of tumor immunology. The authors used a plethora of methods to verify their conclusions. The study demonstrates the particular mechanism underpinning the resistance of GB immune checkpoint blockade using murine glioma cells overexpressing Mek1/2 with high ERK activation, which extends to BRAFV600E mutant GB cells and ex vivo slices. Potential mechanisms have been verified. The data are novel and the findings are in most aspects solid and well presented. Some parts of the manuscript may benefit from more detailed explanation of some results. Technically, the proper state of the art methods have been applied. A number of animals in experiments is sufficient and proper statistical analyses were employed when necessary. In some aspects the results and their interpretation is a bit confusing. The methodology sound and this work meets the expected standards in your field?
There are enough detaila provided in the methods to reproduce the work. There are minor shortcomings that could be improved by a proper revision or re-writing.
Major comments:
1. Fig.2. The authors overexpressed Mek1 and 2 to achieve high Erk activation and such enhancement of MAPK/ERK signaling increased CD19 fraction (consistent with higher CD8+ cell infiltration) and improved survival after anti-PD1 treatment. The appearance of Mek1/2 Erklow tumors is surprising and confusing, and it could be may be reasons that they respond differently to therapy. Basal p-Erk in suddenly high in those cells in contrast to fig.2A.
2. There are some inconsistencies in detecting Erk/pErk. There is discrepancy in basal p-Erk levels between different panels. Fig.2. A. F- The part A shows low level of p-Erk in control, but it is much higher in the part F, where Mek1/2Erk low cells are presented. Supplementary S2. Why Western blot shows a single Erk1 or Erk2 band while there should be two

bands?
3. Fig.3. They wrote: "In the GL261 mouse glioma model, resistant to both conventional and Fc-enhanced aCTLA-4, activation of the MAPK signaling led to improved efficacy of Fc-enhanced aCTLA-4 therapy". But the data on the figure shows the significant positive effect of the Fc-enhanced aCTLA-4. Please be precise in description of the results.
4. Fig.4. Regarding the selection of tumor areas with low or high p-Erk staining. The authors state: "Tumors with high p-ERK exhibited a significantly higher density of SOX2+p-ERK+IRF9+ tumor cells compared to low p-ERK tumors" and quantification shows a number of positive cells per mm2. But what about tumor cell density in those areas? IHC staining in the A panel C seems to show low cell density. High resolution picture should be demonstrated to show co-localization of IRF9 and p-Erk, as it is, it is not convincing.
5. Fig.4D-F. The description of findings from spatial transcriptomics is very scarce and does not allow to apprehend the data: "Spatial analysis revealed a correlation between the MAPK/ERK signaling pathway and type II IFN secretion, production, signaling (Figure 4D and E).' How p-Erk signaling in the spatial data in the fig.4D was defined? How features such as IFN secretion or signaling which are transcription in dependent are evaluated by spatial analysis? If 12 GB samples were used, was there any quantification? What does it mean type I and II IFN signatures? How they were constructed? Please provide more detailed explanation. Color code for TAMs and CD8/CD4 cells in Fig.4G makes it too difficult to distinguish. All IFN types seem to have exactly same expression pattern. These results should be validated with different tools (MERSFISH, IHC for IFN).
6. Suppl. Fig.S4. Why Sox2 positive cells (tum or cells) seem to be the ones expressing IRF9? One would expect some expression in TAMs?
7. Fig.6. The conclusion that the occurrence of high p-ERK and HLA-DR+ cells indicate modulation of antigen presenting processes is too far reaching. HLA-DR staining visualizes myeloid cells and does not indicate antigen presenting processes. I am not convinced SOX2+/ p-ERK+/ HLA-DR+ cells are tumor cells presenting antigen and it is not clear in which areas the staining was performed. Presentation of scRNAseq data is not clear and clusters of tumor cells should be better marked to visualize differences after treatment. We do not know if there is reduced expression of HLA after BRAFi/MEKi or HLA expressing cells are eliminated.
8. A number of samples used for the analysis in slice culture would be useful.

Minor comments:
Fig.2 A. The upper description of overexpressing cells is shifted to the left.

Reviewer #2

(Remarks to the Author)
This manuscript by Kim et al, reports data related to the impact of the MAPK/ERK signaling pathway in gliomas and the modulation of interferon responses, T cells' recruitment, microglia phenotype and the efficacy of immune checkpoint blockade. The work is topical and rigorous. The data is comprehensive and for the most part the conclusions are in line with the experimental results presented. Please find enclosed below several comments aiming at clarifying and enhancing the work reported.

1- In the data reported in Fig 2, it would be valuable if the authors investigated in mid-point and symptomatic tumors the myeloid immune compartment, which is highly prevalent in gliomas and is a major contributor to immunosuppression.

2- In Figure 3, the authors should include a diagram of the experimental design, indicating the treatment initiation time and treatment schedule for the survival data shown.

3- It would be required that the authors show some function assays related to interferon signaling on glioblastoma, both in their mouse models and human samples and how these are related to MAPK/ERK signaling pathway.

4- The same applies to the need to provide functional assays related to antigen presentation in order to provide functional validation for the conclusions put forward by the authors.

5- The data presented in Figure 7 also needs some functional validation in relation to the impact of these interactions in the biology they are reporting in this manuscript. It is felt that this is a correlative and secondary finding that does not contribute significantly to the primary and overarching message conveyed by this manuscript.

Reviewer #3

(Remarks to the Author)
What are the noteworthy results?
How MAPK signaling in tumor cells shapes the microenvironment is an important question, since GB can have upregulated MAPK signaling. Kim et al attempt to address if there is a link between MAPK activation and anti-tumor immunity in glioblastoma (GB), in particular CD8+ T cell mediated immune editing. A previous paper from this group identified MAPK signaling as an enriched pathway in CD8 T-cell depleted mice. Here, Kim et al propose to provide the mechanisms underlying MAPK/ERK pathway signaling and susceptibility to immune checkpoint blockade. They use CRISPR-mediated

genome editing to knock-out kinases and find that MAPK are the top enriched kinases in GL261-cells in CD8 KO mice vs wt mice. They provide preliminary evidence suggesting that MAPK signaling is functionally involved in immune editing and is linked to a CD8-mediated anti-tumor immune response. They attempt to prove that MAPK activation enhances glioma susceptibility to anti-PD-1 therapy and promote durable anti-tumor activity.
While each point is of importance, there is little novelty in this work. Their previous data already suggested that CD8+ T cells are employed to immune edit glioma cells (in a PDGF Pten-/- murine glioma model). In addition, there are other groups that have shown the same association. Moreover the data generated have severe technical limitations which are further described below, and which further limit the enthusiasm for this work
They fail to cite important literature of relevance to their field of research. For example, it is well known that in pediatric glioma a PXA-like tumor subtype is enriched for CD8 T cells and BRAF mutations, already suggesting that increased MAPK signaling (BRAF mutations) in high-grade gliomas associated with increased immunogenicity (PMCID: PMC5956280).
Kim et al use CD8 KO mice in this manuscript, which is not a conventional approach in immunology work (see also comment below). CD8 KO mice have CD4 T cells which could compensate for the lack of CD8 T cells. As shown in an older study using CD8/CD4 double knockout mice, alternative T cells could compensate for the lack of CD8/CD4 T cells (PMID: 8500525). The various T cell populations documented in glioma need to be quantified in the CD8 KO mice bearing murine glioma, to gain mechanistic insights and provide support for their claims that high MAPK signaling is immune edited.
Will the work be of significance to the field and related fields? How does it compare to the established literature? If the work is not original, please provide relevant references.
The work provides very incremental progress from a previous study published in 2020 by this group (PMCID: PMC7442699).

Does the work support the conclusions and claims, or is additional evidence needed?
The work only partially supports the conclusions and claims. Yes, additional evidence is needed as mentioned below. The authors's claim that they provide a mechanistic link of MAPK pathway activation (p-ERK) and immune evasion is not supported by their data. How do they propose the data could benefit patients? The clinical translation should be discussed in more detail.

Are there any flaws in the data analysis, interpretation and conclusions? Do these prohibit publication or require revision?
Is the methodology sound? Does the work meet the expected standards in your field?
The methodology and approach is a major weakness of this manuscript. Validation experiments are essentially missing from the entire manuscript. Most experiments were performed in a single cell line (AM38) or in just one murine glioma model (GL261). Of particular concern are their choice of murine and human models. GL-261 is a poor model for GB and fails to recapitulate the genetics and phenotypes of the human disease. GL-261 is hypermutated and is partially immunogenic. This casts doubts on the results from the CRISPR screen, which was just performed in this GL261 model. They need to show the CRISPR screen and MAPK over expression experiments in at least two murine glioma models side-by-side, and the models need to be more relevant to the disease.
There are also dramatic inconsistencies with the existing literature, which are not reconciled in the discussion. As an example, GL261 is a hypermutated cell line sensitive to immune checkpoint blockade (PMCID: PMC5724383). However, Kim et al show a minor response to PD-1 immunotherapy. In addition, AM38 is a serum-grown cell line for a BRAF V600E mutant GB. Why did they not use additional GB cell lines with MAPK pathway activation? Experiments in a single cell line are uninformative. Additional biological replicates are needed to provide credibility to the statements made by this group.
Is there enough detail provided in the methods for the work to be reproduced?
Each experiment needs to be described in much greater detail to be informative for the readership. As they stand right now, the methods are not described in sufficient detail to be recapitulated elsewhere.
For example, the lentivirus library is not described at al (is this a pan-kinase library?). Information about drug concentration and treatment schedules is missing.
Additional points that need to be addressed.
What are the effects of MAPK activation on IFN signaling, T cells, and responsiveness to immunotherapy in recurrent tumors? Since this type of tumor is usually resistant to BRAFi+MEKi treatment, it is not clear whether tumors in this study are responsive to targeted therapy or resistant since reactivation of the MAPK pathway in recurrent tumors is one of the resistance mechanisms.
Since MAPK activation improved the efficacy of anti-CTLA-4 immune checkpoint blockade in addition to anti-PD-1 treatment, authors should study the effect of combining aPD-1 and aCTLA-4 blockade therapy using Mek1/2-overexpressed mice?

The authors found downregulation of IFN signatures and HLA molecules in BRAFi/MEKi-treated samples using a patient-derived slice culture model compared to TMZ treatment. However, it is unclear how BRAFi+MEKi treatment affects MAPK signaling and IFN and HLA signatures during 7 days of treatment. A time-dependent analysis of these processes is required to determine the expression level of IFN signatures and HLA molecules at different time points during treatment and potentially link them to each otehr.
Minor points
The figure legend for Supplementary Figure 5B mentioned Type I interferon signaling, but the figure and text indicated Type II Interferon signaling. Please revise.
Representative images for cell proliferation assay are missing in Supplementary Figure 6B.
Representative images for IF analysis are missing in Supplementary Figures 4 and 9.
The figure legend for Supplementary Figure 8 is not well described.

Why did the authors focus on the expression of MHC II-related transcripts after ERK2 KO in AM38 cells (Supplementary Figure 10)? Does ERK2 KO also downregulate MHC I-related gene transcripts? Since “antigen processing MHC I” is shown

in the AM38-NTC from GSEA analysis (Figure 5B).
It is interesting to see that BRAFi/MEKi treatment significantly decreased the crosstalk between tumor cells and microglia (I assume by comparing with TMZ treatment), and modulated microglia/TAM phenotypes by increasing anti-inflammatory monocytes.
Given that IFN signaling is associated with cytotoxicity, is there a correlation between MAPK activation and inflammation? Do Mek1/2-overexpressed samples secrete more pro-inflammatory cytokines/chemokines in the tumor microenvironment? In addition, since TMZ treatment is used for comparison, how does the cell-cell communication network look in the untreated (control-treated) tumor sample?
In Figure 7A, BRAFi+MEKi treatment seems to kill more NPC-like tumor cells compared to TMZ treatment, it would be good to explain this observation.

Reviewer #4

(Remarks to the Author)
Kim et al reported that MAPK/ERK activation in tumor cells increased interferon responses and T cell activity, sensitizing mouse GBM tumors to immune checkpoint blockade. Using a kinome-wide CRIPSR screen, the author identified that MAPK/ERK is a regulator of tumor resistance to anti-PD1 treatment. They showed that overexpression of MARK/ERK sensitizes GL261 tumors to checkpoint inhibitors. Elevated p-ERK in glioma cells was associated with increased interferon responses, antigen presentation and T cell infiltration in GBO. Overall this is an interesting manuscript. However, some findings are descriptive. The authors may address the following concerns to improve their study:
Major
1) There is a major concern regarding the rationale of the CRISPR screen. GL261 glioma cells lentivirally transduced with a sgRNA library were implanted intracranially into wildtype and Cd8 KO mice, and the enriched kinase CRISPR was identified in the Cd8 KO mice. This screen will identify not only the kinases that regulate tumor cell response to T cells, but also the kinases that directly contribute to tumor cell proliferation. Therefore, MAPK/ERK could be identified, as it induces tumor cell propagation. In addition, GL261 is a suboptimal model for studying GBM resistance to checkpoint inhibitors and for the screen, as it does not recapitulate the clinical responses.
2) The potential translational impact is relatively moderate. Will the authors propose to use activation of MARK/ERK as a strategy to treat GBM? This apparently will increase tumor growth.
3) No mechanism study to investigate how MARK/ERK activation induces interferon responses and stimulates T cell activation.
4) There is no solid data supporting the conclusion in the mechanism study. Most of results are based on association studies. Particularly, there is no experimental data supporting the microglial phenotypes: only a prediction based on cell-to-cell communication analysis of slice cultivated human GB samples treated with temozolomide.
Minor
Fig. 1. A snapshot of whole kinome data would be helpful to see the global changes. Any positive or negative control?
Fig. 2. The authors may also show the effects of expression of constitutively active MEK/ERK mutants in addition to overexpression of the kinase.
Fig. 2D-G. No tumor growth data shown. Will MAP/ERK activation increase tumor growth?
Fig. 3. Confirmation with additional glioma models is suggested.
Fig. 6. Co-culture of tumor cells with T cells to test their antigen presenting functions is suggested.

Version 1:

Reviewer comments:

Reviewer #1

(Remarks to the Author)
The authors addressed all comments of this referee and performed several experiments and recalculations to provide answers. Some of the results acquired to respond to reviewer's quarries which did not support the author's interpretation have been removed. The requested additional information along with a more precise explanation of the results is provided in the revised version. The data are now comprehensive and for the most part the conclusions are in line with the experimental results presented. The new data clarified and enhanced the work reported. Originally indetified minor flaws in the data analysis, interpretation and conclusions have been corrected.
The revised version is greatly improved.Regarding comments of other reviewers, I agree with researchers that the data from the ex- vivo slice culture model and an experimental perturbation of MAPK signaling with BRAF inhibition investigated the effect of this intervention on the cross-talk between tumor cells and TAM and provide a causative link. The findings of the manuscript indeed established a causal evidence that MAPK drives glioma immunogenicity and response to immunotherapy and explain the results of several clinical trial and human cohort analyses where patients with MAPK exhibit durable responses and favorable survival following immune checkpoint blockade. The results are novel, interesting and important for the field.

Reviewer #2

(Remarks to the Author)
The authors have extensively revised their manuscript, preforming additional experimentation to answer all the reviewers'

comments. The data is rigorous, and the conclusions are in line with the data reported. I have not additional concerns.

Reviewer #3

(Remarks to the Author)
We thank the authors for their thorough revision. The stratification of GBM patients likely to respond to immune checkpoint inhibition is a highly relevant topic, and the translational value of this work is significant. In this study, they demonstrate that MAPK pathway genes, known to upregulate pERK upon activation, are also regulators of susceptibility to CD8 T cell activity and anti-PD-1 treatment. These data suggest that cells exhibiting high MAPK pathway activation are more effectively eliminated by T cell-mediated killing.
While the authors' revision is extensive and some of the data are compelling—such as the potential for MAPK/ERK signaling to predict responses to various forms of GBM immunotherapy—there remain concerns regarding methodology and the limited integration with existing literature, which may lead to contradictions with published data.
1. The overexpression of MEK genes via lentivirus in the hypermutated GL-261 model may itself induce a T cell response, likely activating T cells and sensitizing them to PD-1 immunotherapy due to artificial overexpression of antigens. This limitation should be discussed in the manuscript.
2. The data for BRAFi+MEKi presented in Figures 5 and 6 raise significant concerns and do not adequately support the claim of a mechanistic link between p-ERK and IFN responses in GBM. The reliance on spatial data from a single patient in Figure 5, while replicative, lacks proper controls for BRAFi+MEKi (only TMZ as a comparator). Furthermore, the assertion that inhibiting ERK signaling impairs the induction of IFN response genes conflicts sharply with a recent study by Xing et al. This study demonstrated, through multiple high-fidelity model systems and patient-derived materials, that BRAFi+MEKi treatment actually induces IFN response genes. The authors must cite this study in the introduction and thoroughly discuss the discrepancy within the manuscript. The evidence presented by PMCID: PMC12208339 indicates that BRAFi+MEKi increases MHC class II gene expression in microglia and tumor cells, in a syngeneic mouse model that is not particularly sensitive to immune checkpoint blockade, suggesting an opposite effect to what is reported here. Additionally, PMCID: PMC12208339 indicated that MHC class II expression upregulation does not correlate with ICI response, pointing to other influencing factors, such as immunosuppressive cytokines, that may dampen the ICI response.
3. In Figure 6, while a control arm is included in the multiplex analyses, only a single time point is assessed. As suggested in the initial review, conducting a time-course study and including additional models (e.g., AM38 or other pERK high models or MAPK signaling mutant models) would be beneficial in investigating the temporal effects of BRAFi+MEKi on IFN signature expression. Addressing these discrepancies and incorporating a rigorous discussion of contradictive results is critical.
4. The authors do touch on some limitations in their discussion, such as the lack of an adequate immune system in their human slice cultures; however, other limitations should be candidly acknowledged.
Additional complexities and discrepancies with existing literature must also be addressed prior to acceptance of this article. As mentioned above, a recent paper (Xing et al, PMCID: PMC12208339) showed that adult BRAF-mutant/fusion GBM exhibits significantly higher levels of PD-L1 than BRAF wild-type GBM, validated by two orthogonal methods. Given the relevance of this patient population to the current study, it should be incorporated into the introduction.
The authors should also discuss the implications of robust PD-L1 expression in BRAF-mutant GBM, which may even surpass that of pERK. A comparative discussion of how PD-L1 stands as a predictive biomarker for PD-1/CTLA-4 immune checkpoint inhibition versus pERK is warranted. Xing et al. found that BRAFi+MEKi treatment amplifies PD-L1 expression and induces IFN expression in CD4 T cells, supporting concurrent therapy with anti-PD-1 and anti-CTLA-4. Notably, in mouse glioma models, the combination of these four drugs resulted in greater survival benefits compared to individual drug combinations, even in BRAFi+MEKi resistant glioma. As previously noted, in therapy-resistant models, there is consistent upregulation of IFN and HLA genes with BRAFi+MEKi, fundamentally contradicting findings presented here. Moreover, this upregulation does not yield survival benefits across all models, suggesting that factors beyond MHC class II-mediated antigen presentation must be considered to predict beneficial responses to ICI.
The findings presented by the authors and those in PMCID: PMC12208339 are sharply contradictory. Xing et al. clearly demonstrated the upregulation of IFN signatures in multiple systems following BRAFi+MEKi treatment, with these signature expressions being time-dependent. They further established that either concurrent treatment with BRAFi+MEKi and ICI or sequencing ICI prior to BRAFi+MEKi treatment yields better outcomes than either treatment alone (or administering ICI after BRAFi+MEKi).
The publication of this manuscript could potentially create confusion regarding the impact of these inhibitors on IFN signatures and complicate future research and clinical trials.
The authors may choose from several options to mitigate these issues: 1. Remove the data regarding BRAFi+MEKi since they are premature and need additional controls and experimentation; 2. Add additional controls and experimentation (such as more relevant models and investigate additional time points); and 3. Significantly revise statements concerning these data, clearly acknowledging all study limitations (e.g., performed only in slice cultures lacking comprehensive immune components, absence of temporal resolution, etc.) and discuss their findings in the context of established literature, allowing for acknowledgment of differences potentially due to technical limitations. For instance, variations in mouse model selection likely influence results, as does the timing of measurements (past studies by the authors indicate that elevated p-ERK levels are only predictive shortly before PD-1 treatment and exhibit longitudinal fluctuations).
The authors previously provided convincing evidence that p-ERK1/2 is a predictive marker for response to anti-PD-1 immunotherapy in recurrent glioblastoma. Building on this foundation, the current paper supports the idea of screening recurrent GBM for p-ERK levels and treating patients exhibiting high p-ERK levels with anti-PD-1. This approach remains encouraging.
Furthermore, while they previously associated increased p-ERK levels with greater microglia frequency and enhanced MHC class II gene expression on these cells correlating with increased responses to PD-1, they should also explore alternative mechanisms through which MAPK pathway activation could affect microglial behavior (such as the secretion of microglia-recruiting cytokines), as demonstrated by Kocher et al. (PMCID: PMC11147834) in the BT40 model, which, despite not

being GBM, exhibits high-grade glioma features.

Reviewer #4

(Remarks to the Author)
The manuscript has been improved in this revision. However, the authors have not addressed most of the key concerns raised previously.
1) As commented by both Reviewers #3 and #4, GL261 is not a good model studying glioma immunology. The key in vivo results in Figures 2 and 3 need to be validated using additional animal models.
2) The overall findings are relatively not novel. The advance over the previous literature (from others and the same group) is incremental.
3) Mechanism study is relatively descriptive. No experimental data have been provided to define how MAPK/ERK activation induces IFN response.
4) The translational impact is relatively weak. It is still not clear whether tumors in this study are responsive to targeted therapy or resistant, since reactivation of the MAPK pathway in recurrent tumors is one of the resistance mechanisms. It is confusing that this work suggests potential combination of MAPK/ERK pathway activation with immune checkpoint inhibitors to counterbalance the pathway's tumor-promoting effects while enhancing the immune response.

Version 2:

Reviewer comments:

Reviewer #1

(Remarks to the Author)
The authors addressed all concerns of this referee.
Regarding the comments of other reviewers:
Reviewer 3:
1. I agree the author's comments that they took sufficient care to control for potential sensitizing to PD-1 immunotherapy due to artificial overexpression of antigen. They used proper controls (identical lentiviral manipulation for Mek1/2 OE and control vector infected GL261 tumors), so differences in anti-PD-1 responsiveness reflect MEK pathway activation rather than vector-mediated immunogenicity.
The experiment with rechallenge with implanted wildtype GL261 cells long time after original implantation and rejection of those tumors demonstrates the presence of long-term immune memory against GL261 antigens, not lentiviral components. Many data show that GL261 do not show intrinsic responsiveness to PD-1 blockade (Pilanc et al Front Oncol 2021, Dmello et al. Nat. Comm. 2023; Kim et al, Neuro-Oncol. 2024; Ellert-Miklaszewska et al. J Exp Clin Can Res 2025) unless subjected to TME immunomodulation. I agree that other murine glioma models cited by the reviewer (2341, M34, and RCAS models) are not much better than the ones employed in the study, as they have different immunogenic features—GFP, luciferase or avian lentiviral elements, Cre delivery, and vector–associated immune activation. I agree that increased T cell infiltration is unlikely to be related to antigenicity of the transgene itself.
2. The authors adequately responded to the reviewer concerns by explaining that their interpretation about the association of high p-ERK and IFN responses was based not on one patient (used to illustrate the result), but on the a larger cohort (n = 26 IDH-wildtype, BRAF-mutant GBM dataset).
They also included a non-treated control group in the Fig. 5. Their interpretation holds as the BRAFi- treated group compared with the untreated control and the TMZ-treated groups, exhibits reduced type I and type II IFN response signatures.
The authors discuss the results of Xing et al. (PMCID: PMC12208339) which are not fully comparable to this study and moderated their statements regarding MAPK-antigen presentation.
3. I am convinced that for human data and associations with therapy outcomes, time course is not necessary and in most cases not available. I do not think that time course experiments would be very informative and necessary. The expectation of using multiple models (e.g., AM38 or other pERK high models or MAPK and reproducing studies with those is a bit unrealistic considering time and money and would not bring new data. The employed models (CRISPR-based KO of ERK1 or ERK2 to are selective, sufficient to demonstrate the tested associations. Using other MAPK models would complicate the issue and data interpretation, as they are more complex, with additional signaling networks and it would be difficult to attribute action only to ERK. I am convinced that the authors made big efforts to provide convincing data that support their claim.
4. The issue of limitations of the applied slice culture systems was well addressed, the authors are candid about limitations and in the revised manuscript they acknowledge more potential limitations.
5. I do have a strong impression that the reviewer is very influenced byaa the study published by Xing et al. (PMCID: PMC12208339) which actually is in contrast to some other reports. The authors pointed out what tehir interpartation is a nd I agree with that opinion. It is clear that the present study is more comprehensive regarding IFN responses after ERK manipulation and the data presented fits together. They used several mouse tumor models (GL261, CT-2A, QPP7) and extensively crosschecked their results with human data. I disagree with the recommendation that some data (analysis of RAFi+MEKi) should be removed since they are premature. These data add an important information and are of value to community.

Reviewer 4

GL261 is a most frequently used glioma model and as such allow to interpret the data in well known context and make them more comparative with already collected data. The other models have their own limitations. Despite being more immunogenic than some GBMs, GL261 recapitulates many features of GBM biology and immunology. The authors reproduced important findings in other mouse tumor models (GL261, CT-2A, QPP7), so I do believe their claims are well founded and not exaggerated.
The findings are not incremental, they are important for the field, even if not all mechanisms have been reported and mechanistic insights are not full revealed. The reviewer expectations are sound but ignoring some of the practical difficulties involved in achieving such high goals. The study a well-focused and brings insigths important for the community.
The value of the study should not be based solely on clinical relevance or translational aspects but should be defined by the clarity, novelty and good experimental standards which are all high in this case.

I think the authors addressed fully, both experimentally and in discussion, all important concerns of the reviewers.

Reviewer #2

(Remarks to the Author)
I remain enthusiastic about this manuscript; the authors have revised their manuscript extensively and have addressed all my comments. The work is interesting and it will add valuable information to this filed. In my opinion, some of the issues raised by the other reviewers could be addressed by including a section highlighting the limitation of this study. The data are rigorous and the conclusions are in line with the results presented.

Open Access This Peer Review File is licensed under a Creative Commons Attribution 4.0 International License, which permits use, sharing, adaptation, distribution and reproduction in any medium or format, as long as you give appropriate credit to the original author(s) and the source, provide a link to the Creative Commons license, and indicate if changes were made.
In cases where reviewers are anonymous, credit should be given to 'Anonymous Referee' and the source.
The images or other third party material in this Peer Review File are included in the article’s Creative Commons license, unless indicated otherwise in a credit line to the material. If material is not included in the article’s Creative Commons license and your intended use is not permitted by statutory regulation or exceeds the permitted use, you will need to obtain permission directly from the copyright holder.
To view a copy of this license, visit https://creativecommons.org/licenses/by/4.0/

REVIEWER COMMENTS

Reviewer #1 (Remarks to the Author):

The rationale for this study is based on the observation that MAPK/ERK signaling is associated with overall survival following immune checkpoint blockade (with anti-PD-1 or anti-CTLA-4) in recurrent glioblastomas (GBM). The authors capitalize on the results of the previous study in which they performed the kinome-wide CRISPR/Cas9 screening in murine GL261 gliomas and identified Chek2 as main regulator in CD8KO cells (doi: 10.1038/s41467-023-36878-2.).

In the present study the authors applied similar CRISPR/Cas9 screening to murine 261 glioma cells to identify key regulators in CD8 KO and anti-PD-1 treated mice. This time the Gene ontology (GO) analysis revealed Map2k2 (encoding Mek2) and Araf from the MAPK pathway as the most significantly enriched among all kinase pathways in CD8 KO, and Araf, Map2k1 (encoding Mek1) and Erk2 in anti-PD-1 treated mice. Moreover, Mek1/2 Erkhigh tumors respond well to CTLA4 blockade. Knockout of Erk1 or Erk2 in murine QPP7 gliomas, sensitive to anti-PD1, produced same responses as in control gliomas.

The validation part encompasses overexpression of Mek1 or Mek2, implanting cells and seeking effects on survival. They studied CD8+ T cell responses and survival to validate selected genes. Experimentally-induced ERK phosphorylation in glioma cells enhanced survival after anti-PD1 treatment, led to anti-tumoral immunity upon re-challenge and increased infiltration of memory T cell in long-term survivors. Spatial transcriptomics and scRNA-seq analysis revealed the modulation of interferon responses by the MAPK/ERK pathway in BRAFV600E human GBM cells with ERK1/2 knockout and in slice cultures of human BRAFV600E GBM tissue. Ex vivo a BRAFV600E mutant GBM tumor slices treated with inhibitors (BRAFi/MEKi) demonstrated links between tumor cell immunogenicity. They claimed that BRAFi/MEKi treatment disrupted the interaction between tumor cells and tumor-associated macrophages/microglia in slice cultures from BRAFV600E mutant GBM.

Altogether, the results are intriguing, against what to be expected about roles of IFNs in antitumor responses. The results are significant to the field of tumor immunology. The authors used a plethora of methods to verify their conclusions. The study demonstrates the particular mechanism underpinning the resistance of GBM immune checkpoint blockade using murine glioma cells overexpressing Mek1/2 with high ERK activation, which extends to BRAFV600E mutant GBM cells and ex vivo slices. Potential mechanisms have been verified. The data are novel and the findings are in most aspects solid and well presented. Some parts of the manuscript may benefit from more detailed explanation of some results. Technically, the proper state of the art methods have been applied. A number of animals in experiments is sufficient and proper statistical analyses were employed when necessary. In some aspects the results and their interpretation is a

bit confusing. The methodology sound and this work meets the expected standards in your field?

There are enough details provided in the methods to reproduce the work. There are minor shortcomings that could be improved by a proper revision or re-writing.

Response: Thank you for your insightful comments and positive assessment of our study. We appreciate your recognition of the novel contributions our work makes to the field of tumor immunology, particularly in elucidating the role of the MAPK/ERK pathway in modulating immune responses in GBM. We agree that some parts of the manuscript would benefit from additional clarification, especially regarding the interpretation of certain results. In response, we have revised the Results and Discussion sections to better highlight the rationale behind our mechanistic investigations and to clearly delineate the conclusions drawn from each experimental approach. Specifically, we have elaborated on the link between MAPK/ERK activation and interferon responses, addressing the counterintuitive finding that enhanced MAPK signaling, typically associated with oncogenesis, can also potentiate antitumor immunity under certain contexts.

Major comments:

Comment 1: Fig.2. The authors overexpressed Mek1 and 2 to achieve high Erk activation and such enhancement of MAPK/ERK signaling increased CD19 fraction (consistent with higher $CD8^{+}$ cell infiltration) and improved survival after anti-PD1 treatment.

Response: We thank the reviewer for this insightful observation. The increase in the $CD19^{+}$ fraction following MAPK/ERK activation is indeed a compelling finding, and we have taken note of this with great interest. The role of B cells in GBM, particularly in the context of immunotherapy, has gained increasing recognition in recent years (Wang et al., JCI, 2024; Hou et al., Frontier in Immunol., 2024; Gao et al., Cancer Cell, 2025).

Comment 2: The appearance of Mek1/2 Erk^{low} tumors is surprising and confusing, and it could be may be reasons that they respond differently to therapy. Basal p-Erk in suddenly high in those cells in contrast to fig.2A. There are some inconsistencies in detecting Erk/pErk. There is discrepancy in basal p-Erk levels between different panels. Fig.2. A. F- The part A shows low level of p-Erk in control, but it is much higher in the part F, where Mek1/2Erk low cells are presented. Supplementary S2. Why Western blot shows a single Erk1 or Erk2 band while there should be two bands?

Response: We appreciate the reviewer for this careful evaluation of our manuscript. The apparent discrepancy in basal p-Erk levels, as noted between Figure 1A and 1B of this document (Manuscript Figure 2A and 2F), primarily results from differences in blot exposure times. It is crucial to highlight that although the band density appears different,

this does not necessarily indicate biological variance. The comparison of these levels should be limited to conditions/clones tested within the same blot (Figure 1A and B of this document). To clarify, Erk1 and Erk2 are indeed distinct isoforms, commonly referred to as p44 and p42, respectively. In Supplementary Figure 4, QPP7 cells with targeted knockouts of *Erk1* (*Erk1* KO) show an absence of the p44 band, confirming the effective knockout of *Erk1*. Similarly, QPP7 cells with *Erk2* knockout (*Erk2* KO) show an absence of the p42 band, which confirms the knockout of *Erk2* (Figure 1C of this document).

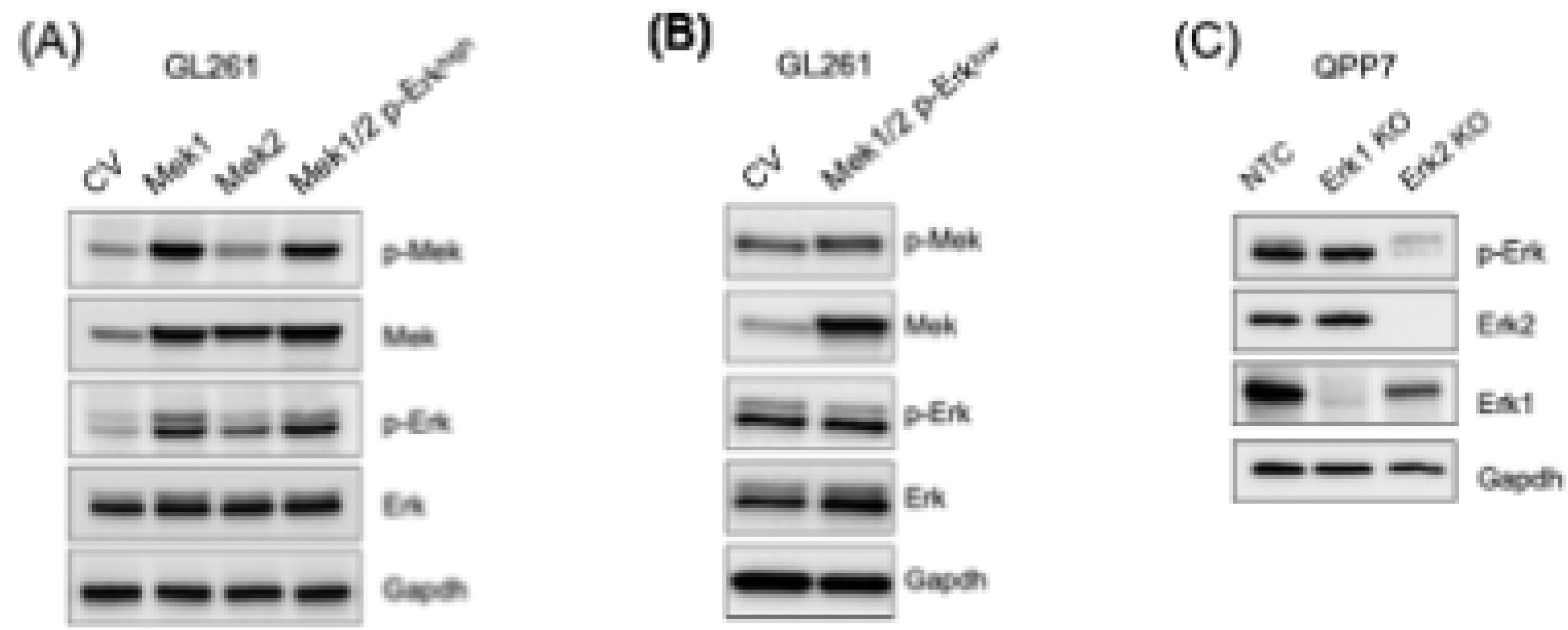

Figure1. Western blot analysis of GL261 cells double-overexpressing Mek1/Mek2 with (A) p-Erk^{high} or (B) p-Erk^{low}. (C) Western blot analysis of QPP7 cells with Erk1 or Erk2 knockout.

Comment 3: Fig.3. They wrote: “In the GL261 mouse glioma model, resistant to both conventional and Fc-enhanced aCTLA-4, activation of the MAPK signaling led to improved efficacy of Fc-enhanced aCTLA-4 therapy”. But the data on the figure shows the significant positive effect of the Fc-enhanced aCTLA-4. Please be precise in the description of the results.

Response: We thank the reviewer for pointing this out. In our recent publication (Kim et al., Neuro-Oncology 2024), we extensively reported on the efficacy of Fc-enhanced aCTLA-4 (FcE-aCTLA-4) therapy and its potential for combination treatments in several preclinical glioma models. Specifically, our study explored the efficacy of FcE-CTLA-4, noting a modest improvement in the GL261 glioma model. Therefore, we tested combination therapies (with DOX and LIPU) in the GL261 model. The GL261 overexpression model we established, using lentiviral vectors to create clones, may potentially induce host immune responses. Unlike the parental GL261 cells, the GL261-CV (control vector) clones showed an enhanced response to FcE-aCTLA-4 therapy. Most critically, as detailed in Manuscript Figure 3, it is important to note that high p-ERK GL261 tumors responded completely to FcE-aCTLA-4 therapy, with all treated mice (10/10) achieving long-term survival. This finding underscores the potential of MAPK signaling activation to improve the efficacy of immune checkpoint blockade in this otherwise resistant glioma cell line/clone. We have also revised the Results section corresponding to Manuscript Figure 3 more clearly highlight this finding and its implications.

Comment 4: Fig.4. Regarding the selection of tumor areas with low or high p-Erk staining. The authors state: “Tumors with high p-ERK exhibited a significantly higher density of SOX2+p-ERK+IRF9+ tumor cells compared to low p-ERK tumors” and quantification shows a number of positive cells per mm2. But what about tumor cell density in those areas? IHC staining in the A panel C seems to show low cell density. High-resolution picture should be demonstrated to show co-localization of IRF9 and p-Erk, as it is, it is not convincing.

Response: We thank the reviewer for bringing up this important point. As the reviewer correctly noted, we observed that tumor regions with high p-ERK staining often exhibited higher overall cell density (Figure 2A and B of this document). When we normalized the SOX2^{+}p-ERK^{+}IRF9^{+} tumor cell counts to account for this variation in cellularity, the previously observed differences between high and low p-ERK tumors were no longer statistically significant (Figure 2C of this document). We will therefore removed these results from the revised manuscript and thank the reviewer for enhancing the rigor of our study. Nevertheless, the broad conclusions of the manuscript are not affected as we have IRF9-independent evidence that MAPK leads to activation of IFN responses in GBM, which we show in Manuscript Figure 4.

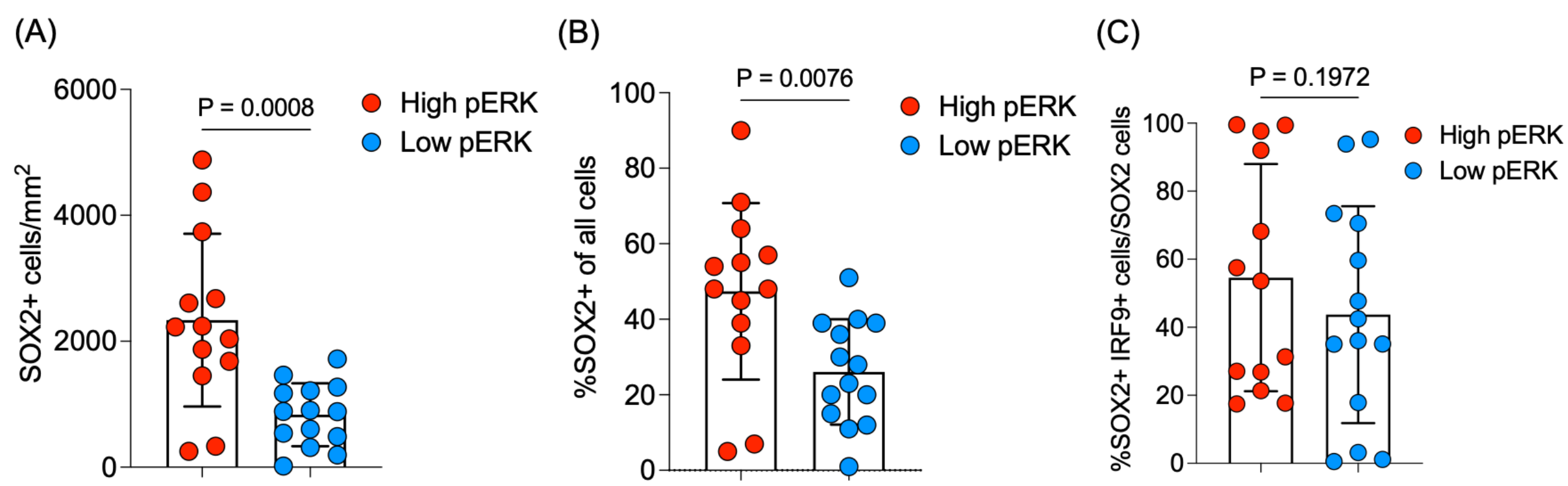

Figure 2. Multiplex immunofluorescence analysis comparing High p-ERK tumor and Low p-ERK tumor in human GBM patient samples. (A) Density of SOX2+ tumor cells. (B) Percentage of SOX2+ tumor cells. (C) Percentage of SOX2+IRF9+ tumor cells relative to the total number of SOX2+ cells.

Comment 5: Fig.4D-F. The description of findings from spatial transcriptomics is very scarce and does not allow to apprehend the data: “Spatial analysis revealed a correlation between the MAPK/ERK signaling pathway and type II IFN secretion, production, signaling (Figure 4D and E).’ How p-Erk signaling in the spatial data in the fig.4D was defined?

Response: We thank the reviewer for the comments. Our specific answers have now been incorporated into a revised manuscript, which are shown below:

To define the p-ERK signal we used the MSIGDB database with the pathways “BP.GO_POSITIVE_REGULATION_OF_ERK1_AND_ERK2_CASCADE” with the

SPATA2 functions: gene sets via SPATA2::getSignature(). The SPATA2 framework was recently published (Ritter et al., Nat Cancer, 2025; Kueckelhaus et al., Nat Comm., 2024; Ravi et al., Cancer Cell, 2022), including the various options for gene set enrichment analysis. To characterize not only the overall enrichment of the p-ERK pathway in the samples, we performed spatial autocorrelation to define if the ERK pathway is enriched in local niches or generally upregulated across the sample. We found that the ERK pathway is mainly enriched in spatial patterns.

Comment: How features such as IFN secretion or signaling which are transcription in dependent are evaluated by spatial analysis? If 12 GBM samples were used, was there any quantification? What does it mean type I and II IFN signatures? How they were constructed? Please provide more detailed explanation.

Response: We have revised the spatial data analysis and added new data to demonstrate a more comprehensive analysis of the IFN pathways. First of all, we added additional samples for a more comprehensive analysis (a total of n=26 *de novo* IDH-wt GBM). In the new cohort, we also added a spatial transcriptomic dataset of a $BRAF^{V600E}$ patient.

The enrichment analysis of IFN was also performed by gene-set enrichment analysis using the IFN signatures:

- *"BP.GO_RESPONSE_TO_INTERFERON_ALPHA"*
- *"BP.GO_RESPONSE_TO_INTERFERON_BETA"*
- *"BP.GO_RESPONSE_TO_INTERFERON_GAMMA"*

Comment 5-3: Color code for TAMs and CD8/CD4 cells in Fig.4G makes it too difficult to distinguish.

Response: We have kept the same color palette in our spatial analysis to allow readers to easily interpret and compare data across samples and studies. Specifically, the palette in Manuscript Figure 4 was chosen to match the cell annotation colors shown in Manuscript Figure 5. However, in response to the reviewer's suggestion—and given that Figures 4 and 5 in the manuscript are presented separately—we recognize that improved color contrast would enhance clarity. Therefore, we have updated the color codes in Manuscript Figure 4G to facilitate better visual distinction (Figure 3 of this document).

Comment: All IFN types seem to have exactly same expression pattern. These results should be validated with different tools (MERSFISH, IHC for IFN).

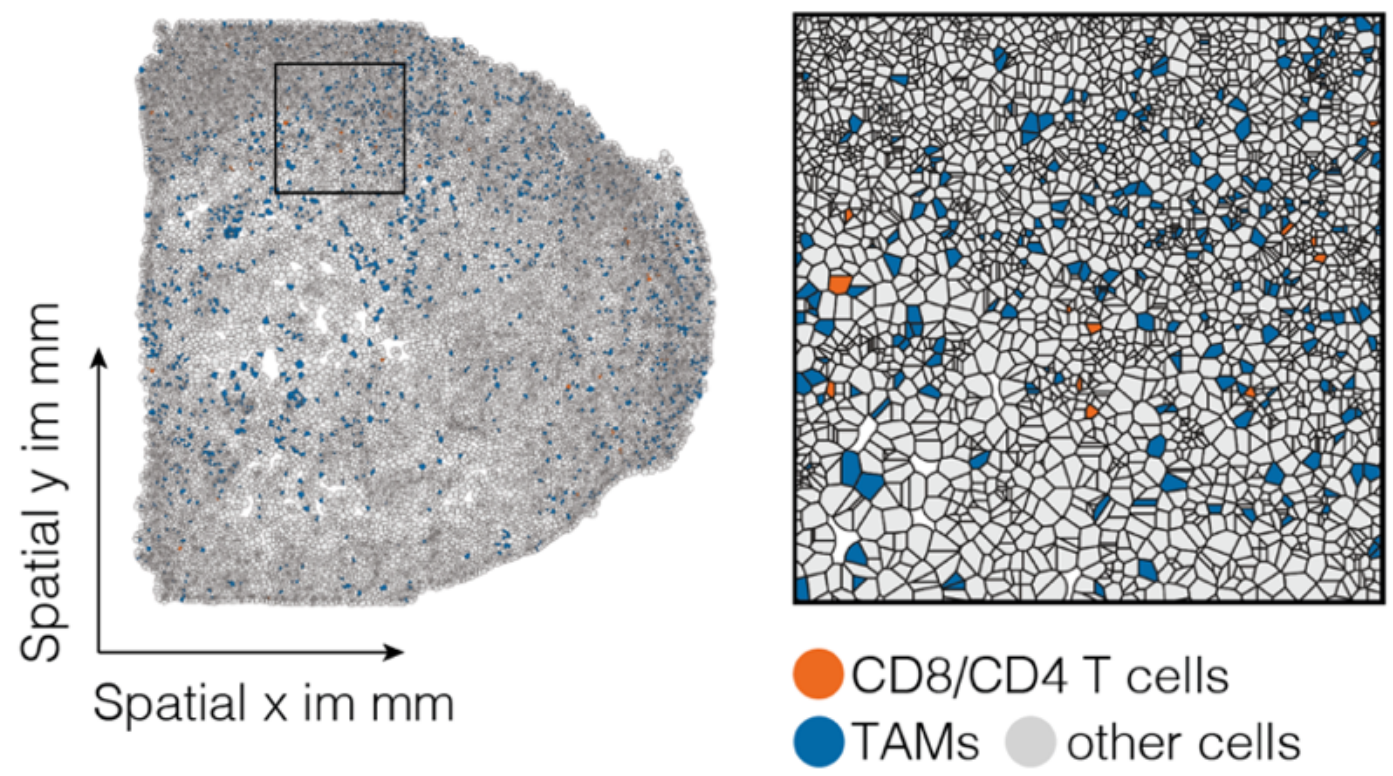

Figure 3. Spatial transcriptomic analysis with revised color palette for improved visual contrast and interpretability.

Response: As requested, we have now investigated the IFN signatures by MERFISH. Here is a description of our results: Because the ERK signal and the IFN type I (alpha) expressed appeared to be spatially correlated, we evaluated whether this reflected co-expression within the same cells or proximity of distinct cell types. To address this, we performed spatial neighborhood analysis of the MERFISH single-cell spatial transcriptomic data, computing the first-hop neighborhoods of tumor cells with high ERK signature across all subgroups (Figure 4G of the manuscript). Using single-cell spatial transcriptomics, we then quantified the probability of type I or II IFN (α/β or γ) pathway enrichment to be driven by direct cell–cell contacts within defined ecosystems. Type I IFN (in microglia) activation was markedly increased when tumor cells with high ERK signature were in close proximity to inflammatory microglia (Figure 4H of the manuscript). Further analysis revealed that oligodendrocyte progenitor cell (OPC)-like and neural progenitor cell (NPC)-like tumor subpopulations were the main contributors to this effect. In contrast, Type II IFN activation in myeloid cells was predominantly associated with neighboring astrocyte (AC)-like and mesenchymal (MES)-like tumor populations (Figure 4H of the manuscript).

Comment: Suppl. Fig.S4. Why Sox2-positive cells (tumor cells) seem to be the ones expressing IRF9? One would expect some expression in TAMs?

Response: We appreciate the reviewer for this critical question indeed. Our multiplex immunofluorescence results demonstrate that IRF9 expression is predominantly localized within tumor cells. We did not detect IRF9 expression in tumor-associated macrophages (TAMs) marked by CD163, or in microglia marked by P2RY12. Yet, given that we found the immunofluorescence results for IRF9 expression in p-ERK high tumors to be explained by differences in cell density, we have removed these results from the manuscript.

Comment: Fig.6. The conclusion that the occurrence of high p-ERK and HLA-DR+ cells indicate modulation of antigen-presenting processes is too far-reaching. HLA-DR staining

visualizes myeloid cells and does not indicate antigen presenting processes. I am not convinced SOX2+/ p-ERK+/ HLA-DR+ cells are tumor cells presenting antigen and it is not clear in which areas the staining was performed.

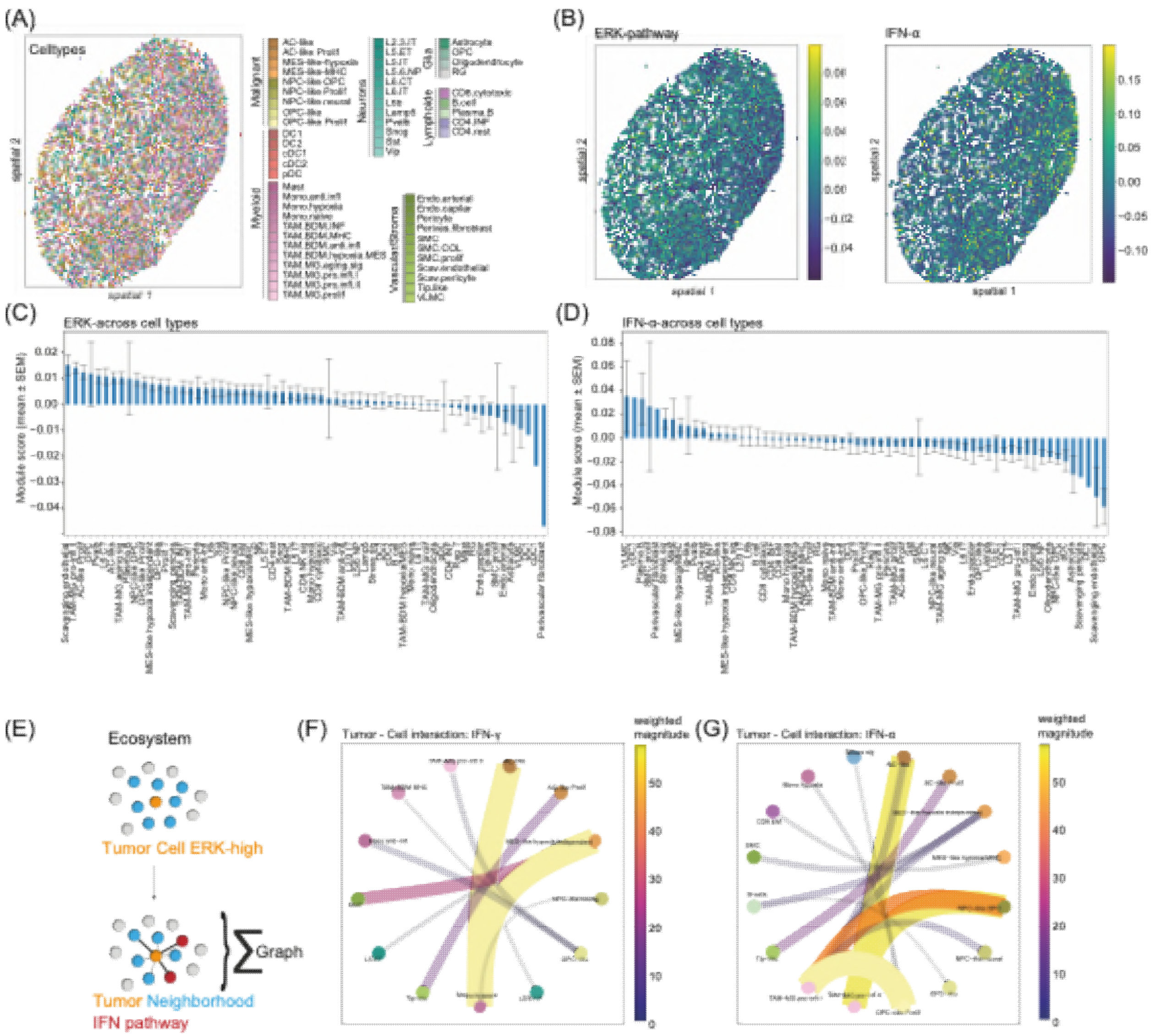

Figure 4. (a) Illustration of an example using tissue microarrays (n=6) of primary GBMs. Celltypes were annotated based on the GBMap. b) Cellular enrichment scores of ERK (left) and IFN-α pathway (right). Colors indicate the pathway enrichment score. (c) Mean ERK and IFN-α enrichment (d) across samples and celltypes. (e-g) Illustration of the network analysis. (f) Circular graph plot of the tumor-microenvironment proximity and IFN enrichment. Colors of the dots demonstrate the cell type, the edges represent the mean enrichment of the IFN pathway (weighted magnitude).

Response: We thank the reviewer for this important comment regarding the interpretation of HLA-DR staining. We agree that HLA-DR is conventionally considered a marker of myeloid cells; however, in our analysis, we specifically quantified HLA-DR expression in SOX2^{+} tumor cells, not in the myeloid compartment. We also agree with the reviewer that

one cannot conclude that the expression of HLA-DR in tumor cells implies that these can present antigens, and have been careful on the revised manuscript to avoid such claim. Notably, in the high p-ERK group of patients, we observed significantly increased HLA-DR expression in tumor cells, as determined by co-localization of SOX2 and HLA-DR in multiplex immunofluorescence staining, and supported this finding with transcriptomic analyses. Importantly, glioma cells are not professional antigen-presenting cells (APCs). Yet, it is well established that non-APCs can upregulate MHC class II molecules such as HLA-DR in response to IFN-γ stimulation. IFN-γ induces the activation of the transcription factor STAT1, which in turn activates CIITA, the master regulator of MHC class II gene expression, via IRF1 (Wright & Ting, Trends Immunol., 2006; Muhlethaler-Mottet et al., Immunity, 1998; Ting & Trowsdale, Cell, 2002; Reith et al., Nat Rev Immunol., 2005; Kobayashi & van den Elsen, Nat Rev Immunol., 2012). Thus, our observation of HLA-DR upregulation in tumor cells with high p-ERK may reflect a MAPK/ERK-driven enhancement of interferon responsiveness, potentially enabling glioma cells to engage with $CD4^+$ T cells in the tumor microenvironment. Furthermore, in addition to the density of $HLA\text{-}DR^+SOX2^+$ cells in the tumor, we further provide the percentage of $HLA\text{-}DR^+$ cells in $SOX2^+$ tumor cells to clarify this claim (Manuscript Figure 6).

We like to clarify that the stainings for this study were done in tumor-containing slides, and the analysis for quantification of multiplex immunofluorescence was done in regions that were outlined as contining viable tumor tissue, as determined by an expert neuropathologist.

Comment: Presentation of scRNAseq data is not clear and clusters of tumor cells should be better marked to visualize differences after treatment. We do not know if there is reduced expression of HLA after BRAFi/MEKi or HLA expressing cells are eliminated.

Response: Most of the single-cell RNA-seq dataset we analyzed is a publicly available resource (GBmap, Ruiz-Moreno et al., Neuro-Oncology, 2025; embedding was published and pre-established, which includes 109 samples of GBM patients) and was not generated from pre- and post-treatment samples. Instead, we performed ERK1/2 knockout in the human GBM cell line AM38 and identified the transcriptional signature associated with ERK loss. This ERK-loss gene signature was then projected onto the GBmap scRNA-seq dataset to explore its enrichment across different cell populations in GBM samples. We have clarified this point in the revised manuscript to avoid potential misunderstanding about the origin and interpretation of the scRNA-seq analysis.

Comment: A number of samples used for the analysis in slice culture would be useful.

Response: We thank for the insightful suggestions. We have added the information to the corresponding sections. The numbers are as follows: 1 patient with 6 slices in each condition (TMZ, Trametinib+Dabrafenib, Trametinib+Vemurafenib,

Trametinib+Encorafenib), 24 slices in total are quantified in BRAF-mutated GBM cell-autografted slice culture experiments).

Minor comments:

Comment: Fig.2 A. The upper description of overexpressing cells is shifted to the left.

Response: We thank the reviewer for the comment, and we have corrected Manuscript Figure 2A

Reviewer #2 (Remarks to the Author):

This manuscript by Kim et al, reports data related to the impact of the MAPK/ERK signaling pathway in gliomas and the modulation of interferon responses, T cells' recruitment, microglia phenotype and the efficacy of immune checkpoint blockade. The work is topical and rigorous. The data is comprehensive and for the most part the conclusions are in line with the experimental results presented. Please find enclosed below several comments aiming at clarifying and enhancing the work reported.

Comment: In the data reported in Fig 2, it would be valuable if the authors investigated in mid-point and symptomatic tumors the myeloid immune compartment, which is highly prevalent in gliomas and is a major contributor to immunosuppression.

Response: We appreciate the reviewer's insightful suggestion. We agree that investigating the myeloid immune compartment is of high relevance, given its abundance and immunosuppressive role in the GBM microenvironment. In our mid-point tumor analyses (mice were euthanized 21 days post intracranial implantation with GL261), we conducted flow cytometry to characterize the myeloid compartment. Interestingly, we observed that activation of the MAPK/ERK pathway appeared to shift both microglia and macrophages toward a more immunogenic phenotype. This was most clearly reflected by decreased expression of Arginase-1 and PD-L1 (Figure 5 of this document, Supplementary Figure 15 of the manuscript). Together, these data indicate that ERK activation may contribute to remodeling the myeloid landscape in a way that supports anti-tumor immunity.

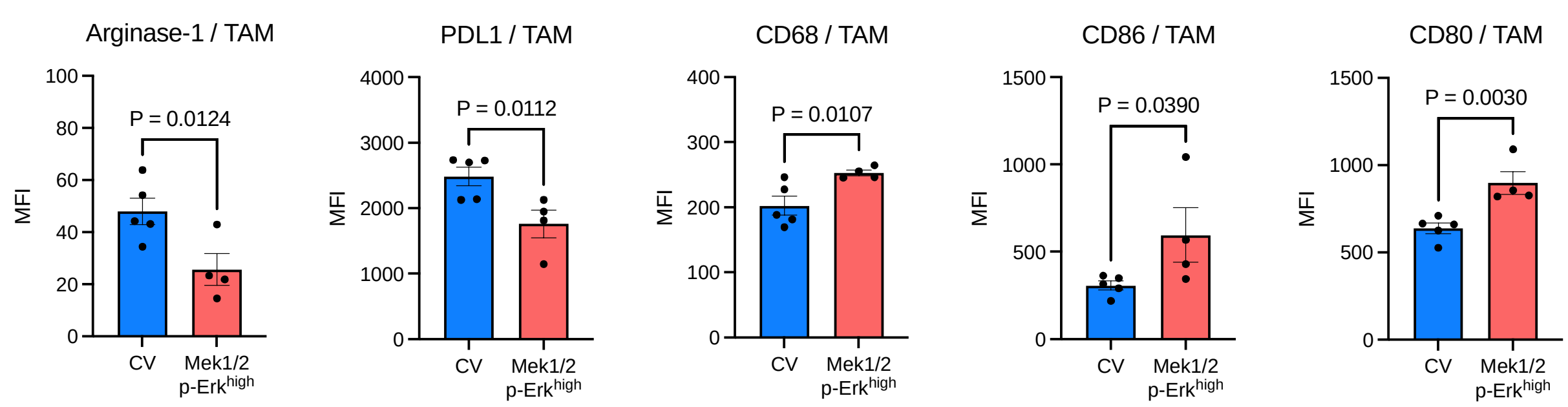

Figure 5. Phenotype of CD11b+ myeloid compartment from GL261 glioma-bearing brains.

Comment: In Figure 3, the authors should include a diagram of the experimental design, indicating the treatment initiation time and treatment schedule for the survival data shown.

Response: Thank you for your valuable comment. In response, we have updated Manuscript Figure 3 to include a detailed illustration of the experimental design for our survival study using the orthotopic GL261 model treated with FcE-aCTLA-4. Treatment was initiated on day 7 post-tumor injection, with a total of four treatments administered over two weeks. We have also enhanced this figure by adding new results on re-challenge experiment where wild-type GL261 tumor cells were implanted intracranially into the long-

term survivor mice that had previous MAPK-active GL261 treateded with FcE-aCTLA-4, and found that these had established immune memory and mostly rejected the tumors.

Comment: It would be required that the authors show some function assays related to interferon signaling on glioblastoma, both in their mouse models and human samples and how these are related to MAPK/ERK signaling pathway.

Response: We appreciate the reviewer's insightful comment. We respectfully like to point out that we have already provided functional data in the submitted manuscript, and kindly ask the reviewer to consider the following analyses that support the connection between MAPK/ERK signaling and interferon responses both *in vitro* and in human samples. First, as shown in Supplementary Figure 10 in the manuscript, we observed that the expression of interferon-response genes following treatment with interferon alpha was impaired when *ERK1* or *ERK2* were knocked out. In the same figure we show results of cytokine secretion assays following IFN-α treatment *in vitro* using non-targeting control (NTC) vs *ERK1* or *ERK2* knockout cells. The results indicate that MAPK/ERK pathway activity modulates type I interferon responses in glioma cells. Additionally, we utilized human GBM patient-derived slice cultures to examine the effect of MAPK inhibition via BRAFi/MEKi treatment. These *ex vivo* experiments further support the notion that suppression of MAPK signaling modulates interferon pathway activity in human GBM tissues. In mouse glioma cell lines, we also showed that *Erk1/2* knock-out leads to downregulation of *Ciita* and MHC class II molecules, which are interferon-response genes (Supplementary Figure 14 in the manuscript). Together, these data provide functional evidence linking the MAPK/ERK pathway to interferon signaling regulation across experimental models, cell lines, and species, including patient samples.

Comment: The same applies to the need to provide functional assays related to antigen presentation in order to provide functional validation for the conclusions put forward by the authors

Response: We thank the reviewer for highlighting the need to provide functional validation regarding antigen presentation. To address this, we employed a murine glioma model using CT-2A cells expressing ovalbumin (OVA), which enables precise evaluation of MHC class I–mediated antigen presentation. We performed flow cytometry to assess the expression of p-ERK, MHC class I-bound SIINFEKL peptide, as well as overall MHC class I and II surface levels. Interestingly, in this system, activation of the MAPK/ERK pathway via Mek1/2 overexpression did not impact MHC class I–mediated antigen presentation. However, consistent with our prior observations, MHC class II expression (measured by I-A/I-E molecule expression) was notably elevated upon Mek1/2 over-expression. These results suggest that MAPK/ERK signaling may predominantly regulate the expression of MHC class II in glioma cells. As we do not know whether this increase expression of MHC class II translates into improvement in antigen presentation, we

interpret this up-regulation as an interferon response, and have adjusted our claims throughout the manuscript to avoid stating that MAPK *per se*, promotes antigen presentation.

Comment: The data presented in Figure 7 also needs some functional validation in relation to the impact of these interactions in the biology they are reporting in this manuscript. It is felt that this is a correlative and secondary finding that does not contribute significantly to the primary and overarching message conveyed by this manuscript.

Response: We kindly like to point out that the results presented in Figure 7 from the *ex-vivo* slice culture model, and therefore provide causal evidence. Indeed, we performed an experimental perturbation of MAPK signaling with BRAF inhibition and investigated the effect of this intervention on the cross-talk between tumor cells and TAM. Nevertheless, we thank the reviewer for pointing out the need for additional functional validation regarding the cell–cell communication analysis presented in Figure 7. In response, we performed the following:

1) We phenotyped TAM from intracranial GL261 tumors with or without Mek1/2 over-expression, and observed a decrease in immunosuppressive markers such as Arginase-1 and PD-L1, with increase in phagocytic and antigen-presenting related markers in TAM derived from tumors with MAPK activation (Figure 5 of this document, and Supplementary Figure 15 of the manuscript).

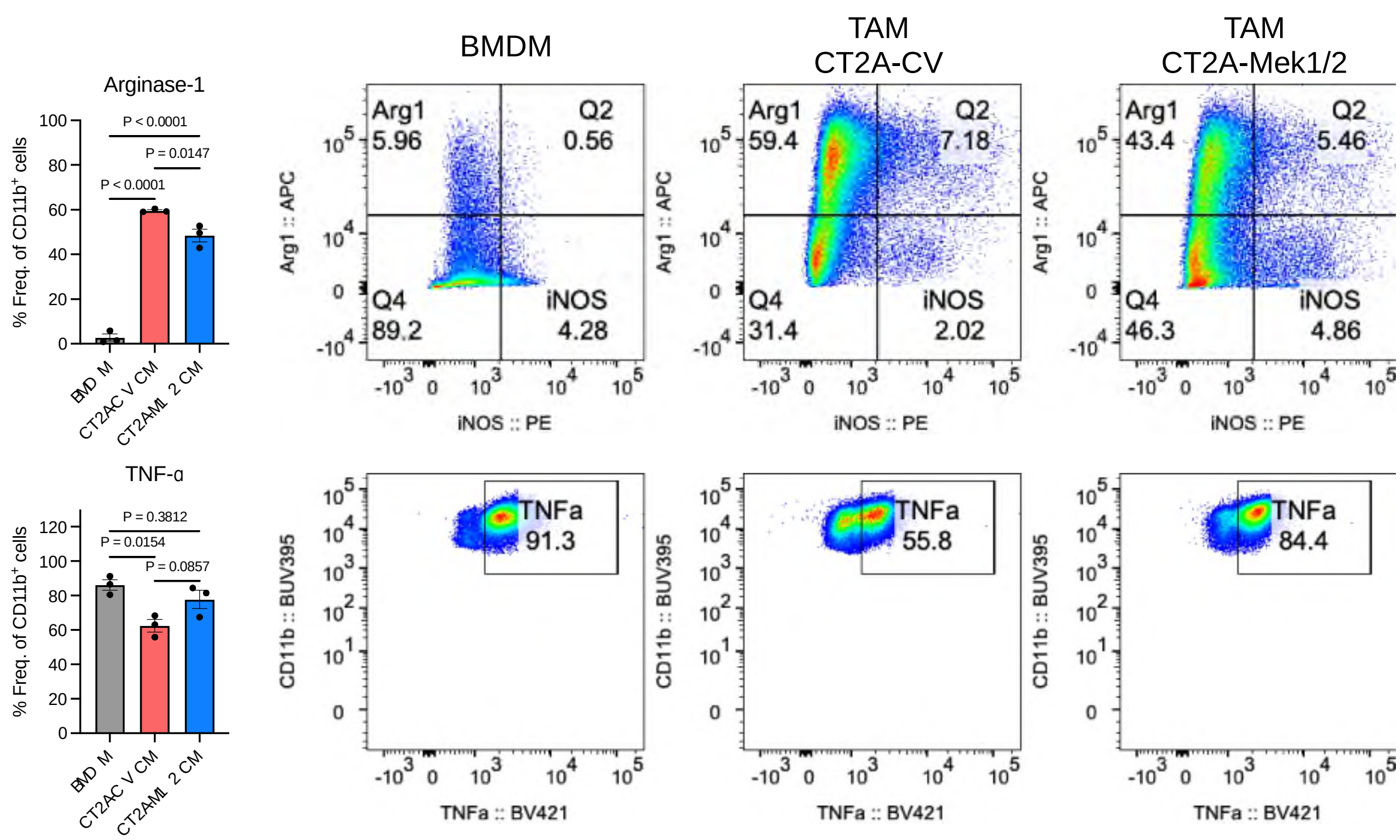

Figure 6. BMDM phenotypes treated with CT-2A-CV or -Mek1/2 conditioned media

2) *In vitro* experiments using bone marrow-derived monocytes (BMDMs) cultured with tumor-conditioned media from CT-2A glioma cells overexpressing either control vector (CV) or Mek1/2 (ERK-high condition). Flow cytometry analysis revealed that BMDMs treated with Mek1/2-overexpressing CT-2A-conditioned media exhibited reduced Arginase-1 expression and increased TNF-α production compared to those treated with CV-conditioned media (Figure 6 of this document, Figure 7C in the revised manuscript). These results indicate a shift toward a more immunogenic myeloid phenotype under ERK-high tumor influence, supporting our conclusion that MAPK/ERK activation in glioma cells impacts myeloid cell polarization and function. We have added these data to the revised manuscript to strengthen the functional basis of the interactions described in Manuscript Figure 7.
3) We used the MERFISH data to screen TAMs in the 1-hop NN to ERK-high/low tumor cells and found a significant increase of TNF-α production (gene set: BP.GO_TUMOR_NECROSIS_FACTOR_SECRETION) while ARG1 expression is significant reduced in the NN of ERK-high tumors (Figure 7 of this document, Figure 7D and E of the revised manuscript).

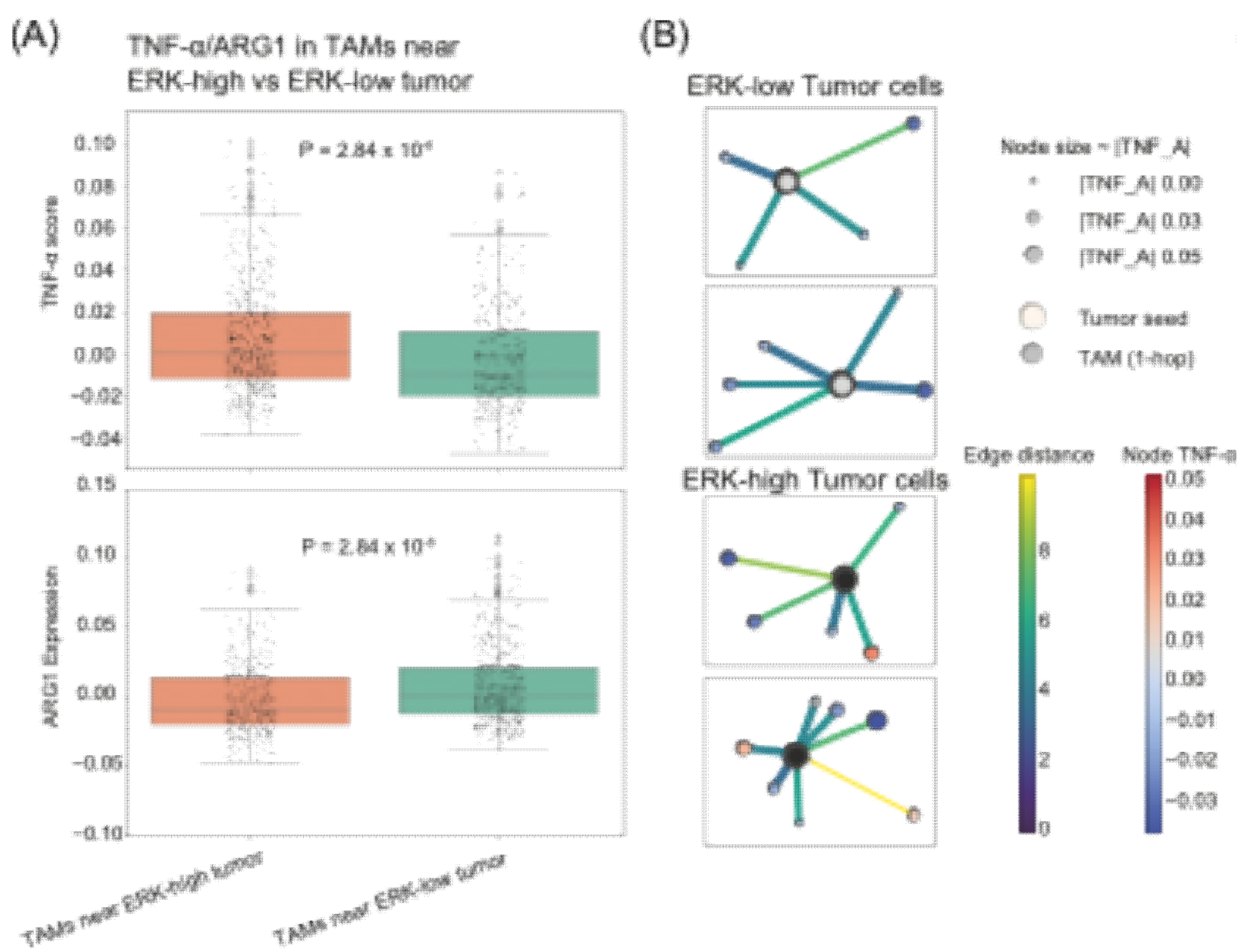

Figure 7. Spatial NN analysis from MERFISH data. (A) Relative expression of TNF-α (upper) or Arginase-1 (lower) in TAMs in the 1-hop NN from ERK-high / low tumors. (B) Examples of TAMs in the NN of tumor cells and the corresponding TNF-α expression.

Reviewer #3 (Remarks to the Author):

1. What are the noteworthy results?

Comment: How MAPK signaling in tumor cells shapes the microenvironment is an important question, since GBM can have upregulated MAPK signaling. Kim et al attempt to address if there is a link between MAPK activation and anti-tumor immunity in glioblastoma (GBM), in particular CD8+ T cell mediated immune editing. A previous paper from this group identified MAPK signaling as an enriched pathway in CD8 T-cell depleted mice. Here, Kim et al propose to provide the mechanisms underlying MAPK/ERK pathway signaling and susceptibility to immune checkpoint blockade. They use CRISPR-mediated genome editing to knock-out kinases and find that MAPK are the top enriched kinases in GL261-cells in CD8 KO mice vs wt mice. They provide preliminary evidence suggesting that MAPK signaling is functionally involved in immune editing and is linked to a CD8-mediated anti-tumor immune response. They attempt to prove that MAPK activation enhances glioma susceptibility to anti-PD-1 therapy and promote durable anti-tumor activity.

While each point is of importance, there is little novelty in this work. Their previous data already suggested that CD8+ T cells are employed to immune edit glioma cells (in a PDGF Pten-/- murine glioma model). In addition, there are other groups that have shown the same association. Moreover, the data generated have severe technical limitations which are further described below, and which further limit the enthusiasm for this work.

Response: We appreciate the reviewer's perspective but respectfully disagree. The previous manuscripts from our group that are cited do not show causality of MAPK signaling in tumor cells contributing to susceptibility of glioma cells to T cells, or immune modulation to be dependent on MAPK. Similarly, no previous studies, including those cited have shown that MAPK contributes to susceptibility of gliomas to immunotherapy. An association (previously reported in Arrieta et al., Nature Cancer, 2021) does not necessarily imply causality. This is one of the main findings our manuscript, as we show that MAPK signaling in tumor cells can modulate the tumor cell immunogenicity, tumor-microglia crosstalk, and contribute to T cell infiltration, recognition, and ultimately, susceptibility to immunotherapy. We also describe the mechanisms involved in this process, including IFN responses.

Comment: They fail to cite important literature of relevance to their field of research. For example, it is well known that in pediatric glioma a PXA-like tumor subtype is enriched for $CD8^+$ T cells and BRAF mutations, already suggesting that increased MAPK signaling (BRAF mutations) in high-grade gliomas associated with increased immunogenicity (PMCID: PMC5956280).

Response: We thank the reviewer for bringing this up, as we do consider this reference relevant, and in response, we have now cited it, as supportive evidence of our findings. Yet, please consider that this citation, and all previous work on this topic shows associations between MAPK and immunogenicity, but not evidence of MAPK being causally implicated, or driving tumor cell immunogenicity and susceptibility to immunotherapy.

Comment: Kim et al use CD8 KO mice in this manuscript, which is not a conventional approach in immunology work (see also comment below). CD8 KO mice have CD4 T cells which could compensate for the lack of CD8 T cells. As shown in an older study using CD8/CD4 double knockout mice, alternative T cells could compensate for the lack of CD8/CD4 T cells (PMID: 8500525). The various T cell populations documented in glioma need to be quantified in the CD8 KO mice bearing murine glioma, to gain mechanistic insights and provide support for their claims that high MAPK signaling is immune edited.

Response: We thank the reviewer for this thoughtful comment. While we acknowledge the possibility that compensatory mechanisms involving $CD4^+$ or unconventional T cells may arise in *Cd8* KO mice as reported in PMID: 8500525, we selected this model because it offers a clean and specific genetic depletion of cytotoxic $CD8^+$ T cells, which allowed us to evaluate their indispensable role in mediating the MAPK/ERK-related immune response in glioma. In our study, the CRISPR screen results, along with the lack of therapeutic efficacy observed in *Cd8* KO mice, support the conclusion that MAPK/ERK-driven immunogenicity in glioma is largely dependent on $CD8^+$ T cells.

To further clarify the immune context in this model, we have now cited our previous work (Dmello et al., Nature Comm. 2023), where we comprehensively profiled immune subsets in *Cd8* KO glioma-bearing mice. That analysis demonstrated that $CD8^+$ T cells are the primary population depleted, as shown in Figure 8 of this document extracted from that previous paper, shown below for the reviewer's convenience. We observe no compensatory expansion of other effector subsets. We have included this information and citation in the revised Discussion section to better address the reviewer's concern and to strengthen the mechanistic interpretation of our findings.

2. Will the work be of significance to the field and related fields? How does it compare to the established literature? If the work is not original, please provide relevant references.

Comment: The work provides very incremental progress from a previous study published in 2020 by this group (PMCID: PMC7442699).

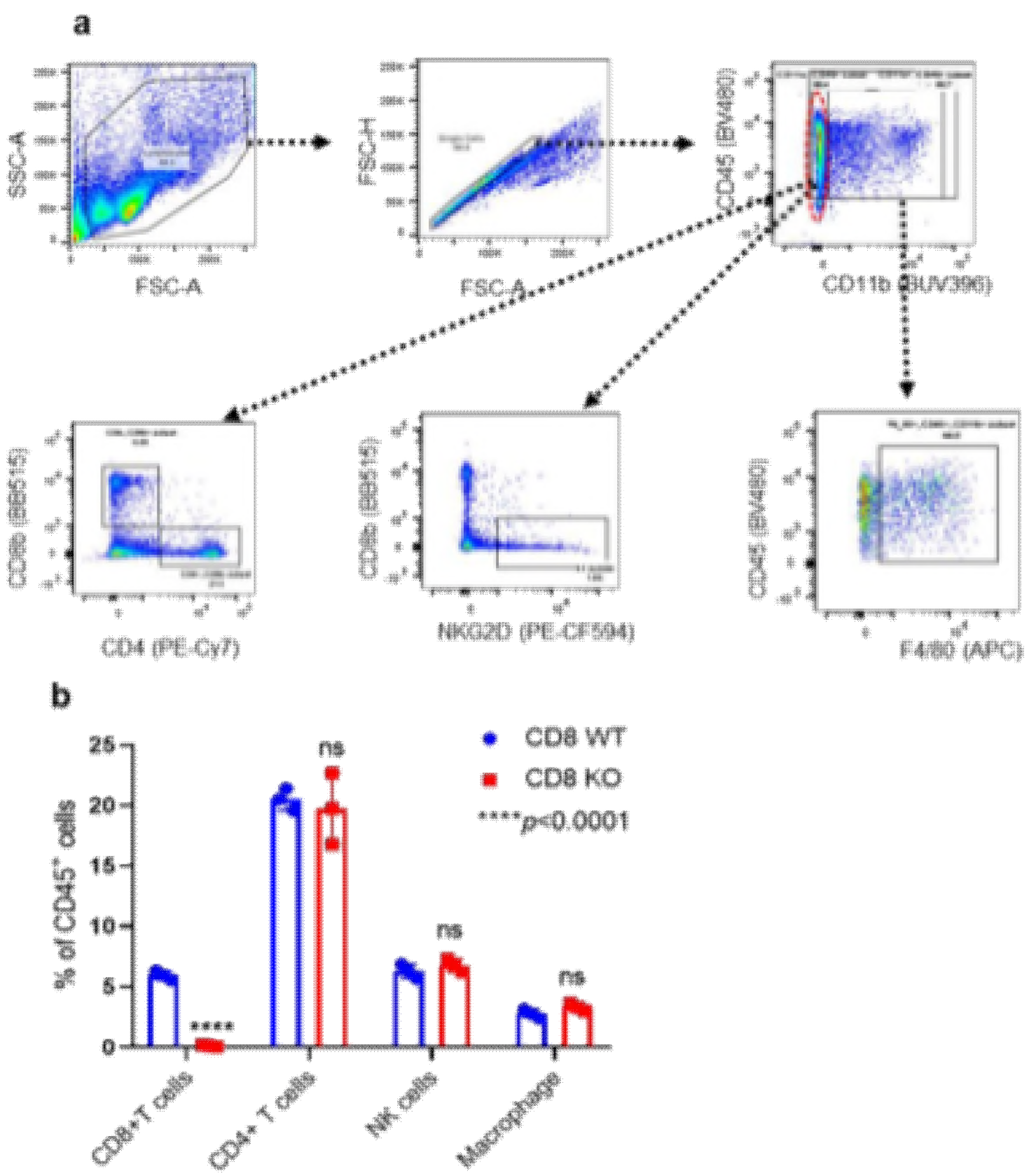

Figure 8. Supplementary Figure 1 from Dmello et al., Nature Comm. 2023.

Response: The reference mentioned by the reviewer showed that glioma-genesis in the absence of $CD8^+$ T cells promotes the development of more immunogenic tumors, which have MAPK activation, but does not show that MAPK per se is responsible for glioma immunogenicity, or modulating response to different forms of immunotherapy. That work also did not include human GBM data. The publication cited also does not provide causal evidence or mechanistic insight on MAPK driving response of gliomas to immunotherapy, an association we previously reported. Yet to the best of our knowledge, until this manuscript, the implication of MAPK on the modulation of immune response in gliomas has not been experimentally investigated. In particular, the fact that MAPK drives susceptibility to T cells and various forms of immunotherapy through enhancement of IFN responses, modulation of microglia phenotype, and an increase in T cell infiltration is novel.

3. Does the work support the conclusions and claims, or is additional evidence needed?

Comment: The work only partially supports the conclusions and claims. Yes, additional evidence is needed as mentioned below. The authors' claim that they provide a mechanistic link of MAPK pathway activation (p-ERK), and immune evasion is not

supported by their data. How do they propose the data could benefit patients? The clinical translation should be discussed in more detail.

Response: We appreciate the reviewer's comment, but we would like to clarify that our data support the opposite conclusion to that stated by the reviewer above: we do not claim that MAPK activation is linked to immune evasion, but the contrary. The findings of the manuscript establish causal evidence that MAPK drives glioma immunogenicity and response to immunotherapy and explain several clinical trial and human cohort analyses where we observed MAPK to identify patients that exhibit durable responses and favorable survival following immune checkpoint blockade (Zhao et al., Nature Med., 2019; Arrieta et al., Nature Cancer, 2021; Arrieta et al., CCR, 2024). For the first time, we report that MAPK susceptibility to immunotherapy is applicable beyond aPD-1 immune checkpoint blockade immunotherapy and is also enhances responses to a novel form of immunotherapy with FcE-aCTLA-4, currently being tested on a clinical trial (NCT05864534), and ro CD8 T-cell immune response in general. Based on these results, we are now investigating whether MAPK can identify patients who benefit from this treatment in this clinical trial. More importantly, as the mechanism we describe for MAPK promoting immunotherapy response involves IFN and T cell responses, our manuscript opens up the possibility of MAPK signaling serving as a biomarker for other emerging forms of GBM immunotherapy such as B cell vaccines, CAR T cells BiTEs, etc. While therapeutic modulation of MAPK is outside the scope of the current manuscript, our study sets the foundation to investigate this as a strategy to enhance glioma susceptibility to immunotherapy for patients who currently do not benefit from these treatments.

4. Are there any flaws in the data analysis, interpretation and conclusions? Do these prohibit publication or require revision? Is the methodology sound? Does the work meet the expected standards in your field?

Comment: The methodology and approach are a major weakness of this manuscript. Validation experiments are essentially missing from the entire manuscript. Most experiments were performed in a single cell line (AM38) or in just one murine glioma model (GL261).

Of particular concern are their choice of murine and human models. GL-261 is a poor model for GBM and fails to recapitulate the genetics and phenotypes of the human disease. GL-261 is hypermutated and is partially immunogenic. This casts doubts on the results from the CRISPR screen, which was just performed in this GL261 model. They need to show the CRISPR screen and MAPK over expression experiments in at least two murine glioma models side-by-side, and the models need to be more relevant to the disease.

Response: We thank the reviewer for raising this important concern. While we acknowledge the limitations of the GL261 model, we respectfully disagree with the

assertion that our findings are insufficiently supported due to our choice for the model. We provide data on GL261, QPP7, AM38 human glioma cell line, as well as a primary culture of a GBM sample with BRAF mutation in human brain slices, as well as scRNA-seq, multiplex IF on 27 GBM patient samples, and spatial transcriptomic data on an additional 27 GBM patients, and scRNA-seq of over 100 GBM patients. Yet, to address this critique, we have now included data on CT-2A. As shown in Figure 9, we observed similar results to GL261 tumors in our CT-2A flow cytometry analysis. Moreover, from multiplex histology data, we quantified CD8$^+$ T cells infiltration is higher in Mek1/2 overexpression high p-Erk tumor (Fig. 9 of this document). All the relevant additional new data on CT-2A have been included in Supplementary Figure 3.

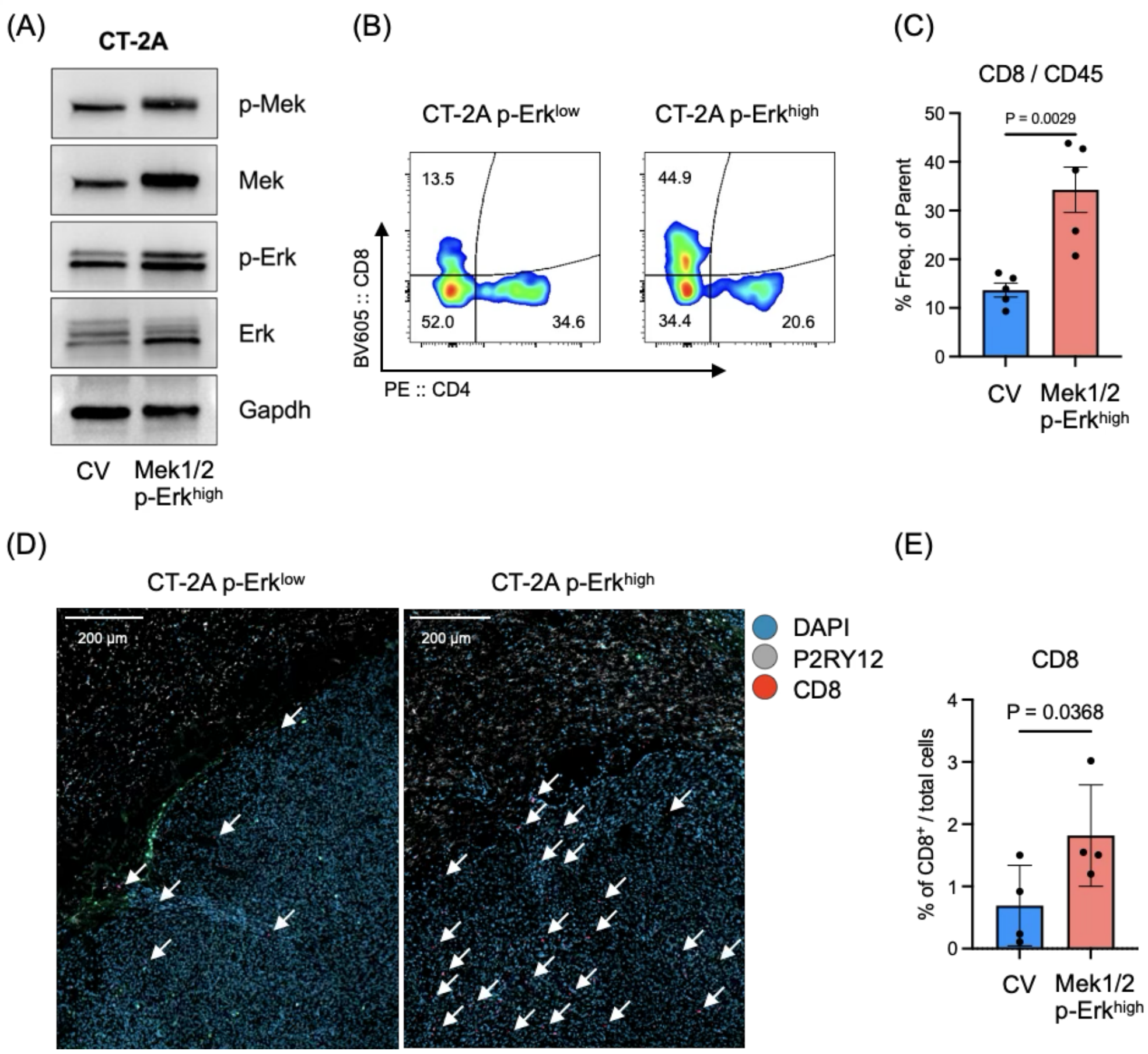

Figure 9. CT-2A tumor analysis.

We have also conducted a pilot CRISPR screen in CT-2A model (Figure 10 of this document). Interestingly, we observed that CT-2A cells transduced with the sgRNA library and Cas9 exhibited reduced tumor engraftment capacity *in vivo*—only 3 of 15 mice developed tumors—suggesting that expression of the CRISPR machinery may enhance immunogenicity in this particular model. We have included this data for the reviewer's convenience; however, we have decided not to pursue the CT-2A glioma *in vivo* CRISPR screening as results would be artefactual. Please consider that performing CRISPR

screens in multiple models is not necessary, as we have validated the MAPK-related findings across different models, species and systems, while avoiding any functional claims on other hits from the CRISPR screen that were not carefully validated for the manuscript.

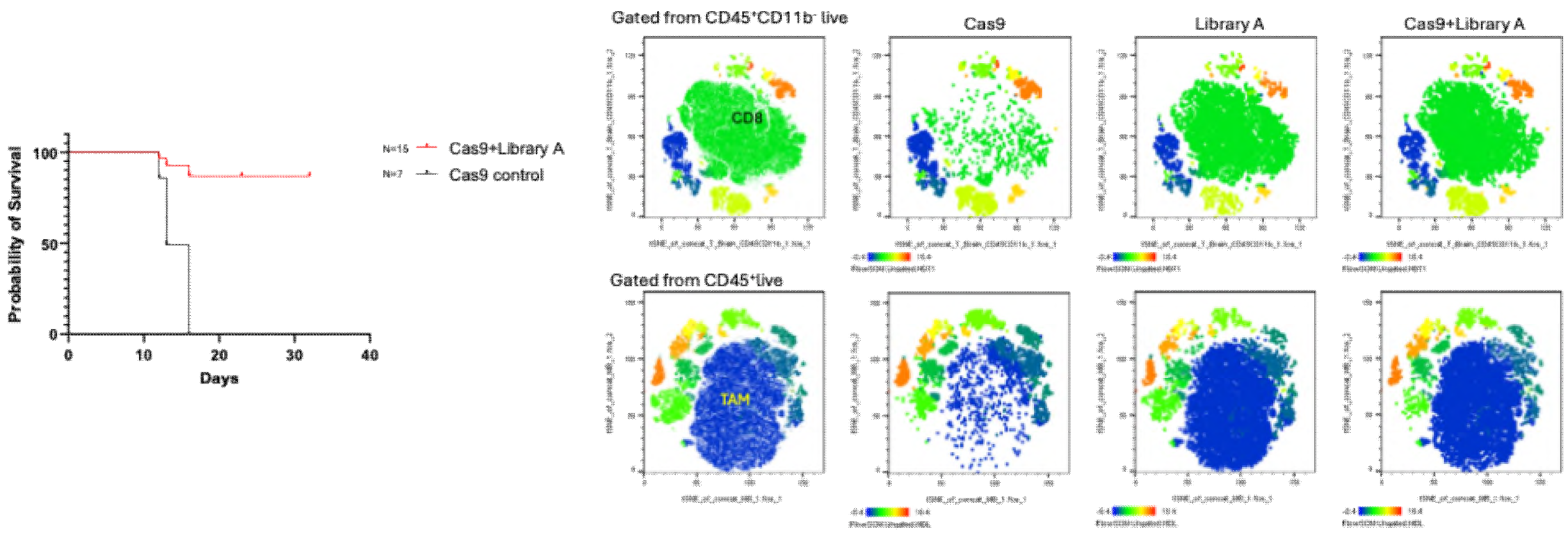

Figure 10. CRISPR screening in CT-2A in vivo model.

Comment: There are also dramatic inconsistencies with the existing literature, which are not reconciled in the discussion. As an example, GL261 is a hypermutated cell line sensitive to immune checkpoint blockade (PMCID: PMC5724383). However, Kim et al show a minor response to aPD-1 immunotherapy.

Response: We thank the reviewer for pointing this out. Indeed, there are differences in GL261 susceptibility to immunotherapy, as noted, including several reports of clones that are resistant to immunotherapy. Differences in GL261 susceptibility to immunotherapy have been widely reported, and depend on clones (Kim et al., Neuro-Oncol., 2024; Arrieta et al., Nature Comm., 2024; Dmello et al., Nature Comm., 2023). As clarification, our GL261 cells were obtained from NCI. Cells undergo regular STR profiling, and are routinely tested for mycoplasma contamination. These cells are truly resistant to immunotherapy, similar to what has been previously reported by several groups. In response, we have added a sentence to the revised manuscript acknowledging that different clones GL261 cells have been reported to have variable response to immunotherapy (Reardon et al., Cancer Res., 2016).

Comment: In addition, AM38 is a serum-grown cell line for a BRAF V600E mutant GBM. Why did they not use additional GBM cell lines with MAPK pathway activation? Experiments in a single cell line are uninformative. Additional biological replicates are needed to provide credibility to the statements made by this group.

Response: We appreciate the reviewer's important comment. We selected AM38 as a well-established human GBM cell line harboring the $BRAF^{V600E}$ mutation, which serves as a representative model for MAPK/ERK pathway activation. This line allowed us to mechanistically assess the downstream consequences of ERK modulation in a

genetically defined human context. Yet, we have performed extensive validation of the findings, including in human GBM specimens. Specifically, we incorporated *ex vivo* slice cultures from a $BRAF^{V600E}$ human GBM shortly after being excised from a patient, where we observed consistent changes in interferon signaling and tumor immunogenicity following BRAFi/MEKi treatment. Furthermore, we conducted additional experiments using murine glioma models (GL261 and CT-2A) to evaluate ERK modulation through genetic overexpression. These models consistently recapitulated the immune phenotypes associated with MAPK pathway regulation. Finally, to expand the translational relevance of our findings, we performed immunofluorescence and transcriptomic analyses across a cohort of human GBM samples, using scRNA-seq, spatial transcriptomics. These data robustly support the central role of MAPK/ERK signaling in shaping interferon responses and immune susceptibility in GBM. We have further clarified this rationale in the revised manuscript.

5. Is there enough detail provided in the methods for the work to be reproduced?

Comment: Each experiment needs to be described in much greater detail to be informative for the readership. As they stand right now, the methods are not described in sufficient detail to be recapitulated elsewhere. For example, the lentivirus library is not described at all (is this a pan-kinase library?). Information about drug concentration and treatment schedules is missing.

Response: We appreciate the reviewer's feedback pointing out the need for more detailed descriptions in our methods section to enable reproducibility. We have thoroughly revised this section to include comprehensive details. Specifically, we used the Brie mouse CRISPR knockout pooled library for our kinome-wide CRISPR/Cas9 screening. This library targets a wide range of kinase genes, allowing for a comprehensive assessment of potential regulatory elements in tumor response mechanisms. Additionally, we have now included precise concentrations and treatment schedules for all drugs and interventions used throughout our experiments in the Method section.

6. Additional points that need to be addressed.

Comment: What are the effects of MAPK activation on IFN signaling, T cells, and responsiveness to immunotherapy in recurrent tumors? Since this type of tumor is usually resistant to BRAFi+MEKi treatment, it is not clear whether tumors in this study are responsive to targeted therapy or resistant, since reactivation of the MAPK pathway in recurrent tumors is one of the resistance mechanisms.

Response: Thank you for this question. Our findings revealed the efficacy of BRAFi+MEKi to some extent (Supplementary Figure 11 panel B of the manuscript). Both types of interferon response as well as $CD8^+$ T cell activation, were downregulated after BRAFi+MEKi (Figure 5 of the manuscript). Of note, the effects of MAPK inhibition on

tumor growth are tangential to the immune-modulatory effect and interferon response reliance on MAPK that we study here.

Comment: Since MAPK activation improved the efficacy of anti-CTLA-4 immune checkpoint blockade in addition to anti-PD-1 treatment, the authors should study the effect of combining aPD-1 and aCTLA-4 blockade therapy using Mek1/2-overexpressed mice?

Response: We thank the reviewer for this interesting idea. In these mouse glioma models, we cannot evaluate the efficacy of the combinatorial immunotherapy vs monotherapy, as monotherapies with Mek1/2, led to cure in most mice, leaving no room for improvement. We are evaluating p-ERK in the setting of this immunotherapy combination in an ongoing clinical trial (NCT 05864534).

Comment: The authors found downregulation of IFN signatures and HLA molecules in BRAFi/MEKi-treated samples using a patient-derived slice culture model compared to TMZ treatment. However, it is unclear how BRAFi+MEKi treatment affects MAPK signaling and IFN and HLA signatures during 7 days of treatment. A time-dependent analysis of these processes is required to determine the expression level of IFN signatures and HLA molecules at different time points during treatment and potentially link them to each other.

Response: We appreciate this valuable suggestion. However, unfortunately, these time course experiments cannot be repeated, as the original BRAF-mutant human tumor used is no longer available for additional experiments, and on the other hand, BRAF mutant GBM is a relatively rare, so it is not practical to wait to get another case for completing this manuscript revision.

Minor points

Comment: The figure legend for Supplementary Figure 5B mentioned Type I interferon signaling, but the figure and text indicated Type II Interferon signaling. Please revise.

Response: We thank the reviewer for the comment, and we have corrected this figure, which is now Supplementary Figure 8 of the manuscript.

Comment: Representative images for cell proliferation assay are missing in Supplementary Figure 6B.

Response: We thank the reviewer for the comment, and we have added the representative pictures as requested, on the revised manuscript, this is now Supplementary Figure 9.

Comment: Representative images for IF analysis are missing in Supplementary Figures 4 and 9. The figure legend for Supplementary Figure 8 is not well described.

Response: The representative images for Supplementary Figure 4 was on the main figure of the original manuscript, yet we have now removed these results on immunofluorescence staining for IRF9. The representative images for Supplementary Figure 9, are now included on the main Figure 6A. As mentioned above, we have now corrected the legend for Supplementary Figure 8.

Comment: Why did the authors focus on the expression of MHC II-related transcripts after ERK2 KO in AM38 cells (Supplementary Figure 10)? Does ERK2 KO also downregulate MHC I-related gene transcripts? Since "antigen processing MHC I" is shown in the AM38-NTC from GSEA analysis (Figure 5B).

Response: We thank the reviewer for this comment. From bulk RNA-seq from AM38 ERK1/2 KO cells, we identified multiple gene signatures related to antigen presenting process. In our study, the choice to highlight MHC II-related transcripts was driven by preliminary findings suggesting that ERK2 particularly influences MHC II expression, as a readout for IFN responses.

Comment: It is interesting to see that BRAFi/MEKi treatment significantly decreased the crosstalk between tumor cells and microglia (I assume by comparing with TMZ treatment), and modulated microglia/TAM phenotypes by increasing anti-inflammatory monocytes.

Response: Thank you for comments. We were able to provide additional validation of this finding by our new MERSFISH analysis, as well as *ex-vivo* and in vitro validation in the murine glioma models GL261 and CT-2A (Supplementary Figures 15 and Figure 7 of the manuscript).

Comment: Given that IFN signaling is associated with cytotoxicity, is there a correlation between MAPK activation and inflammation? Do Mek1/2-overexpressed samples secrete more pro-inflammatory cytokines/chemokines in the tumor microenvironment? In addition, since TMZ treatment is used for comparison, how does the cell-cell communication network look in the untreated (control-treated) tumor sample?

Response: Thank you for the insightful comments. We include data in human and in mouse glioma cell lines, where we observe that MAPK signaling in glioma cells promotes expression of cytokines and chemokines, including CCL3, CCL4 and TNFα, in response to IFN in mouse gliomas, human cell lines, and in human GBM (Supplementary Figure 10 and Figure 7 of the manuscript). We have now added the correlation between MAPK activation and $CD8^+$ T cell activation, as well as the cell-cell communication for the control condition in slice culture.

Comment: In Figure 7A, BRAFi+MEKi treatment seems to kill more NPC-like tumor cells compared to TMZ treatment, it would be good to explain this observation.

Response: While we don’t have the data to imply that a subset of tumor cell phenotypes is more susceptible to BRAFi+MEKi, we do appreciate the observation made by the reviewer. By the way, for the revised Figure 7, we have replaced the TMZ condition for a control condition, as requested by the reviewer, and the same observation applies in this case too.

Reviewer #4 (Remarks to the Author):

Kim et al reported that MAPK/ERK activation in tumor cells increased interferon responses and T cell activity, sensitizing mouse GBMM tumors to immune checkpoint blockade. Using a kinome-wide CRIPSR screen, the author identified that MAPK/ERK is a regulator of tumor resistance to anti-PD1 treatment. They showed that overexpression of MARK/ERK sensitizes GL261 tumors to checkpoint inhibitors. Elevated p-ERK in glioma cells was associated with increased interferon responses, antigen presentation and T cell infiltration in GBM. Overall, this is an interesting manuscript. However, some findings are descriptive. The authors may address the following concerns to improve their study:

Comment: There is a major concern regarding the rationale of the CRISPR screen. GL261 glioma cells lentivirally transduced with a sgRNA library were implanted intracranially into wild-type and Cd8 KO mice, and the enriched kinase CRISPR was identified in the Cd8 KO mice. This screen will identify not only the kinases that regulate tumor cell response to T cells, but also the kinases that directly contribute to tumor cell proliferation. Therefore, MAPK/ERK can be identified as it induces tumor cell proliferation. In addition, GL261 is a suboptimal model for studying GBM resistance to checkpoint inhibitors and for the screen, as it does not recapitulate the clinical responses.

Response: We appreciate the reviewer's comment and agree that this screen, when comparing KO clones that enriched over time (relative to the initial representation of these clones at time of implantation), can identify kinases regulating tumor cell proliferation. However, the time-driven selection pressure was applied equally to both the control and treatment groups. Our gene hits were identified through a multilevel variant analysis, which first normalized each group to the same baseline control before comparing the control and treatment groups (see MAGEcK MLE workflow). This approach mitigates potential false positives arising from time-selection pressure. To further support this, we conducted a random permutation test, selecting a gene set of the same size as our significant hits from the BRIE library dataset for pathway enrichment analysis. The resulting analysis did not identify the MAPK pathway as significantly enriched (Figure 11 of this document, Supplementary Figure 2 of the manuscript).

Furthermore, the design of the CRISPR screen analysis by comparing *Cd8* KO vs wild type, focuses on gene KO that are selected out by $CD8^+$ T cells, regardless of their tumor growth contribution (which happens in both conditions). The choice of GL261 for this experiment is based on its susceptibility to $CD8^+$ T cells, as we have shown before (Dmello et al., Nature Comm., 2023). Regardless of the choice of glioma model for this screen, the results we focused on are validated across multiple *in vitro, in vivo, ex vivo* experiments and analyses in gliomas across mouse and human species. As mentioned above, we performed a pilot CRISPR screen in the CT-2A mouse glioma model, and

observed that in such model, there are artefacts, and immunogenicity intrinsic to CRISPR, that would not allow us to perform the same expertiment we present with GL261, as tumors expressing Cas9 did not readily engraft (Figure 10 of this document).

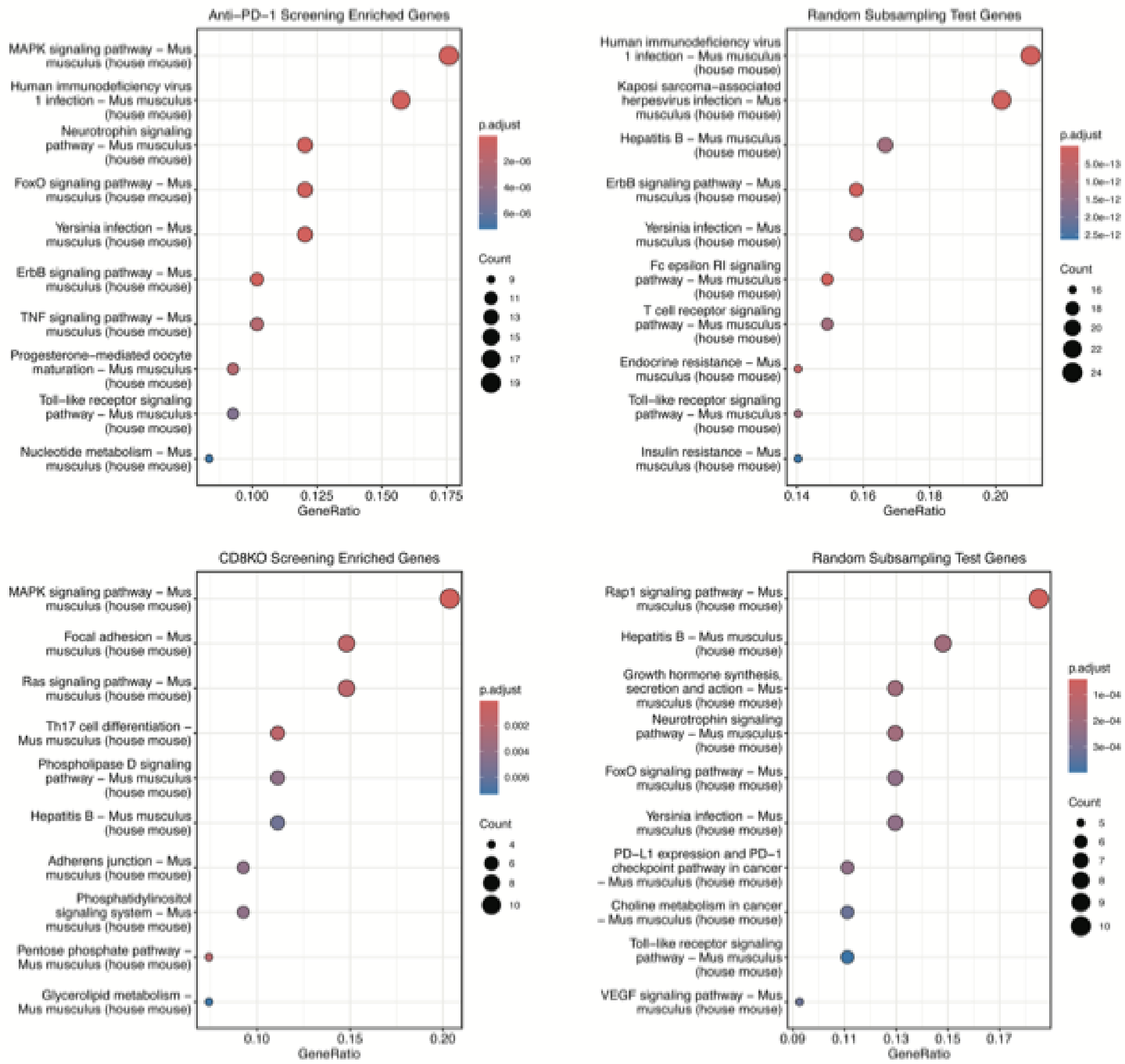

Figure 11. Random permutation test of CRISPR/Cas9 screening dataset.

Comment: The potential translational impact is relatively moderate. Will the authors propose to use activation of MARK/ERK as a strategy to treat GBM? This apparently will increase tumor growth.

Response: We thank the reviewer for the insightful comment on the translational impact of our study. We understand the concerns regarding the potential for MAPK/ERK pathway activation to promote tumor growth, given its well-established role as an oncogene. Indeed, the activation of this pathway is typically associated with tumor progression in various cancers, including GBM. Our previous clinical findings have shown that the response to immune checkpoint inhibitors, such as nivolumab, can be modulated by the activity of the MAPK/ERK pathway. This suggests that while the activation of MAPK/ERK pathway may generally promote tumor growth, it also enhances the immunogenicity of

the tumor, making it more susceptible to immune checkpoint blockade. In this study, we propose that under certain conditions, specifically within the context of immunotherapy, activating this pathway could indeed serve as a viable strategy to enhance treatment efficacy. This is supported by our data showing that ERK activation correlates with increased interferon responses and T cell infiltration, which are critical for the effective response to immunotherapy. While our findings suggest a potential for therapeutic strategies that might combine MAPK/ERK pathway activation with immune checkpoint inhibitors to counterbalance the pathway’s tumor-promoting effects while enhancing the immune response, this therapeutic strategy is out of the scope of this current manuscript.

Comment: No mechanism study to investigate how MARK/ERK activation induces interferon responses and stimulates T cell activation.

Response: Thank you for your comment. While our study primarily establishes the causal link of MAPK/ERK activation leading to enhanced immune responses, including interferon signaling and T cell activity, we recognize the importance of elucidating the underlying mechanisms driving these observations. To address this, we performed cytokine profiling and demonstrated that MAPK/ERK pathway activation regulates the secretion of cytokines relevant to immune stimulation, following IFN exposure. In particular, tumors with high ERK activity exhibited elevated expression of IFN-responsive genes, cytokines, chemokines, resulted in increased crosstalk with TAMs, and T cell recruitment. These features suggest that MAPK/ERK activation may foster a more immunologically permissive tumor microenvironment, facilitating T cell infiltration and activation.

Regarding the mechanism by which p-ERK modulates IFN responses in gliomas, we mentioned in the discussion that this is consistent with previous literature implicating MAPK/ERK signaling in the IFN response in other settings (Kiyatkin et al., Sci Signal, 2020; Mazewski et al., Front Immunol., 2020). This regulation occurs through the activation of signaling pathways, including STAT1 drives the expression of genes involved in immune responses (Banik et al., J Breast Cancer, 2021).

Comment: There is no solid data supporting the conclusion in the mechanism study. Most of results are based on association studies. Particularly, there is no experimental data supporting the microglial phenotypes: only a prediction based on cell-to-cell communication analysis of slice cultivated human GBM samples treated with temozolomide.

Response: We thank the reviewer for raising this. First we want to clarify that for the experiment of slice culture, we treated the BRAF mutant GBM tumors, which have elevated MAPK activity with either control condition, TMZ, or BRAF/MEK inhibition, and we obseve that in the condition where MAPK signal is inhibited, there is decrease in tumor-microglia crosstalk, thus this experimental data supportive of a causal link between MAPK and tumor-microglia interactions, not an association. Yet to further investigate this,

we now include functional validation of the microglial and myeloid phenotypes to complement the findings observed in our *ex vivo* slice culture experiment mentioned above. We performed additional *in vitro* experiments using bone marrow–derived monocytes (BMDMs) as a surrogate model to investigate tumor-conditioned modulation of myeloid cell states. Specifically, we isolated BMDMs from mouse femurs and cultured them with tumor-conditioned media derived from glioma cells. We then analyzed the expression of Arginase-1, TNF-α, and PD-L1 via flow cytometry. Compared to control conditions, monocytes exposed to tumor-conditioned media displayed increased Arginase-1 and reduced TNF-α expression, consistent with a shift toward a tumor-associated myeloid phenotype. Notably, this phenotype was reversed when monocytes were cultured with conditioned media from high p-Erk tumors, suggesting that MAPK/ERK activation in tumor cells modulates the differentiation and functional polarization of surrounding myeloid cells by a mechanism that to some degree, involves soluble factors (Figure 6 of this document, Figure 7 of the manuscript). These results provide mechanistic support beyond association and substantiate our conclusions regarding microglial/myeloid modulation. We also observed that TAM from GL261 gliomas with elevated MAPK, exhibited a decrease in immunosuppressive markers such as Arginase-1, PD-L1, but increased the expression of markers CD68, CD86 and CD80 (Figure 5 of this document, supplementary Figure 15). Additional validation of microglial phenotypes influenced by interactions with high p-ERK tumor cells is supported by our MERFISH data. In human patient samples, tumor cells with high ERK activity were found in closer proximity to inflammatory microglia, further supporting the notion that MAPK/ERK signaling modulates microglial activation states (Figure 4 of this document).

Minor

Comment: Fig. 1. A snapshot of whole kinome data would be helpful to see the global changes. Any positive or negative control?

Response: We appreciate the reviewer’s suggestion. The global kinome screening results for the CD8 KO condition have been comprehensively reported in our previous publication (Figure 1 in Dmello et al., Nature Comm. 2024), which provided a broad overview of the experiment. In that study, we reported kinases that are involved in immune evasion by glioma cells. Positive and negative controls (e.g., non-targeting sgRNAs) were included in the original library and screen, as detailed in the Methods section and as previously reported in Dmello et al. We have mentioned this in the revised manuscript for further clarity (Supplementary Figure 1C and D of the manuscript and Figure 12 in this document).

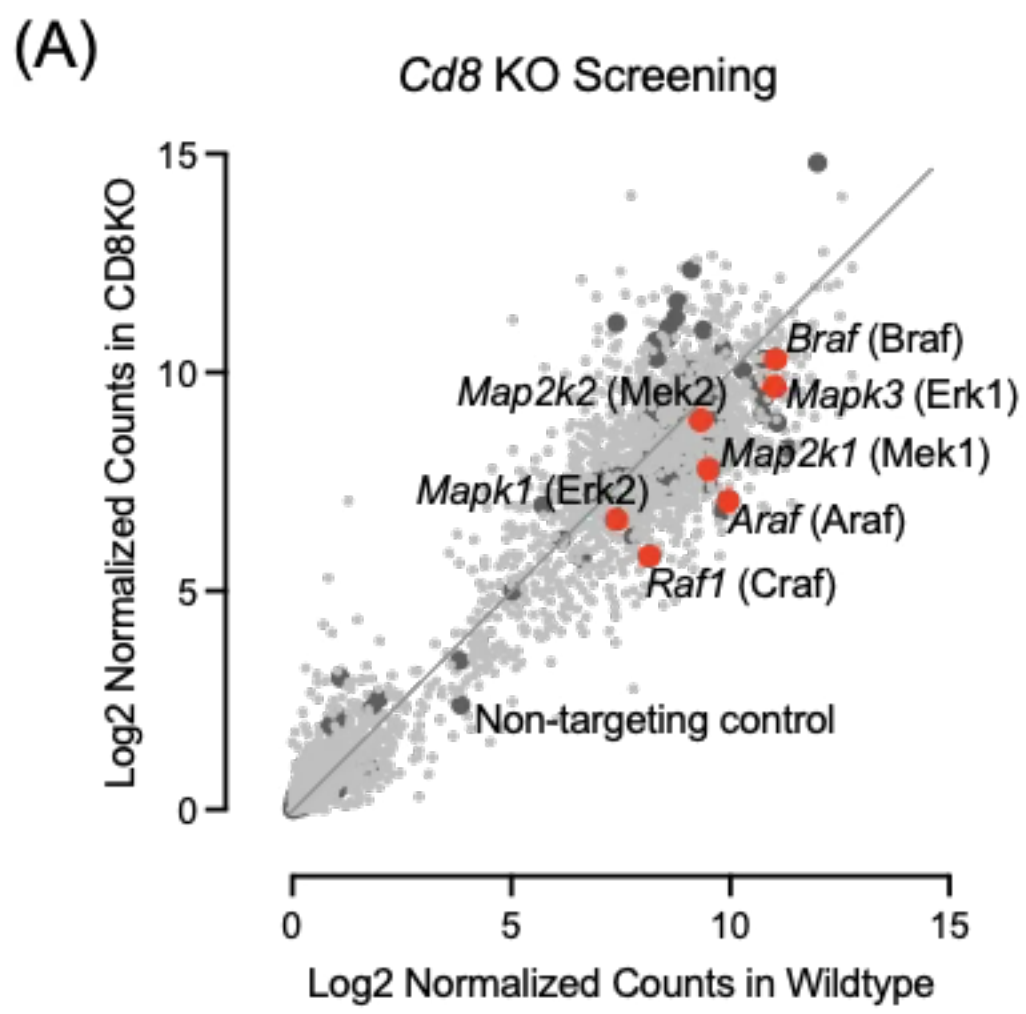

Figure 12. (A) Overall scatter plot showing kinases with enriched or depleted sgRNAs in the wild-type compared to Cd8 KO mice. Red dots represent the MAPK/ERK pathway-targeting sgRNAs; gray dots indicate non-targeting control sgRNAs. (B) Overall scatter plot showing kinases with enriched or depleted sgRNAs in anti-PD-1-treated mice compared to controls. Red dots represent the MAPK/ERK pathway-targeting sgRNAs; gray dots indicate non-targeting control sgRNAs.

Comment: Fig. 2. The authors may also show the effects of expression of constitutively active MEK/ERK mutants in addition to overexpression of the kinase.

Response: We appreciate the reviewer to point this out. As requested, we have explored the effects of a constitutively active MEK mutant in our murine glioma cell line (Figure 13 of this document). The results were consistent with those observed with overexpression of the wildtype kinase, reinforcing the role of the MEK/ERK pathway in modulating tumor immunogenicity. However, we did not focus on this mutant as a model out of concern for its potential artefactual immunogenicity by the mutant protein.

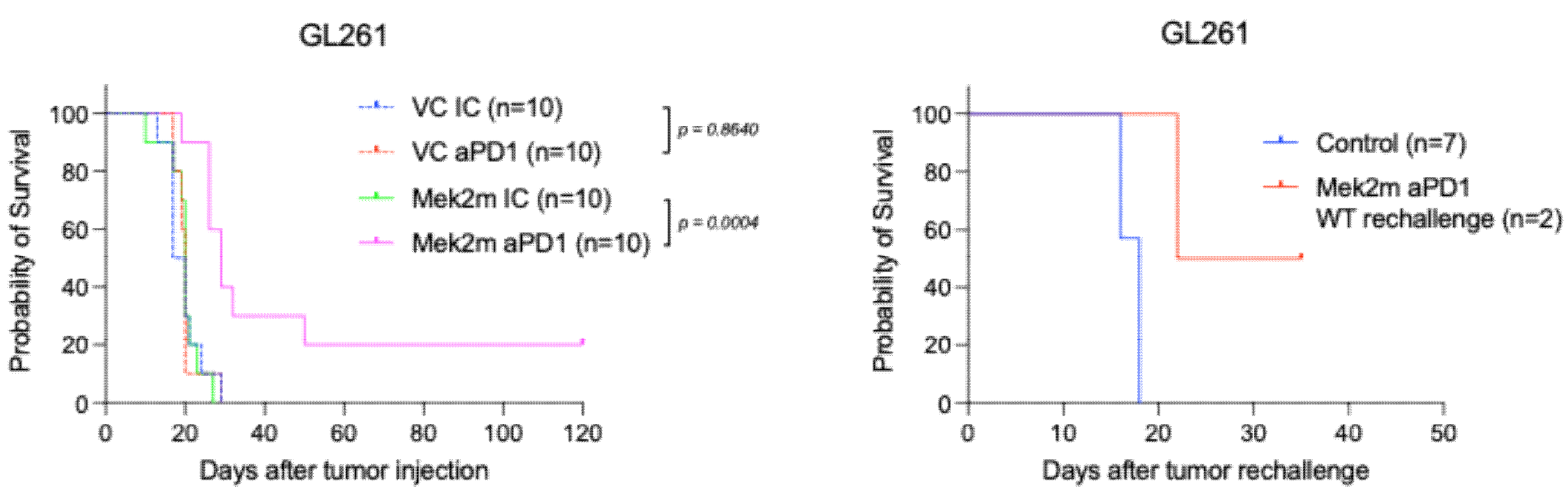

Figure 13. Kaplan Meier survival study using constitutive active Mek2 (Mek2m) isoform.

Comment: Fig. 2D-G. No tumor growth data shown. Will MAPK/ERK activation increase tumor growth?

Response: We thank the reviewer for pointing this out. To address this concern, we show *in vitro* proliferation assays. In our murine glioma models, overexpression of Mek1 or Mek2 did not result in a significant increase in tumor cell proliferation (Figure 14 of this document). This observation is consistent with the *in vivo* survival data, where baseline survival between control and MAPK/ERK-activated groups did not show a meaningful difference in the absence of immunotherapy, suggesting that MAPK/ERK activation alone does not promote faster tumor growth/shorter survival *in vivo* (Figures 2 and 3 of the manuscript). In contrast, in human glioma cells (AM38), we observed a moderate decrease in proliferation upon knockout of ERK1 or ERK2, supporting the known role of MAPK/ERK signaling in cell proliferation. These data are already included in the Supplementary Figure (Supplementary Figure 9 in the manuscript). In humans, we reported that in the absence of immunotherapy, GBM patients with high p-ERK do not have a worse prognosis than those with low p-ERK (Arrieta et al., Nature Cancer, 2021). While is difficult to generalize from a few examples, it is possible that introducing MAPK signaling in a tumor that does not rely on it might not enhance its proliferation, whereas inhibition of MAPK signalling in a tumor that relies on it, can slow tumor growth.

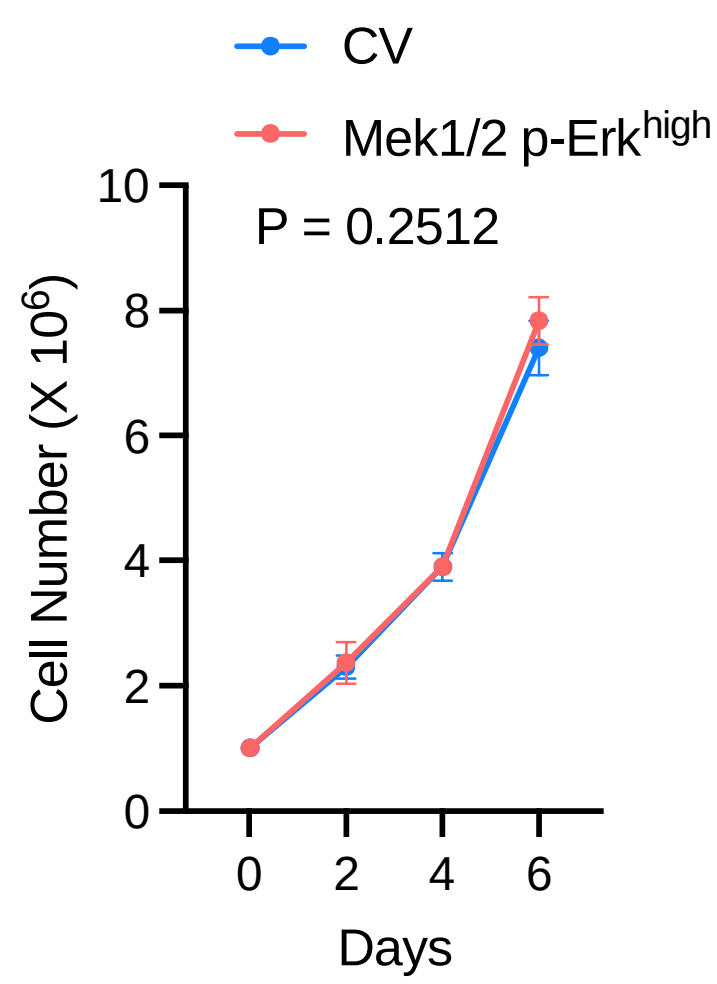

Figure 14. CT-2A Mek1/2 cell proliferation assay.

Comments: Fig. 3. Confirmation with additional glioma models is suggested.

Response: We appreciate the reviewer's suggestion to confirm our findings in an additional glioma model. To this end, we included the CT-2A murine glioma model purchased from Millipore, which in our hands demonstrated responsiveness to immunotherapy. We performed mid-point (21 days post intracranial implantation) flow cytometry analyses and, consistent with our observations in the GL261 model, we observed increased infiltration of $CD8^+$ T cells in tumors with high MAPK/ERK activation. These results support the generalizability of our findings across multiple syngeneic glioma models (Figure 9 of this document). We also have included results that support these central findings using the murine glioma cell line QPP4 (Supplementary Figure 4 of the manuscript). Moreover, we now have included a re-challenge experiment using GL261 wild type cells, where we show that mice that were originally implanted with a MAPK active tumors, and treated with FcE-aCTLA-4, established immune memorty and and mostly resisted engraftment of the parental cell line upon intracranial implantation.

Comment: Fig. 6. Co-culture of tumor cells with T cells to test their antigen presenting functions is suggested.

Response: We thank the reviewer for this suggestion. In this study, we did not claim increased antigen presentation via MHC class I in high p-Erk mouse glioma tumor. Instead, we interpret MHC class II upregulation as a downstream effect of enhanced IFN signaling. As such, we focused on the MAPK/ERK–IFN pathway axis and its role in modulating the immune response. Given this, we do not believe that co-culture of tumor cells with T cell *in vitro* would yield meaningful insights into T cell activation or proliferation in this setting (without additional microenvironment context). To avoid any ambiguity, we have carefully refined the claims of our manuscript text, to avoid stating that MAPK promotes antigen presentation in tumor cells.

Response to reviewers document:

We thank the reviewers for the thorough evaluation of our revised manuscript and the additional comments. Please find below a point-by-point response to these.

Reviewer's Comments:

Reviewer #1 (Remarks to the Author)

The authors addressed all comments of this referee and performed several experiments and recalculations to provide answers. Some of the results acquired to respond to reviewer's quarries which did not support the author's interpretation have been removed. The requested additional information along with a more precise explanation of the results is provided in the revised version. The data are now comprehensive and for the most part the conclusions are in line with the experimental results presented. The new data clarified and enhanced the work reported. Originally identified minor flaws in the data analysis, interpretation and conclusions have been corrected.

The revised version is greatly improved. Regarding comments of other reviewers, I agree with researchers that the data from the ex- vivo slice culture model and an experimental perturbation of MAPK signaling with BRAF inhibition investigated the effect of this intervention on the cross-talk between tumor cells and TAM and provide a causative link. The findings of the manuscript indeed established causal evidence that MAPK drives glioma immunogenicity and response to immunotherapy and explain the results of several clinical trial and human cohort analyses where patients with MAPK exhibit durable responses and favorable survival following immune checkpoint blockade. The results are novel, interesting and important for the field.

Reviewer #2 (Remarks to the Author)

The authors have extensively revised their manuscript, preforming additional experimentation to answer all the reviewers' comments. The data is rigorous, and the conclusions are in line with the data reported. I have not additional concerns.

Reviewer #3 (Remarks to the Author)

We thank the authors for their thorough revision. The stratification of GBM patients likely to respond to immune checkpoint inhibition is a highly relevant topic, and the translational value of this work is significant. In this study, they demonstrate that MAPK pathway genes, known to upregulate p-ERK upon activation, are also regulators of susceptibility to CD8+ T cell activity and anti-PD-1 treatment. These data suggest that cells exhibiting high MAPK pathway activation are more effectively eliminated by T cell-mediated killing. While the authors' revision is extensive and some of the data are compelling—such as the potential for MAPK/ERK signaling to predict responses to various forms of GBM immunotherapy—there remain concerns regarding methodology and the limited integration with existing literature, **which may lead to contradictions with published data**.

1. The overexpression of MEK genes via lentivirus in the hypermutated GL261 model may itself induce a T cell response, likely activating T cells and sensitizing them to PD-1 immunotherapy due to artificial overexpression of antigens. This limitation should be discussed in the manuscript.

Response:
We thank the reviewer for raising this important point. We agree that the GL261 model has limitations, particularly because it is a chemically induced and hypermutated murine glioma line, which can exhibit elevated baseline immunogenicity. Importantly, by the way, GL261 (validated by STR profiling and confirmed to be *Mycoplasma*-free) did not show intrinsic responsiveness to PD-1 blockade in this research and our previous reports (Dmello et al., Nat. Comm. 2023; Kim et al., Neuro-Oncol. 2024), and our MEK-overexpression findings should be interpreted in this context (Figure 2D). Moreover, please consider the following:

- There is no animal model that completely recapitulates human GBM. The murine glioma models cited by the reviewer (2341, M34, and RCAS models) also possess intrinsic or model-establishment–related immunogenic features—GFP, luciferase or avian lentiviral elements, Cre delivery, and vector–associated immune activation. Specifically, the clone of GL261 we used does not respond to ICB, and control vector-GL261 tumors exhibit similar median survival in wild-type C57BL/6 and Cd8-knockout hosts, indicating that baseline CD8-dependent antitumor immunity is minimal in this model.
- Identical lentiviral manipulation was used for both Mek1/2 OE and control vector GL261 tumors, so differences in anti-PD-1 responsiveness reflect MEK pathway activation rather than vector-mediated immunogenicity.
- Murine wild-type Mek1 and Mek2 are intracellular self-proteins unlikely to generate artificial neoantigens; therefore, increased T cell infiltration is unlikely to be related to antigenicity of the transgene itself.
- Our findings are consistent across multiple independent models and datasets (CRISPR screen, Cd8-knockout hosts, and most importantly, human GBM specimens), arguing against a lentiviral artifact.
- We implanted wildtype (non-transduced) GL261 cells into mice 120 days after the initial GL261 control vector or Mek1/2 OE tumor implantation. These secondary tumors were rejected, demonstrating the presence of long-term immune memory against native GL261 antigens, not lentiviral components.

2. The data for BRAFi+MEKi presented in Figures 5 and 6 raise significant concerns and do not adequately support the claim of a mechanistic link between p-ERK and IFN

responses in GBM. The reliance on spatial data from a single patient in Figure 5, while replicative, lacks proper controls for BRAFi+MEKi (only TMZ as a comparator). Furthermore, the assertion that inhibiting ERK signaling impairs the induction of IFN response genes conflicts sharply with a recent study by Xing et al. This study demonstrated, through multiple high-fidelity model systems and patient-derived materials, that BRAFi+MEKi treatment actually induces IFN response genes. The authors must cite this study in the introduction and thoroughly discuss the discrepancy within the manuscript. The evidence presented by PMCID: PMC12208339 indicates that BRAFi+MEKi increases MHC class II gene expression in microglia and tumor cells, in a syngeneic mouse model that is not particularly sensitive to immune checkpoint blockade, suggesting an opposite effect to what is reported here. Additionally, PMCID: PMC12208339 indicated that MHC class II expression upregulation does not correlate with ICI response, pointing to other influencing factors, such as immunosuppressive cytokines, that may dampen the ICI response.

Response:

- While Figure 5C-F shows representative images of spatial transcriptomic data of one patient to illustrate the result, the relationship (Figure 5D) between high p-ERK and IFN responses is generated using a larger cohort (n = 26 IDH-wildtype, BRAF-mutant GBM dataset).
- In response to the reviewer, we have now included a non-treated control group in Figure 5. Compared with both the untreated control and the TMZ-treated groups, the BRAFi-treated group exhibits reduced type I and type II interferon response signatures.
- The results of Xing et al. (PMCID: PMC12208339) manuscript are tangential to our study. Xing et al. is an interesting paper that investigates the effects of long-standing BRAF/MEK targeted therapy in gliomas, with results that likely reflect mechanisms of evolution and adaptation in the setting of acquired resistance to these drugs. Given that in Xing et al., the MAPK activity (e.g. p-ERK) is not characterized in tumor cells (particularly in the context of long-standing BRAFi/MEKi treatment and acquired resistance), we find it difficult to compare those results with our study, particularly to understand how MAPK influences immunogenicity. Recurrent tumors after BRAFi/MEKi therapy frequently exhibit reactivated or even heightened MAPK activity. Given that Xing et al. did not analyze MAPK MAPK status or its rebound kinetics in their specimens, we cannot conclude much on how MAPK relates to immunotherapy response from that study.
- We have refrained from implying that MAPK drives tumor antigen presentation and therapeutic efficacy.

3. In Figure 6, while a control arm is included in the multiplex analyses, only a single time point is assessed. As suggested in the initial review, conducting a time-course study and including additional models (e.g., AM38 or other pERK high models or MAPK signaling mutant models) would be beneficial in investigating the temporal effects of BRAFi+MEKi on IFN signature expression. Addressing these discrepancies and incorporating a rigorous discussion of contradicting results is critical.

Response:

- The multiplex IF analysis in Figure 6 is based on human GBM specimens, which we categorized into p-ERK–high and p-ERK–low groups using IHC-based quantification. Because these are fixed clinical samples representing single time points from different patients, a time-course study is neither feasible nor conceptually appropriate.

- In our in vitro systems (AM38 or murine high p-Erk model – QPP7) we relied on CRISPR-based KO of ERK1 or ERK2 to directly investigate these kinases, and avoid off-target effects of drugs. There is no rationale for applying BRAFi/MEKi, as our study is designed to interrogate the direct functional role of ERK phosphorylation itself rather than the pharmacologic consequences of inhibiting upstream MAPK components.

4. The authors do touch on some limitations in their discussion, such as the lack of an adequate immune system in their human slice cultures; however, other limitations should be candidly acknowledged.

Response:
In the discussion, we have explicitly acknowledged in the key limitations of our experimental systems, including: (i) the use of Cd8 KO mice in the CRISPR screen, with a detailed rationale for this design choice and a clarification that compensatory $CD4^+$ or unconventional T-cell responses are minimal based on our prior published data; (ii) the inherent constraints of murine models in fully recapitulating human GBM complexity; and (iii) the limited immune completeness of our $BRAF^{V600E}$ GBM slice culture model, which lacks systemic immune components. These points directly address the reviewer's concerns and demonstrate that we have provided a candid and comprehensive discussion of the limitations of our study.

Additional complexities and discrepancies with existing literature must also be addressed prior to acceptance of this article. As mentioned above, a recent paper (Xing et al, PMCID: PMC12208339) showed that adult BRAF-mutant/fusion GBM exhibits significantly higher levels of PD-L1 than BRAF wild-type GBM, validated by two orthogonal methods. Given the relevance of this patient population to the current study, it should be incorporated into the introduction.

Response:
Several seminal studies performed in large glioblastoma patient cohorts and clinical trials investigated PDL1 expression as a predictive biomarker for anti-PD1 immune checkpoint blockade in glioblastoma, and did not find the expression of this receptor to be predictive of response or survival of glioblastoma to anti-PD1 immune checkpoint blockade (Omuro et al., Neuro-Oncology 2017, Zhao et al., Nature Medicine 2019, Cloughesy et al., Nature Medicine, 2019, among others). Thus, this observation by Xing et al., is tangential and irrelevant to the focus of our study, which investigates why pERK is predictive of response and survival to this form of immunotherapy in glioblastoma.

The authors should also discuss the implications of robust PD-L1 expression in BRAF-mutant GBM, which may even surpass that of p-ERK. A comparative discussion of how PD-L1 stands as a predictive biomarker for PD-1/CTLA-4 immune checkpoint inhibition versus pERK is warranted. Xing et al. found that BRAFi+MEKi treatment amplifies PD-L1 expression and induces IFN expression in CD4 T cells, supporting concurrent therapy with anti-PD-1 and anti-CTLA-4. Notably, in mouse glioma models, the combination of these four drugs resulted in greater survival benefits compared to individual drug combinations, even in BRAFi+MEKi resistant glioma. As previously noted, in therapy-resistant models, there is consistent upregulation of IFN and HLA genes with BRAFi+MEKi, fundamentally contradicting findings presented here. Moreover, this upregulation does not yield survival benefits across all models, suggesting that factors beyond MHC class II-mediated antigen presentation must be considered to predict beneficial responses to ICI.

Xing et al., place substantial emphasis on PD-L1 expression; however, PD-L1 is only one of several IFN-responsive genes, and we provide a comprehensive analysis of IFN response. While we do not dispute that PD-L1 can be upregulated, as mentioned above, several seminal studies have demonstrated that PD-L1 expression is not predictive of ICB response in GBM. In contrast, elevated p-ERK levels have been shown to more robustly predict response to PD-1 blockade in GBM, which is the central focus of our study. We do not imply that MHC-class II expression in tumor cells equals antigen presentation, or that this is a biomarker for immunotherapy response.

The findings presented by the authors and those in PMCID: PMC12208339 are sharply contradictory. Xing et al. clearly demonstrated the upregulation of IFN signatures in multiple systems following BRAFi+MEKi treatment, with these signature expressions being time-dependent. They further established that either concurrent treatment with BRAFi+MEKi and ICI or sequencing ICI prior to BRAFi+MEKi treatment yields better outcomes than either treatment alone (or administering ICI after BRAFi+MEKi). The publication of this manuscript could potentially create confusion regarding the impact of these inhibitors on IFN signatures and complicate future research and clinical trials.

- As discussed above, the analysis of Xing et al., is interesting, but focused on the mechanisms of adaptation/evolution following chronic BRAF/MEK inhibition. As it is known that some of these mechanisms can lead to refractory up-regulation of MAPK activity, and MAPK signaling per se is not investigated by Xing et al., it is difficult for us to discuss that study in the context of our analysis of how MAPK modulates immune response in GBM. Indeed, a major reason the two studies cannot be directly compared is the difference in treatment time scales. In our human brain slice culture system, we applied BRAFi/MEKi for 7 days, followed by spatial transcriptomic profiling that captured sustained MAPK suppression, the resulting changes in IFN response signatures, and even alterations in local T cell abundance.
- Our study does not aim to develop or evaluate therapeutic sequencing strategies for BRAFi/MEKi and ICB. Therefore, the combinatorial treatment framework emphasized by Xing et al. is outside the scope of our work. We do not claim that BRAFi/MEKi treatment modulates IFN responses in a clinically actionable manner. Instead, our focus is on how intrinsic tumor-cell p-ERK levels shape GBM cell states and their interaction with the surrounding immune microenvironment. Notably, we showed previously that p-ERK is a stronger predictor of ICB responsiveness than PD-L1 expression or baseline T-cell abundance.

The authors may choose from several options to mitigate these issues: 1. Remove the data regarding BRAFi+MEKi since they are premature and need additional controls and experimentation; 2. Add additional controls and experimentation (such as more relevant models and investigate additional time points); and 3. Significantly revise statements concerning these data, clearly acknowledging all study limitations (e.g., performed only in slice cultures lacking comprehensive immune components, absence of temporal resolution, etc.) and discuss their findings in the context of established literature, allowing for acknowledgment of differences potentially due to technical limitations. For instance, variations in mouse model selection likely influence results, as does the timing of measurements (past studies by the authors indicate that elevated p-ERK levels are only predictive shortly before PD-1 treatment and exhibit longitudinal fluctuations). The authors previously provided convincing evidence that p-ERK1/2 is a predictive marker for response to anti-PD-1 immunotherapy in recurrent glioblastoma. Building on this foundation, the current paper supports the idea of screening recurrent GBM for p-ERK

levels and treating patients exhibiting high p-ERK levels with anti-PD-1. This approach remains encouraging.

Furthermore, while they previously associated increased p-ERK levels with greater microglia frequency and enhanced MHC class II gene expression on these cells correlating with increased responses to PD-1, they should also explore alternative mechanisms through which MAPK pathway activation could affect microglial behavior (such as the secretion of microglia-recruiting cytokines), as demonstrated by Kocher et al. (PMCID: PMC11147834) in the BT40 model, which, despite not being GBM, exhibits high-grade glioma features.

Response:
In response, we have enhanced our results as the slice culture data we provide in the revised manuscript by including a non-treatment control condition, which supports our results. As mentioned above, we have provided a thorough acknowledgment of the limitations of our study in the discussion of our manuscript. We disagree that our results are not generalizable due to the use o GL261, as we used multiple mouse tumor models (GL261, CT-2A, QPP7), and all findings that we report were also observed in the various human analyses we include in the manuscript.

With regards to the reviewer's request to perform experiments on slice cultures or cell lines over multiple timepoints, we would like to clarify that this is unfortunately not feasible, as we would need another human GBM with BRAF mutation freshly isolated to conduct these studies. These represent less than 3% of all GBM. We also do not consider these multiple time points to be high yield for our study. Our analyses were all done in relatively short timeframes following MAPK inhibition, or with stable KO or over-expression clones that provide consistent p-ERK modulation. We also provide evidence that when Mek1/2 over-expressing clones lose pERK over multiple passages, the resultant tumors become resistant to ICB. For each of our models, we have characterized MAPK signaling after experimental perturbation to attribute the phenotype to this pathway. Thus, the findings of our manuscript establish causal evidence that MAPK drives glioma immunogenicity and response to immunotherapy and explain several clinical trial and human cohort analyses where we observed MAPK to identify patients that exhibit durable responses and favorable survival following immune checkpoint blockade.

Kocher et al. (PMCID: PMC11147834) investigate the cytokine secretion in pediatric glioma models during BRAF/MEK inhibition that remains elevated shortly after discontinuation of this therapy, which is tangential to our study. We provide data on cytokine expression that can be attributed directly to MAPK signaling, which is conserved across preclinical *in vitro* and *in vivo* models, as well as human GBM slice culture models. The MAPK-related tumor cell microglia crosstalk we describe is confirmed through orthogonal spatial transcriptomic analyses in non-overlapping human GBM sample sets. In Figure 7, our network analysis demonstrates that microglia interacting with control tumors exhibited inflammatory phenotypes; however, these microglia–tumor interaction networks are lost following MEKi/BRAFi treatment, indicating MAPK-dependent regulation of microglial states. To further support this observation, our *in vitro* experiments show that high p-Erk tumor cells actively maintain pro-inflammatory features in surrounding myeloid cells.

Reviewer #4 (Remarks to the Author)

The manuscript has been improved in this revision. However, the authors have not addressed most of the key concerns raised previously.

1) **As commented by both Reviewers #3 and #4, GL261 is not a good model studying glioma immunology. The key in vivo results in Figures 2 and 3 need to be validated using additional animal models.**

Response:
Our conclusions are not dependent on GL261 alone. The key findings shown in Figures 2 and 3 are supported by multiple independent in vivo systems, including CT-2A, QPP7 glioma models; $BRAF^{V600E}$ human brain slice culture system; human GBM datasets (high vs low p-ERK samples); MERFISH dataset from human GBM patients, and large datasets of single-cell RNAseq of human GBM, cell lines, etc. Thus, the central in vivo observations are validated across diverse model systems, and the reliance on GL261 does not drive or bias our overall conclusions.

2) **The overall findings are relatively not novel. The advance over the previous literature (from others and the same group) is incremental.**

Response:
The fact that MAPK drives susceptibility to T cells and various forms of immunotherapy through enhancement of IFN responses, modulation of microglia phenotype, and an increase in T cell infiltration is novel.

3) **Mechanism study is relatively descriptive. No experimental data have been provided to define how MAPK/ERK activation induces IFN response.**

Response:
We respectfully disagree; how MAPK/ERK regulates IFN responses is out of the scope of this manuscript, which focuses on investigating a causal relationship between MAPK signaling in tumor cells, with the associated microglia phenotype, and susceptibility to immunotherapy. We show that IFN response is one pathway modulated by MAPK, and cite literature that implicates MAPK in response to IFN in other systems.

4) The translational impact is relatively weak. It is still not clear whether tumors in this study are responsive to targeted therapy or resistant, since reactivation of the MAPK pathway in recurrent tumors is one of the resistance mechanisms. It is confusing that this work suggests potential combination of MAPK/ERK pathway activation with immune checkpoint inhibitors to counterbalance the pathway's tumor-promoting effects while enhancing the immune response.

Response:

- The findings of the manuscript establish causal evidence that MAPK drives glioma immunogenicity and response to immunotherapy and explain several clinical trial and human cohort analyses where we observed MAPK to identify patients that exhibit durable responses and favorable survival following immune checkpoint blockade.
- Moreover, in the clinic, patients with BRAF-mutant GBM or HGG routinely receive BRAFi/MEKi.

- Importantly, our work does not propose combining MAPK/ERK activation with immune checkpoint inhibitors. Rather, we are suggesting that MAPK pathway status (whether p-ERK is present, and or whether this pathway is being inhibited with drugs) should be considered when immunotherapies for not only BRAF-mutant tumor but general GBM or HGG tumors.
